# Effect of Ca^2+^ on the promiscuous target-protein binding of calmodulin

**DOI:** 10.1371/journal.pcbi.1006072

**Published:** 2018-04-03

**Authors:** Annie M. Westerlund, Lucie Delemotte

**Affiliations:** Science for Life Laboratory, Department of Applied Physics, KTH Royal Institute of Technology, Stockholm, Sweden; National Cancer Institute, United States of America and Tel Aviv University, Israel, UNITED STATES

## Abstract

Calmodulin (CaM) is a calcium sensing protein that regulates the function of a large number of proteins, thus playing a crucial part in many cell signaling pathways. CaM has the ability to bind more than 300 different target peptides in a Ca^2+^-dependent manner, mainly through the exposure of hydrophobic residues. How CaM can bind a large number of targets while retaining some selectivity is a fascinating open question. Here, we explore the mechanism of CaM selective promiscuity for selected target proteins. Analyzing enhanced sampling molecular dynamics simulations of Ca^2+^-bound and Ca^2+^-free CaM via spectral clustering has allowed us to identify distinct conformational states, characterized by interhelical angles, secondary structure determinants and the solvent exposure of specific residues. We searched for indicators of conformational selection by mapping solvent exposure of residues in these conformational states to contacts in structures of CaM/target peptide complexes. We thereby identified CaM states involved in various binding classes arranged along a depth binding gradient. Binding Ca^2+^ modifies the accessible hydrophobic surface of the two lobes and allows for deeper binding. Apo CaM indeed shows shallow binding involving predominantly polar and charged residues. Furthermore, binding to the C-terminal lobe of CaM appears selective and involves specific conformational states that can facilitate deep binding to target proteins, while binding to the N-terminal lobe appears to happen through a more flexible mechanism. Thus the long-ranged electrostatic interactions of the charged residues of the N-terminal lobe of CaM may initiate binding, while the short-ranged interactions of hydrophobic residues in the C-terminal lobe of CaM may account for selectivity. This work furthers our understanding of the mechanism of CaM binding and selectivity to different target proteins and paves the way towards a comprehensive model of CaM selectivity.

## Author summary

Calmodulin is a protein involved in the regulation of a variety of cell signaling pathways. It acts by making usually calcium-insensitive proteins sensitive to changes in the calcium concentration inside the cell. Its two lobes bind calcium and allow the energetically unfavorable exposure of hydrophobic residues to the aqueous environment which can then bind target proteins. The mechanisms behind the simultaneous specificity and variation of target protein binding is yet unknown but will aid understanding of the calcium-signaling and regulation that occur in many of our cellular processes. Here, we used molecular dynamics simulations and data analysis techniques to investigate what effect calcium has on the binding modes of calmodulin. The simulations and analyses allow us to observe and differentiate specific states. One domain of calmodulin is shown to be selective with binding involving short-distance interactions between hydrophobic residues, while the other binds target proteins through a more flexible mechanism involving long-distance electrostatic interactions.

## Introduction

**Fig 1:**
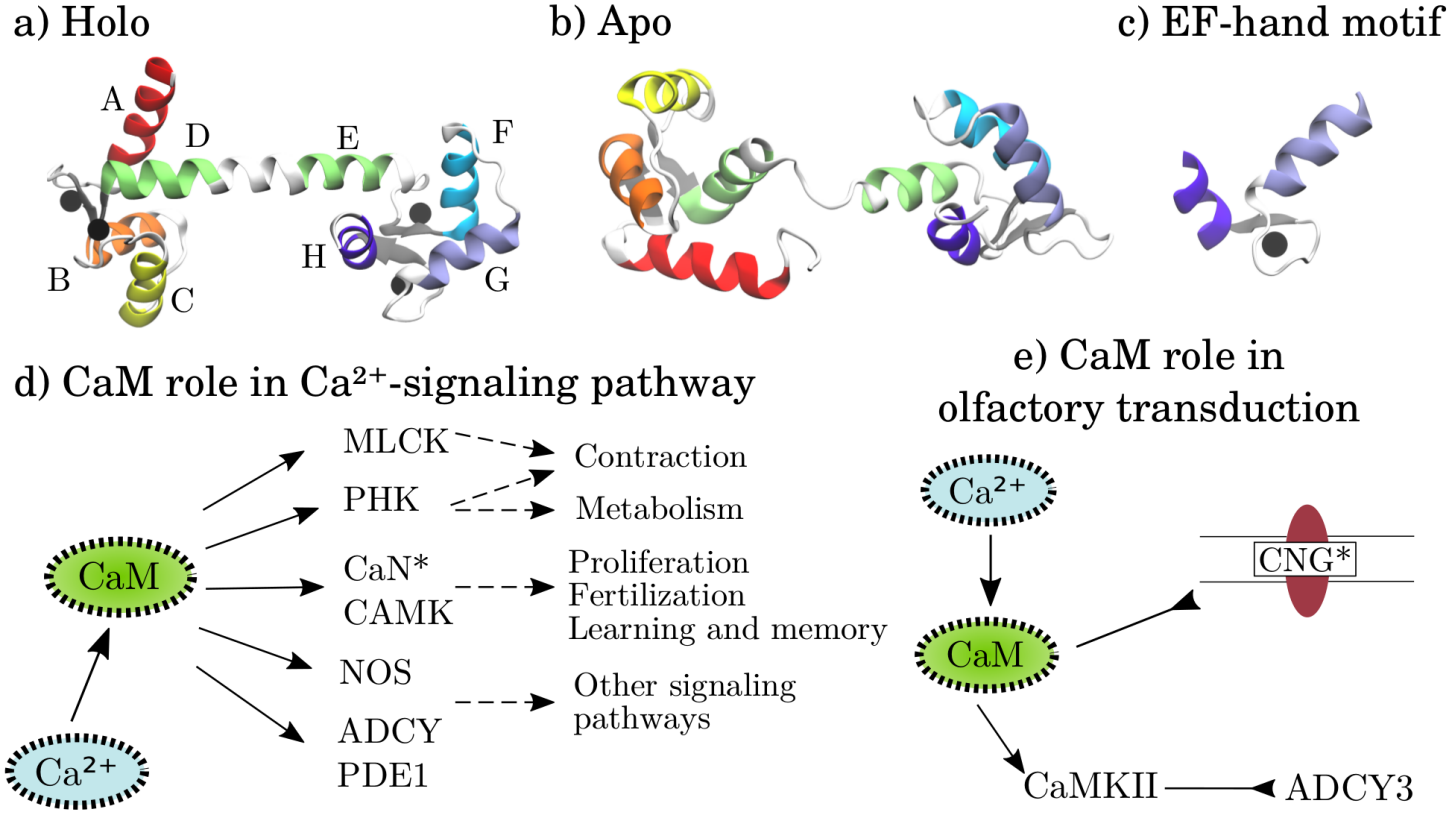
The molecular structure of calmodulin and pathways where calmodulin acts as protein regulator. Molecular structures of a) holo and b) apo calmodulin. The helices are marked according to their canonical labeling. Ca^2+^ ions are represented as black spheres and the beta sheets are marked with gray color. c) The EF-hand motif. d) The role of CaM in the Ca^2+^-signaling pathway. CaM activates the myosin light chain kinase IV (MLCK) and phosphorylase kinase (PHK), calcineurin (CaN), Ca^2+^/calmodulin-dependent protein kinase (CAMK), nitric oxide synthase 1 (NOS), adenylate cyclase 1 (ADCY) and phosphodiesterase 1A (PDE1). This affects downstream processes such as contraction, metabolism, proliferation, learning etc. e) The role of CaM in olfactory transduction. CaM inhibits the cyclic nucleotide-gated (CNG) channel and activates Ca^2+^/calmodulin-dependent protein kinase II (CaMKII). CaMKII then inhibits adenylate cyclase 3 (ADCY3). Proteins marked by a star are included in our CaM binding study. The pathways in d) and e) are adapted from KEGG [3].

Calmodulin (CaM), Fig 1a and 1b, is a promiscuous Ca^2+^-sensing protein that plays part in many physiologically important cellular processes [1]. Its two lobes, connected by a flexible linker, have one beta sheet and two EF-hand motifs each, Fig 1a–1c. The EF-hand binds a Ca^2+^ ion which induces tertiary structure rearrangements of lobe helices, exposing hydrophobic residues [2]. This allows CaM to bind and regulate a myriad of target proteins such as ion channels, kinases and G-protein coupled receptors. The Ca^2+^-signaling and olfactory transduction pathways (Fig 1d and 1e) are two examples of cell-signaling pathways where CaM is involved. In the Ca^2+^-signaling pathway, CaM activates and regulates the myosin light chain kinase IV (MLCK) and calcineurin (CaN), among others [3]. Through this, CaM plays part in regulating a variety of biological processes including metabolism, proliferation and learning. In olfactory transduction, CaM’s role is instead to inhibit the CNG channel and activate the Ca^2+^/CaM-dependent protein kinase (CAMK) which inhibits the adenylate cyclase 3 (ADCY3).

CaM’s promiscuity can be mainly ascribed to its ubiquity and interactions to specific target proteins depend partly on local cell environments such as availability of target proteins. Moreover, high affinity binding to target proteins is partly due to the flexible linker that allows wrapping around target proteins but also stems from the structural properties of the two lobes, thus linked to the pockets formed by their hydrophobic interfaces [4]. Here, we study the interactions between CaM and various target proteins, including proteins that are involved in the Ca^2+^-signaling and olfactory transduction pathways.

**Fig 2:**
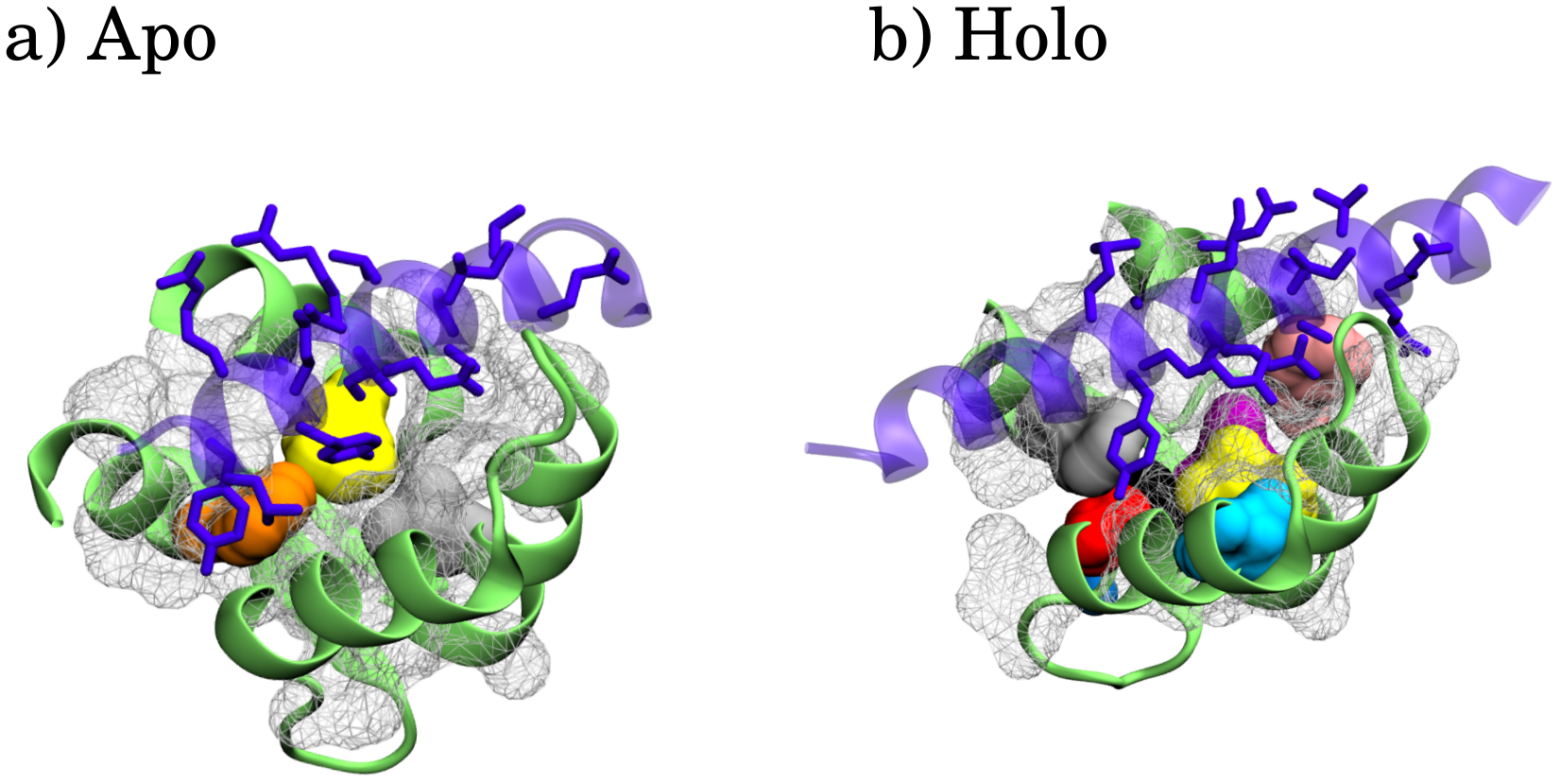
Calmodulin C-term domain binding the voltage-gated sodium channel Na_V_1.2. a) Ca^2+^-free state (apo) and b) Ca^2+^-bound state (holo). The structures have PDB accession numbers a) 2KXW and b) 4JPZ. The target protein backbone is shown as a violet ribbon and its side chains are depicted as violet sticks. Hydrophobic residues are visualized in a white mesh. Key hydrophobic residues found in this paper’s analysis are highlighted in different colors. The residues are shown in S1 and S3 Figs.

Ca^2+^-independent CaM regulation, with Ca^2+^-free CaM (apo CaM) regulating the target protein may occur, although not as frequently as Ca^2+^-dependent CaM regulation. The IQ binding motif, named after the first two conserved residues of the motif, is often associated to apo-CaM binding while 1-5-10 or 1-5-8-14 motifs, named after the position of conserved hydrophobic residues, are associated with Ca^2+^-bound CaM (holo CaM) binding. [5–7] These motifs are, however, lacking the complexity to completely distinguish holo-CaM and apo-CaM binding, as well as different types of binding. An example is seen in Fig 2, where apo and holo CaM C-terminal domain (C-CaM) expose different hydrophobic interfaces, but both bind the voltage-gated sodium channel Na_V_1.2 at the same IQ binding motif. Holo CaM often interacts with a higher affinity, but in some cases, such as the IQ motif of Na_v_1.2, apo-CaM binding is more favorable than holo-CaM binding [8, 9].

Both CaM dynamics and motif-dependent target-protein binding have been extensively studied via both experiments [2, 8, 10–25] and simulations. Indeed, all-atom molecular dynamics (MD) simulations allow for a detailed description with molecular insights. Early MD simulations of the holo CaM conformational ensemble have shown the holo-CaM linker to be flexible [26–28] and indicated structural destabilization when removing Ca^2+^ from one site in the N-term domain [29]. Moreover, CaM target-protein binding was approached by studying the dynamics as well as the thermodynamics of CaM or CaM complexes [4, 26, 30], with insights into binding of specific target proteins using both regular MD and metadynamics [30]. However, little is known about the conformations of the binding interfaces and different binding modes of the two lobes, as well as their relation to protein-unbound CaM conformational ensemble. Part of this was addressed through the analysis of multiple aggregated MD simulations of C-CaM using Markov state modeling (MSM), in which target protein binding was proposed to occur by C-CaM adopting the bound conformation before binding to the protein [31]. However, the analysis did not cover the differences of CaM N-terminal domain (N-CaM) and C-CaM dynamics, mechanisms and binding modes, nor did the study explore the possibility of different binding mechanisms linked to different binding modes.

Binding a ligand can be conceptualized using two frameworks: conformational selection and induced fit. In pure conformational selection, the apo protein adopts a holo-like state before binding [32]. In pure induced fit, the ligand binds in a typical apo state that is not ideal, which causes subsequent rearrangements before reaching a high-affinity holo state [33]. In reality, binding likely involves a combination of the two mechanisms. However, spectroscopy experiments, as well as extensive MD simulations, may shed light on which mechanism is dominating. If the apo protein samples the holo-like state, conformational selection is typically assumed to be dominating, otherwise induced fit is assumed [34].

Here, we analyze thermally enhanced MD simulations of calmodulin with different Ca^2+^-occupancy and use spectral clustering to elucidate calmodulin selective promiscuity. We search for indicators of conformational selection by mapping solvent exposure of residues from sampled states (clusters) to contacts of already existing structures of CaM/peptide complexes. Moreover, we gain knowledge about the characteristics behind different binding modes of the two lobes, as well as the difference between holo and apo binding modes.

## Materials and methods

### Calmodulin system preparation and equilibration

For this project, we considered four different binding states: holo and apo calmodulin, as well as Ca^2+^ bound only in the N-term (N-holo) and Ca^2+^ bound only in the C-term (C-holo), Table 1. The simulations of N-holo, C-holo and holo CaM used structure 3CLN [35], while the apo simulations used structure 1LKJ [36]. N-holo was generated by removing Ca^2+^ from C-CaM and C-holo by removing Ca^2+^ from N-CaM from the holo structure. The systems were built using Charmm-gui [37, 38], where the protein was solvated in a box of about 21000 water molecules. The systems were then ionized with 0.15 M NaCl. Charmm36 was chosen as force field [39], and TIP3P [40] as water model. The modified parameters of Charmm27 force field from Marinelli and Faraldo-Gomez [41] were used to parameterize Ca^2+^ ions.

**Table 1:** Systems and MD simulation details.

Ca^2+^-binding state	PDB ID	Number of atoms	Length [MD, T-REMD, REST]
**Holo**	3CLN	65620	[3600, 570, 460] ns
**Apo**	1LKJ	66277	[5800, 570, 420] ns
**Ca^2+^ in N-term (N-holo)**	3CLN	65614	[2400, 570, 420] ns
**Ca^2+^ in C-term (C-holo)**	3CLN	65614	[2100, 570, 350] ns

5000 steps of minimization were carried out, followed by a 50 ps NVT ensemble equilibration with strong harmonic restraints on the protein atoms. The box was then scaled, relaxing pressure with Berendsen barostat [42], while gradually releasing the position restraints for 350 ps.

### Molecular dynamics simulation parameters

The MD parameters used in these simulations are extensively described elsewhere [43].

Calmodulin was simulated in an NPT ensemble with a 1 atm pressure and 2 fs time step. The short-ranged electrostatic interactions were modeled with a 1.2 nm cutoff where the switching function started at 1.0 nm, and the long-ranged ones with PME [44]. We used a Nose-Hoover thermostat [45], an isotropic Parinello-Rahman barostat [46], and constrained hydrogen bonds with LINCS [47].

The MD simulations were run at a constant temperature of 303.5 K. In addition to regular MD simulations, temperature enhanced simulations were performed; temperature replica exchange [48] (T-REMD) and replica exchange solute tempering [49–51] (REST). In T-REMD, parallel simulations of independent replicas are run at different temperatures. A random walk in temperature space is achieved by employing the Metropolis criterion periodically to accept coordinate exchanges between neighboring replicas. Conformations obtained at higher temperatures are propagated down to the lower temperatures through exchanges. Because barriers are more easily passed at higher temperatures, the efficiency is increased when the free energy landscape is rugged. However, the number of replicas needed to span a certain temperature interval scales with system size [52]. For this reason, T-REMD may not always be efficient. To alleviate this, REST only modifies the hamiltonian of the system for the solute (protein), and not the solvent [51]. However, the relative efficiencies of regular MD, T-REMD and REST depend on the ruggedness of the free energy landscape [43].

We used 25 replicas in both T-REMD and REST. The replicas of T-REMD spanned a temperature range of 299.13-326.09 K, while the REST replicas were simulated at temperatures between 300.0-545.0 K. The temperature ranges for REST and T-REMD were chosen using the “Temperature generator for REMD simulations” [53], considering only the protein for REST. Exchanges between neighboring replicas were attempted every 2 ps, where half of the replicas were involved in each attempt.

The REST simulations were performed with the Plumed 2.3b [54] plug-in patched with Gromacs version 5.1.2 [55], where the charge of the atoms in the hot region were scaled, as well as the interactions between the two regions and the proper dihedral angles [56]. Analysis was performed using the replica at the lowest temperature. and was carried out on the protein heavy atoms. The first four residues in the apo structure were removed, because those are missing in the 3CLN structure.

The apo CaM ensemble is more diffusive and generally well sampled by regular MD, while holo CaM free energy landscape is more hierarchical, and thus sampled more efficiently by temperature enhanced methods [43]. For this reason, more MD data is used in the apo analysis while more REST data is used for holo, Table 1. The three methods may sample different regions with varying efficiency, which is why data from all three simulations is used.

### Identifying states through spectral clustering

**Fig 3:**
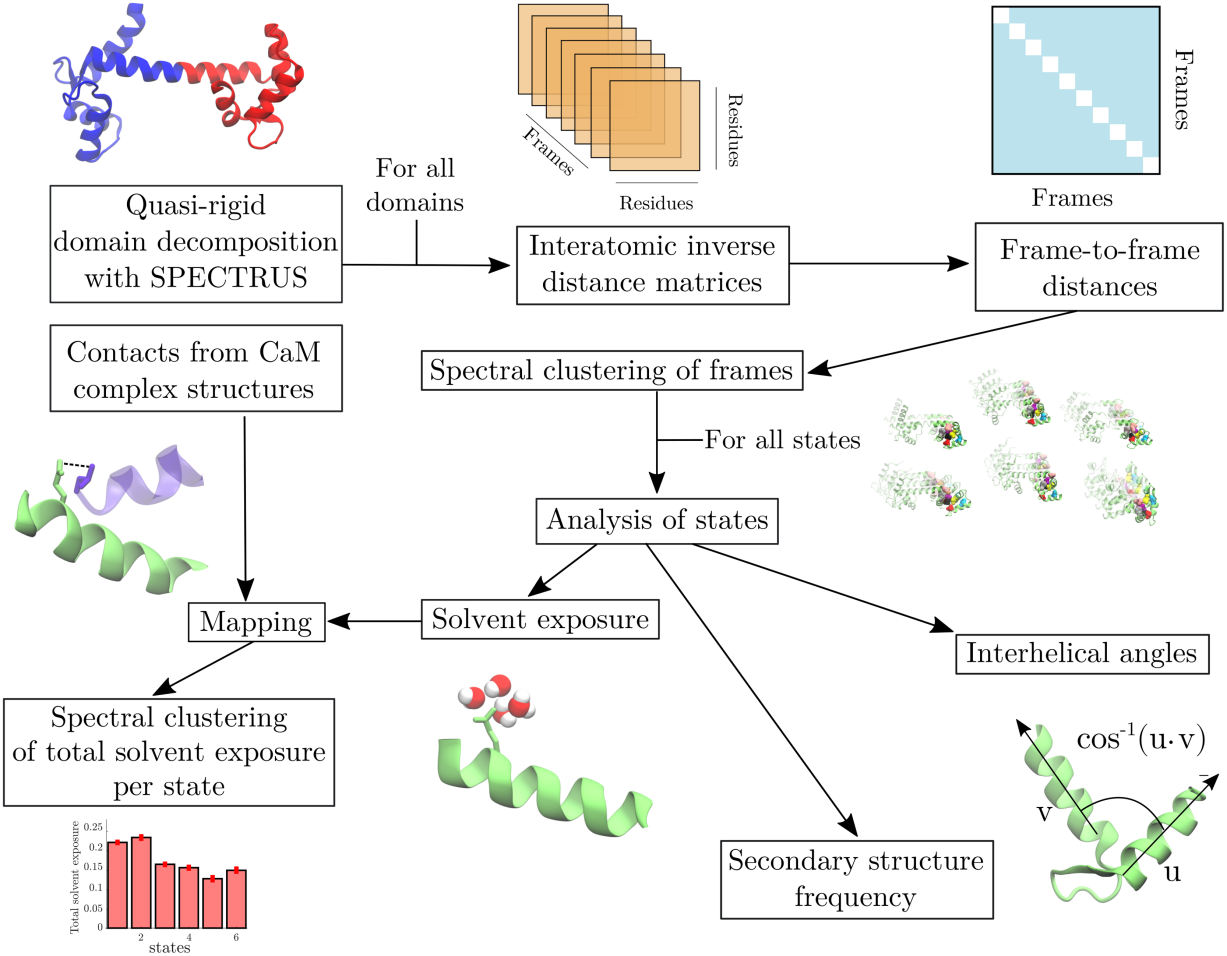
Flow chart of analysis methods. The quasi-rigid domains of CaM were first identified. Then, interatomic inverse distance matrices were used to cluster the frames into states with spectral clustering. The states were analyzed by computing interhelical angles and secondary structure frequencies. Finally, the solvent exposure of each state was mapped to contacts formed in CaM structures, followed by a classification of binding modes with spectral clustering.

Fig 3 illustrates the steps carried out to analyze the MD simulation trajectories.

In a first step, the protein was divided into quasi-rigid domains using SPECTRUS [57]. This procedure exploits fluctuations between residues to determine which parts of the protein move together. The clustering and post-processing described hereafter were done on each quasi-rigid domain.

To further reduce dimensionality and complexity of the dataset while preserving the most important features, we used spectral clustering [58]. The advantage of spectral clustering compared to regular clustering techniques like *k*-means is three-fold. First, it is able to accurately assign points to non-convex clusters. Second, non-linear dimensionality reduction is intrinsic to the algorithm. For high-dimensional data, such as MD trajectories, non-linear dimensionality reduction improves clustering by circumventing the curse of dimensionality where the sparsity of the data increases with increased dimensionality [59]. Third, the number of clusters is the same as the number of dimensions onto which the points are projected. This feature becomes advantageous as the number of free parameters is reduced.

In spectral clustering, the data manifold is first approximated by a graph with adjacency, or similarity, matrix *A*. It contains the local relationships between points and is constructed given the matrix of distances *d*. The distances *d*_*ij*_ are passed through a radial basis function, or Gaussian kernel, with scaling parameter *σ*, yielding the graph edge-weights

$$\begin{array}{c} A_{ij}=e^{-\frac{d_{ij}^{2}}{2\sigma^{2}}}, \end{array}$$ (1)

where *d*_*ij*_ is the dissimilarity between conformation *x*_*i*_ and *x*_*j*_. The geodesic distance between two points is the distance between these points along the manifold, the shortest distance on the graph. The size of the scaling parameter, *σ*, influences the accuracy of geodesic distances, and should not be too large nor too small. A too large *σ* would yield short-cuts, thus causing non-convex clusters to be poorly defined. A too small *σ*, on the other hand, would result in a disconnected graph. Here, *σ* is the average nearest neighbor distance between configurations. The random walk matrix, related to the Laplacian [58], is then constructed

$$\begin{array}{c} L=D^{-1/2}AD^{-1/2}. \end{array}$$ (2)

The degree matrix, *D*, is diagonal with *D*_*ii*_ being the degree of node *i*. The first *k* eigenvectors are computed and normalized per row to obtain points projected onto the *k*-sphere. These points are clustered into *k* clusters using *k*-means with centers projected onto the sphere. The choice of number of clusters is guided by the maximum eigengap, the difference between two consecutive eigenvalues. The representative structure of a cluster is chosen as the structure with smallest RMSD with respect to the other structures in the cluster. A cluster with all structures represents the conformational heterogeneity of one state.

Here, in practice, the dissimilarity between conformations, *d*_*ij*_, is measured as the distance between contact maps of inverse inter-atomic distances (iiad-cmap). This general metric is effective for all proteins and, unlike root-mean-square-deviation (RMSD), does not rely on structural alignment. The inverse distances make larger distances small so that far-away motions are cancelled out, without requiring a cutoff. The frame-to-frame distance matrix is compiled by computing the distances between each frame iiad-cmap.

### Analysis of states

Each state, or cluster of frames, was analyzed to provide statistical information about its specific characteristics and molecular features. The features included interhelical angles, secondary structure and importance of states for target protein binding.

#### Extracting molecular features of states

A common approach to study calmodulin conformations is to compute its interhelical angles [2, 24]. The interhelical angles in part define the exposed hydrophobic interface and thus selectivity. The angle, *α*, between two helices, ***u*** and ***v***, was determined by their dot product, ***u*** ⋅ ***v*** = ‖***u***‖‖***v***‖ cos(*α*). We defined a helix vector as the principal eigenvector of the points corresponding to the heavy backbone atoms in sequence direction. The angles between each pair of helices *A* − *D* (N-CaM and first half of linker) and *E* − *H* (second half of linker and C-CaM) were calculated, following the helix definitions from Kuboniwa et. al [2]. The helix boundaries are shown in Table 2. We compared the angles found with the method used here to the results in [2, 24], as well as with the experimentally obtained structures of protein-unbound holo and apo CaM, Table 3, and CaM bound to target proteins, Tables 4 and 5.

**Table 2:** Residue definition of the calmodulin alpha helices.

Helix ID	Residues in helix
**A:**	6-18 (holo: 13-18)
**B:**	29-38
**C:**	45-54
**D:**	65-74
**E:**	83-91
**F:**	102-111
**G:**	118-127
**H:**	139-145

**Table 3:** Experimental structures of calmodulin used to compare interhelical angles with the MD dataset.

PDB ID holo	PDB ID apo
2K0E [60]	1LKJ [36]
1CLM [61]	1DMO [24]
1CLL [62]	1CFC [2]
1EXR [63]	
1OOJ [64]	
1OSA [65]	
1PRW [66]	
1UP5 [67]	
3CLN [35]	
4CLN [68]	
4BW7 [69]	
4BW8 [69]	
3IFK [70]	
1AK8 [71]	

**Table 4:** Structure PDB ID and binding mode identified for holo CaM complexes.

PDB ID	Target protein	N-term binding mode	C-term binding mode
**5J03:** [10]	Chimeric Kv7.2-Kv7.2 channel	Deep intermediate	Shallow intermediate
**5DBR:**	Nav 1.5 channel (human)	Shallow	-
**5DOW_*A*_:** [11]	Murine Cl^−^/HCO_3_-exchanger SLC26A3	Shallow	Shallow
**5DOW_*C*_:**	Murine Cl^−^/HCO_3_-exchanger SLC26A3	Deep. MET buried.	Intermediate.
**5DOW_*E*_:**	Murine Cl^−^/HCO_3_-exchanger SLC26A3	Shallow.	Shallow.
**5DOW_*G*_:**	Murine Cl^−^/HCO_3_-exchanger SLC26A3	Shallow.	Shallow.
**5HIT:** [12]	EAG1 channel	Shallow.	Shallow.
**5J8H:** [13]	Elongation factor 2 kinase (eEF-2K)	Deep. ALA and PRO facing cleft.	Shallow.
**5SY1_*C*_:** [85]	STRA6 receptor for retinol uptake	Shallow.	Deep.
**5SY1_*D*_:**	STRA6 receptor for retinol uptake	Shallow.	Deep.
**5T0X:**	ER alpha peptides	Deep. TRP buried.	Deep. LEU and TRP buried.
**1G4Y:** [14]	Small conductance (SK) channel	Shallow	Mid-shallow.
**5K7L:** [86]	EAG1 channel	Deep/intermediate. TRP and PHE facing cleft.	-
**5VMS:**	KCNQ1 channel	Shallow.	Mid-shallow.
**5WSV:** [15]	Myosin VIIa IQ5	Shallow.	Intermediate.
**2M0J:** [87]	Olfactory cyclic nucleotide-gated channel	Deep. TRP buried.	Deep. PHE buried in cleft.
**2M0K:** [87]	Olfactory cyclic nucleotide-gated channel	Deep/intermediate. PHE in cleft.	Deep. TRP buried in cleft.
**2MES:** [16]	PSD95	Deep. CYS buried.	Deep. TYR buried in cleft.
**4AQR_*A*_:** [88]	Ca^2+^-ATPase	Deep. VAL buried.	Deep. TRP buried in cleft.
**4AQR_*B*_:**	Ca^2+^-ATPase	Deep. VAL buried.	Deep. PHE buried in cleft.
**4JPZ:** [17]	Nav 1.2 channel	Shallow.	Mid-shallow.
**4M1L:** [22]	IQCG	Deep. TRP facing cleft.	Deep. ILE and LEU facing cleft.
**4Q5U:** [18]	Calcineurin	Shallow.	Mid-shallow.

**Table 5:** Structure PDB ID and binding mode identified for apo CaM complexes.

PDB ID	Target protein	N-term binding mode	C-term binding mode
**2IX7_*A*_:** [19]	Myosin V	Shallow 1 (hydrophobic)	Intermediate 1.
**2IX7_*B*_:**	Myosin V	Shallow 2 (polar)	Intermediate 2.
**2L53:** [89]	Nav 1.5 channel (human)	-	Intermediate 1.
**4R8G:** [20]	Myosin 1c	Shallow 1 (hydrophobic)	Intermediate 1.
**5WSU:** [15]	Myosin VIIa IQ5-SAH	Shallow 2 (polar)	Intermediate 2.
**3WFN:** [21]	Nav 1.6 channel	-	Intermediate 1.
**2KXW:** [8]	Nav 1.2 channel	-	Intermediate 1.
**4LZX:** [22]	IQCG	Shallow 2 (polar)	Intermediate 1.

DSSP secondary structure assignment [72] frequencies were computed to detect the fraction of time each residue is involved in *α*-helices, *β*-sheets or coils. In order to easily spot differences between the different conformational states, as well as between the four binding states, the difference in secondary structure frequency was compared to a typical holo ensemble. To be able to evaluate the fluctuations in the holo simulations, the typical holo ensemble was represented by one third of the T-REMD trajectory.

A conformational state of CaM was characterized by the average solvent exposure of each residue. Solvent exposure of a residue was calculated as the number of water-oxygens in contact with any heavy atom of the residue. The cutoff was chosen to be 4.5 Å. This was not only done for each state, but also for the complete holo, apo, C-holo and N-holo ensembles, respectively. To characterize allosteric communication between the two lobes, we calculated the residue solvent exposures of C-holo and N-holo compared to holo and apo ensembles. To obtain the relative solvent exposure of a state, the cluster-specific solvent exposure was divided by the average solvent exposure of all states.

Distances and DSSP secondary structure assignment were calculated with MDtraj [73], and molecular visualization was done with VMD [74].

#### Kinetic analysis

We calculated the average time taken to transition from one state to another in the MD simulations. The kinetic rates are taken as the inverse of this average time.

#### Propensity of states to exhibit conformational selection

We studied the involvement of the different states (clusters) in binding various targets in a conformational selection mechanism by comparing them with CaM in complex with targets. This analysis required four steps: 1) defining the CaM residues in contact in the different available CaM-complex structures, 2) computing the relative solvent exposure of each residue for the different sampled states, 3) mapping these two results, and 4) classifying different binding modes. These steps are depicted in the bottom left part of Fig 3.

A contact in CaM-complexes was defined as a CaM-residue heavy atom being within 4.5 Å of a target-protein residue atom. For an ensemble of structures, the residue pair were said to be in contact if they were within 4.5 Å for 70% of the structures. A contact vector, *m*^(*k*)^, was devised for each complex, *k*, marking CaM contact residues with ones and CaM non-contact residues with zeros,

$$\begin{array}{c} m_{j}^{\left(k\right)}=\{\begin{array}{c} 1\;\text{if}\;\text{residue}\;j\;\text{is}\;\text{in}\;\text{contact}\;\text{to}\;\text{target}\;\text{protein}\;\text{in}\;\text{structure}\;k\\ 0\;\text{otherwise} \end{array}. \end{array}$$ (3)

If conformational selection dominates the binding to a target protein, the CaM residues in contact to this protein should be exposed to solvent when CaM is unbound. Therefore, the relative solvent exposure, $s_{j}^{\left(i\right)}$, of residue *j* in state *i*, was calculated for each residue. The matching score, or total solvent exposure, for a state *i* and complex *k* was computed as the sum over all residues in contact to the target protein,

$$\begin{array}{c} S_{i}^{\left(k\right)}=\sum_{j=1}^{N_{\text{resid}}}s_{j}^{\left(i\right)}m_{j}^{\left(k\right)}. \end{array}$$ (4)

This was normalized over states to provide a distribution for each CaM-complex.

To identify patterns of different classes of binding modes, these distributions were clustered. For this, spectral clustering was performed again, taking the distance matrix *d* to be the distance between the distributions. For each class, the mean distribution, with standard deviation, of total relative solvent exposure was computed. Moreover, the residue types (polar, apolar, charged) involved in the contacts were identified and the average distribution of residue types as well as the standard deviation were calculated.

## Results and discussion

To characterize the ability of calmodulin to bind to calcium and target peptides through conformational selection, the protocol outlined in Fig 3 was used. The configurations of quasi-rigid domains in molecular trajectories were partitioned with spectral clustering and the obtained conformational states (hereby referred to as states) were analyzed.

### Conformational selection aspects of Ca^2+^-binding in the two lobes

We sampled conformations of apo and holo CaM using MD, T-REMD and REST, Table 1. The analysis began by identifying two quasi-rigid domains, namely N-CaM and C-CaM, with SPECTRUS [57]. The identified domains are shown as red and blue CaM ribbons in Fig 3. The clustering and post-processing were therefore carried out on these two domains separately. S1–S4 Figs show the representative structures of holo and apo CaM obtained after clustering on C-CaM and N-CaM. Together with these states, experimental structures of similar conformations are shown for comparison.

**Fig 4:**
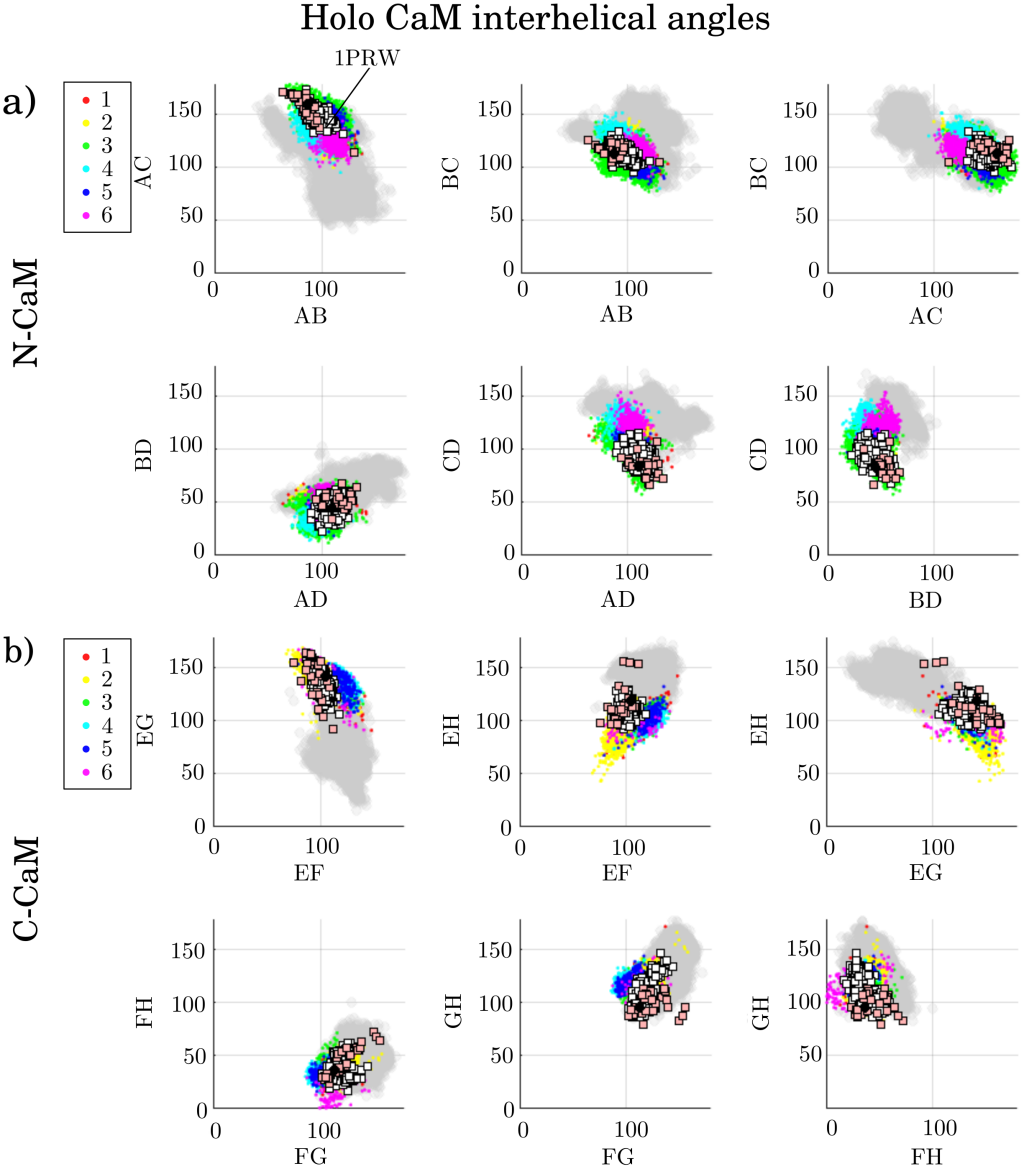
a) N-CaM and b) C-CaM states (clusters) projected onto interhelical angles of holo CaM, colored by their respective assigned cluster. The gray dots correspond to the apo ensemble. The white squares are experimentally determined structures of protein-unbound holo CaM, while pink squares are experimentally determined structures of protein-bound holo CaM. The black circles respresent values of CaM interhelical angles inferred from NMR data [2, 24].

**Fig 5:**
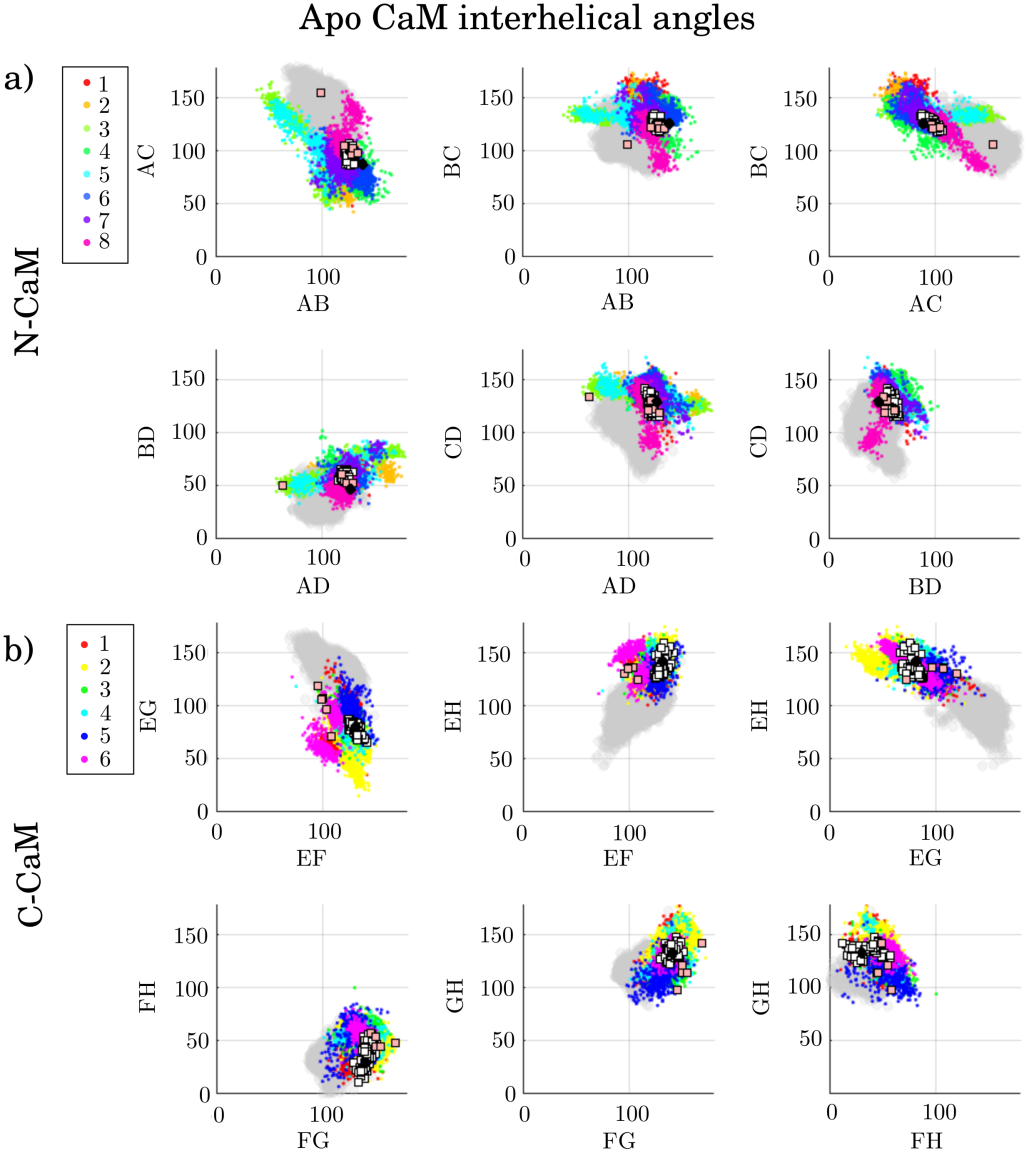
a) N-CaM and b) C-CaM states (clusters) projected onto interhelical angles of apo CaM, colored by their respective assigned cluster. The gray dots correspond to the holo ensemble. The white squares are experimentally determined structures of protein-unbound apo CaM, while pink squares are experimentally determined structures of protein-bound apo CaM. The black circles represent values of CaM interhelical angles inferred from NMR data [2, 24].

The molecular features were extracted from the conformational states by computing the interhelical angles and secondary structures. Fig 4 shows the interhelical angles of each state (cluster) from the holo simulations, while Fig 5 shows the interhelical angles for each state of the apo simulations. Each dot is a projection of a conformation in interhelical angle space, colored according to the state (cluster) it belongs to. On top of this, experimentally obtained structures are plotted as squares. The white squares denote structures where CaM is not bound to target peptides, Table 3, while pink squares correspond to structures with CaM bound to a target peptide, Tables 4 and 5. The black circles are mean values of CaM interhelical angles inferred from NMR data [2, 24], used for comparison. Furthermore, to allow a comparison between interhelical angles in the holo and apo ensemble, the sampled apo conformations are shown as a gray shadow in Fig 4 (holo) and the sampled holo conformations are shown as a gray shadow in Fig 5 (apo). A simple sanity check, S5–S10 Figs, showed that most states were sampled by all three simulation methods, with a few exceptions. Examples of such are states that are separated by a high free energy barrier and therefore not sampled by the regular MD simulations within these timescales, or states that could not be sampled by the replica exchange simulations because of the relatively short time scales simulated. Similar conclusions can be drawn from the kinetic analysis of MD trajectories (S11 Fig).

A change in interhelical angles due to Ca^2+^-binding can be seen in Fig 4 (holo) and Fig 5 (apo), in agreement with previous work [2, 24, 26] (see S1 Text for more extensive description and details). We note also that the conformational ensemble generated by MD simulations is broader than the ones observed experimentally for both protein-bound and unbound CaM. In agreement with NMR studies of holo CaM [60], the protein-unbound CaM adopts the conformations of CaM bound to specific target proteins, which is seen as an overlap between the pink squares and the MD data (colored dots) in Fig 4, thus hinting at a possible conformational selection mechanism of target protein binding.

**Fig 6:**
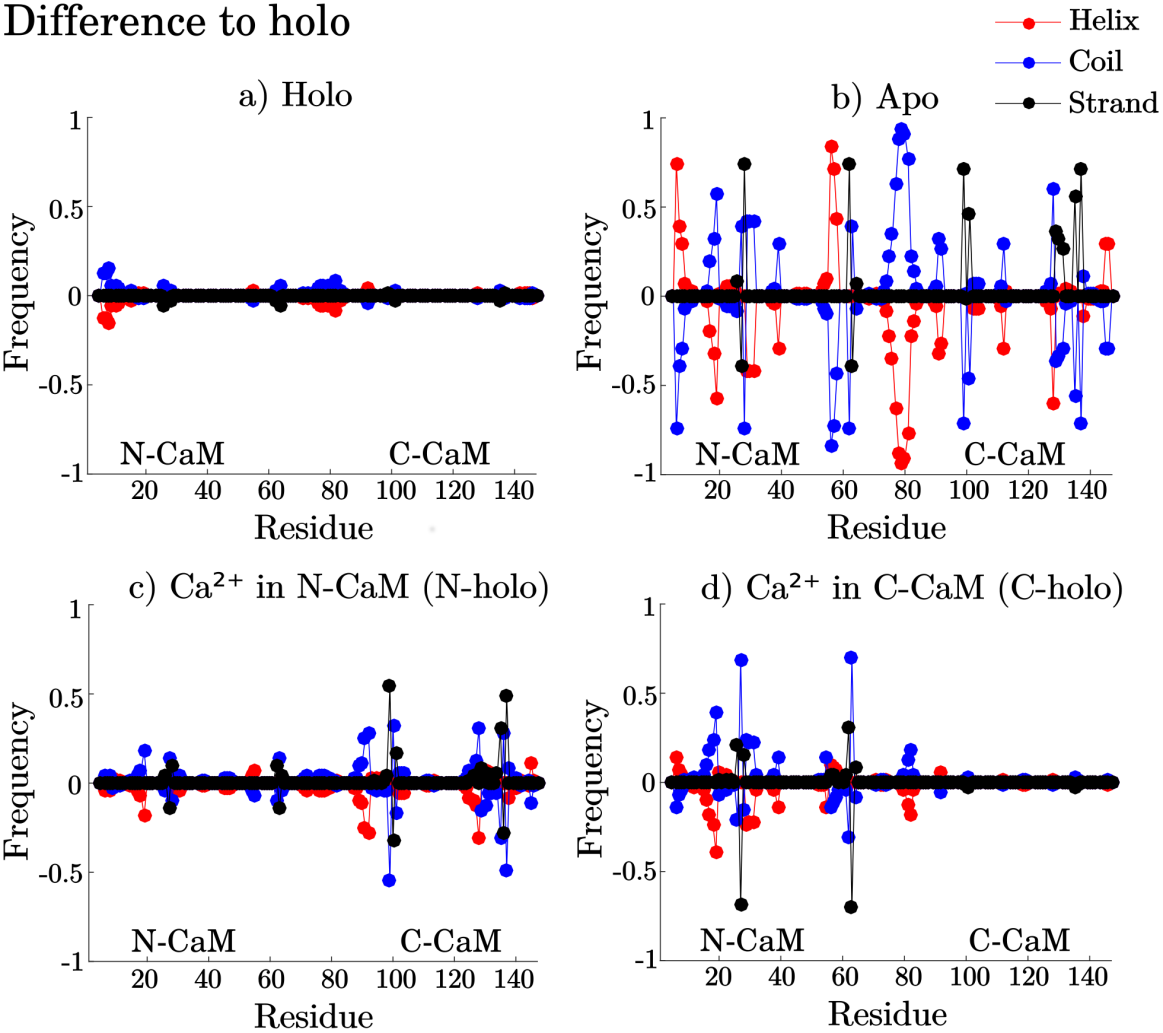
Secondary structure frequency difference to holo for a) complete holo, b) C-holo, c) N-holo and d) apo ensembles. Helix frequency is shown in red, strand in black and coil in blue. Above zero denotes gained secondary structure compared to holo while below shows the loss in secondary structure frequency.

Fig 6 displays the difference in secondary structure frequency for each residue compared to holo. Red dots denote the helical, black the strand and blue the coil content. Positive values indicate a gain in secondary structure element compared to holo, and negative ones a loss. The helical break around residue 74-82 [26–28] is seen to different extents in all Ca^2+^-binding states of CaM, Fig 6. This break occurs in the linker and yields a compact state with the two lobes in contact. This compact state resembles the binding mode where CaM is wrapped around the target protein and was suggested to be an intermediate conformation during target protein- and Ca^2+^-binding [69, 75]. Furthermore, binding Ca^2+^ rearranges the helices in the lobes and pins the beta sheets between residues 27/63 and 100/136 instead of 28/62 and 99/137, Fig 6. Apo CaM is more flexible, allowing the beta sheets to shift between 26-28/62-64 and 99-101/135-137, Fig 6. This highlights the rigidity of holo compared to apo CaM. Due to the flexibility of apo, a C-CaM state (Fig 5b, state 2) is found where the end of the fourth binding loop is involved in a beta sheet, deforming the binding loop, in agreement with [31]. We hypothesize that this state may inhibit Ca^2+^-binding. In this state, residues 129-131 are more prone to form a beta sheet with 99-101 and 135-137, S12 Fig. This extra sheet does not form in apo N-CaM because the coiled loop between helices C and D is slightly shorter than the one in apo C-CaM between helices G and H. The flexibility obtained by removing Ca^2+^ from C-CaM allows these beta sheet formations to occur.

To investigate the possibility for conformational selection during the Ca^2+^-binding process, we searched for potential overlaps in interhelical angle space between apo and holo. We observe holo-like states in apo N-CaM, Fig 5a. (states 3, 5 and 8, plotted with colors lime, cyan and cerise), as well as C-CaM holo-like states that have been reported in previous work [31, 76], Fig 5b (states 1 and 5, depicted as red and blue). These states could potentially aid Ca^2+^-binding though conformational selection. The holo N-CaM states 4 and 6 (shown by cyan and magenta dots) overlap with the apo ensemble, Fig 4a. Their secondary structure frequencies are plotted in S13 Fig, which show similar secondary structure frequencies as apo (Fig 6b), with a shift of beta sheet to residues 28/62. The C-CaM interhelical angles of holo and apo overlap more than the N-CaM interhelical angles do. The overlap in interhelical angles and shift of beta sheet in N-CaM, make these states likely intermediate states in Ca^2+^-binding, involved in conformational selective binding of Ca^2+^.

**Fig 7:**
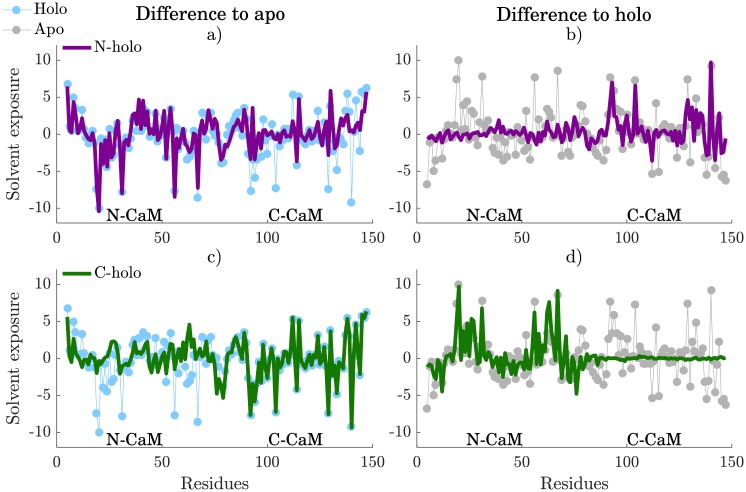
Solvent exposure difference of N-holo (purple) and C-holo (green) to a, c) apo and b,d) holo. The difference in solvent exposure to holo of both N-holo and C-holo are compared to the solvent exposure difference of apo (gray) to holo, while the difference in solvent exposure to apo of the two are compared to the solvent exposure difference of holo (blue) relative to apo.

To assess whether Ca^2+^-binding allosterically modulates the conformational ensemble of the other lobe, we used the MD, T-REMD and REST datasets of C-holo (Ca^2+^ in C-CaM) and N-holo (Ca^2+^ in N-CaM), Table 1. Solvent exposure for each residue in N-holo and C-holo was compared to the apo (Fig 7a and 7c), and holo (Fig 7b and 7d) ensembles. This shows that the Ca^2+^-free lobe tends to transition to an apo-like conformational ensemble, while the Ca^2+^-bound lobe stays in the holo-like ensemble. Although there is a small allosteric influence between the lobes, as seen in Fig 6c and 6d, where N-CaM mostly lacks beta sheet in C-holo, the solvent exposure analysis indicates that the overall conformation of one lobe is mostly independent of the conformation of the other. These results indicate that binding of Ca^2+^ to one of the lobes does not yield a significant population shift in the other lobe and thus that binding is likely not conformationally cooperative between the two lobes. Cooperative binding between sites and lobes has been investigated but the results obtained in different studies have been largely inconsistent, thus making it difficult to validate our results. This is because many different parameter sets in the models used to fit the experimental data perform equally well [77].

### Conformational selection aspects of target protein-binding

To investigate conformational selection aspects of calmodulin binding to target proteins, the relative solvent exposure of the different CaM residues in different states was calculated and mapped to the contacts in a set of CaM complexes, Tables 4 and 5 and Fig 3. The underlying idea is that in a conformational selection mechanism, water molecules around an exposed residue will be replaced by the target peptide. Therefore, the residues that are in contact with the target peptides should be found exposed to solvent in a state involved in a binding mechanism dominated by conformational selection. To then characterize different modes of binding, the distributions of total solvent exposure per state were clustered and divided into classes, as described in the Materials and Methods section.

#### Holo C-CaM complexes can be divided into five classes according to binding depth

**Fig 8:**
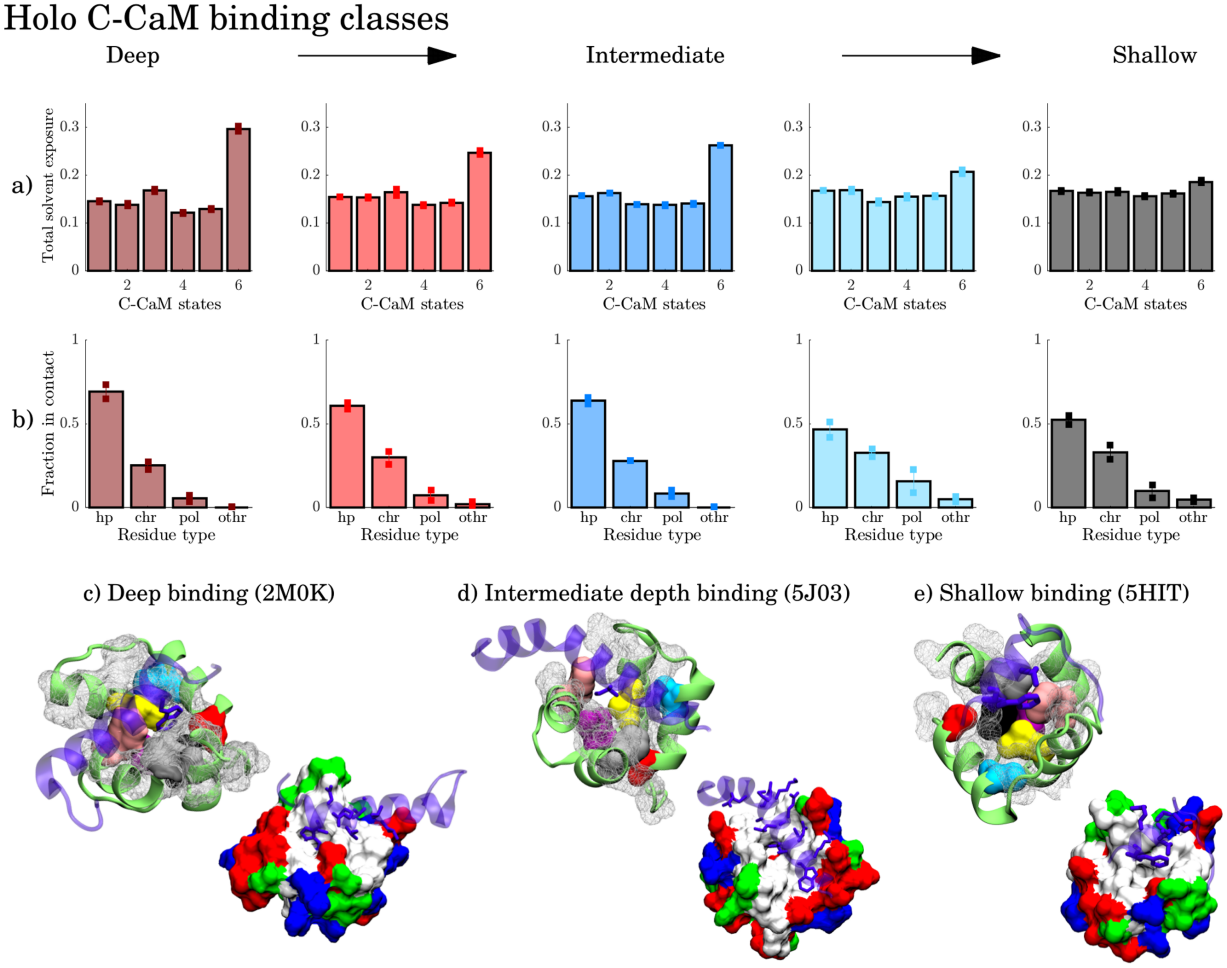
Representative models from the three binding classes of holo C-CaM, with deep binding in c) olfactory cyclic nucleotide-gated ion channel (OLFp), intermediate binding in d) chimeric Kv7.2—Kv7.3 ion channel and shallow binding in e) EAG1 ion channel. Color-highlighted residues are hydrophobic residues showing distinct signals in the contact-solvent exposure analysis. The other hydrophobic residues are shown by the white mesh. Associated to each class is a) the average total solvent exposure distribution per state and b) the average distribution of contact types formed in the CaM-complexes. The standard deviation is indicated on the bars.

The clustering of distributions of total relative solvent exposure of residues involved in binding holo C-CaM complexes (Table 4) yielded five subclasses. Through visual inspection, we aligned these along a depth gradient with deep, intermediate and shallow subclasses, Fig 8, S14 and S15 Figs.

Deep binding is characterized by a large hydrophobic target peptide residue buried in the CaM hydrophobic cleft. It is favored by states 3 and, especially, 6. These states expose hydrophobic residues from deep within the cleft simultaneously; PHE92, LEU105, ILE100, VAL121, VAL136 and PHE141. State 6 is characterized by parallel F and G helices, Fig 4b, and lacking beta sheet structure, S16 Fig. This state, which could be favorable for binding target proteins, is a high free energy state, only observed in the temperature enhanced simulations, S17 Fig. Binding is also likely when CaM is in the state 3 conformation, but binding to state 3 may require more subsequent rearrangements than binding to state 6. An example of deep binding is shown by the contact interface of CaM-CNG ligated ion channel (2M0K) where the a tryptophan is buried in the hydrophobic cleft, Fig 8c. Coincidentally, the residues that give rise to significant signals in relative solvent exposure, S18–S20 Figs and S1 Fig, are also highly conserved in eukaryotes through evolutionary time [78].

Intermediate depth binding specifically favors state 6 and 2, Fig 8. In intermediate depth binding, the residue pointing towards the cleft is smaller than in deep binding, as in the case of CaM-Kv7.2-Kv7.3 (5J03) binding interface with a leucine pointing towards the cleft, Fig 8d.

In the superficial binding class, the residues in contact are roughly equally exposed to solvent in all states, Fig 8a and S18–S20 Figs. As seen in the distribution of residue types that are part of contacts, Fig 8b, less hydrophobic contacts are formed for more shallow binding modes. Moreover, the shallow binding modes include a larger fraction of charged residues, and do not favor any particular state since the residues needed for contact are always equally exposed to solvent. Thus, the long-ranged electrostatic interactions of the charged residues may initiate binding, yielding fast and promiscuous binding, while the CaM hydrophobic pocket of deeper binding may account for selectivity and be involved in binding mechanisms dominated by conformational selection.

#### Holo N-CaM complexes can be divided into two classes according to binding depth

**Fig 9:**
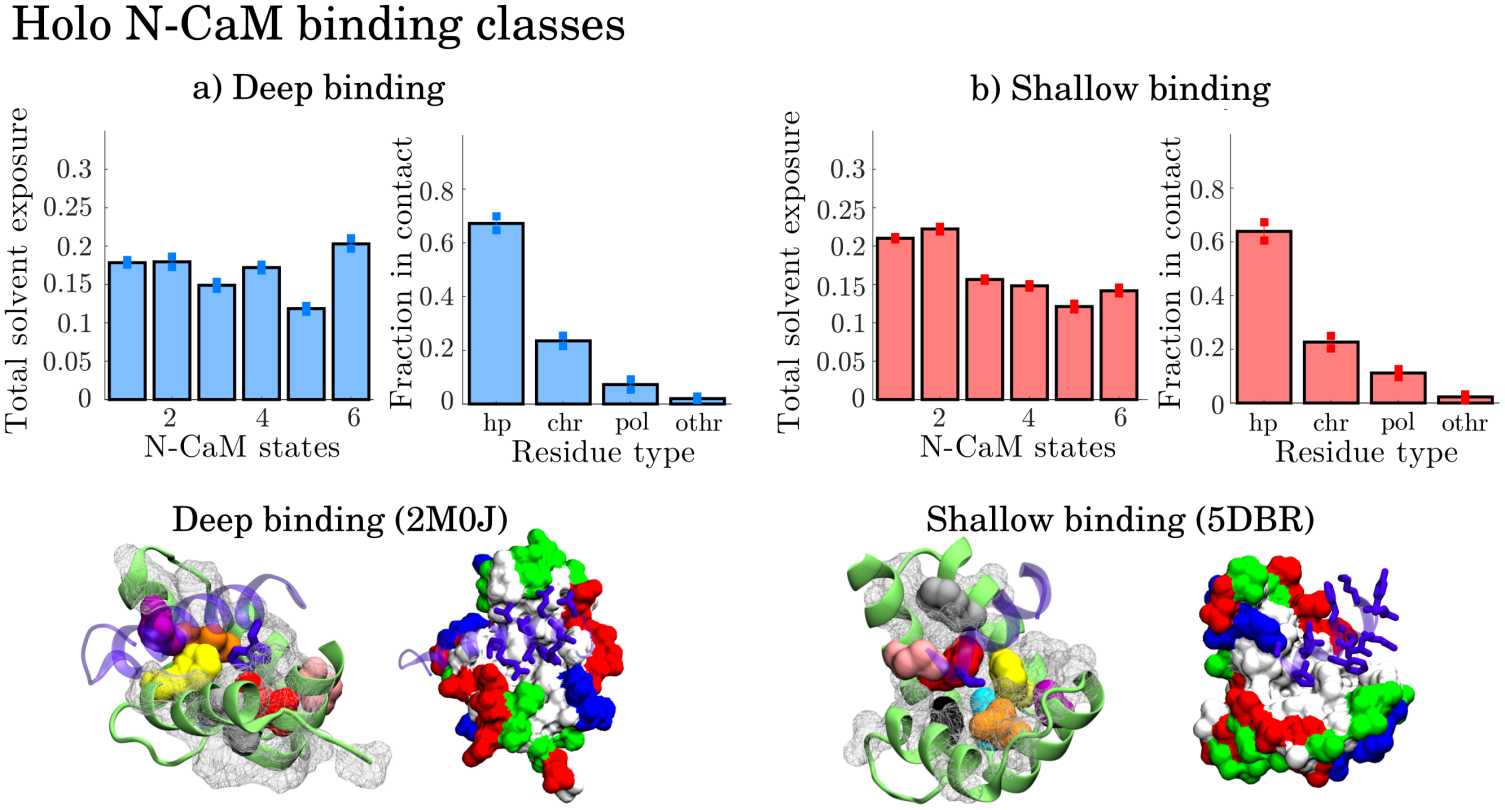
Representative models from the two binding classes of holo N-CaM, with deep binding in a) olfactory cyclic nucleotide-gated ion channel (OLFp) and shallow binding in b) (human cardiac) Nav1.5 ion channel. Color-highlighted residues are hydrophobic residues showing distinct signals in the contact-solvent exposure analysis. The other hydrophobic residues are shown by the white mesh. Associated to each class is the average total solvent exposure distribution per state (left) and the average distribution of contact types formed in the CaM-complexes (right). The standard deviation is indicated on the bars.

For holo N-CaM binding, clustering enabled to identify 2 classes corresponding to deep binding and shallow binding, Fig 9, S21 and S22 Figs. In shallow binding, states 1 and 2 appear more favorable than the rest. The key feature of shallow binding resides in smaller hydrophobic target protein residues facing the CaM hydrophobic cleft, Fig 9b.

For deeper binding, a mix of states gives exposure to all necessary contact residues, namely states 1, 2, 4 and 6, Fig 9a. State 6 is slightly more favorable for complexes with deeper binding, associated with more hydrophobic contacts S21 Fig. However, since not one state exposes all necessary residues, the binding of the N-CaM could be dominated by induced fit. After binding to any state, the rest of the residues necessary for binding will be exposed to reduce free energy. In state 4, CaM binds its own helix A, S2 Fig. This state has been observed before with N-CaM forming salt bridges to the linker [79]. It is also represented by structure 1PRW [66] which overlaps state 4 in the interhelical angle space, Fig 4a. It may display a possible intermediate conformation during a flexible binding process in the N-term.

The hydrophobic cleft of N-CaM is not as open as in C-CaM. This is reason to believe that while C-CaM binding may be dominated by conformational selection, N-CaM binding may need a more flexible mechanism with intermediate states to facilitate deep binding. Also here, however, deep binding involves a large hydrophobic target protein residue buried in the CaM cleft, Fig 9a.

As in C-CaM binding, the depth of binding in the N-CaM hydrophobic cleft determines which interacting residues and therefore which states are more favorable for binding. The contacts in deeper binding are formed through a combination of CaM residues ILE27, LEU32, VAL35, ILE63, PHE68 and MET71, S23–S25 Figs and S2 Fig. These contacts are here associated to a large hydrophobic residue from the target peptide being buried or facing the hydrophobic cleft. Furthermore, these CaM residues are also reported as highly conserved in eukaryotes. [78]

In summary, states 1 and 2 expose residue PHE68 and MET71 which are required even in shallow binding. Therefore, state 1 and 2 are presumably favorable for initiating binding to both shallow and deeper binding modes. State 6 exposes residues in contact in slightly deeper binding and state 4 is probably an intermediate state visited in on the way towards deep binding.

#### Apo CaM target-protein binding is more shallow than holo CaM binding

**Fig 10:**
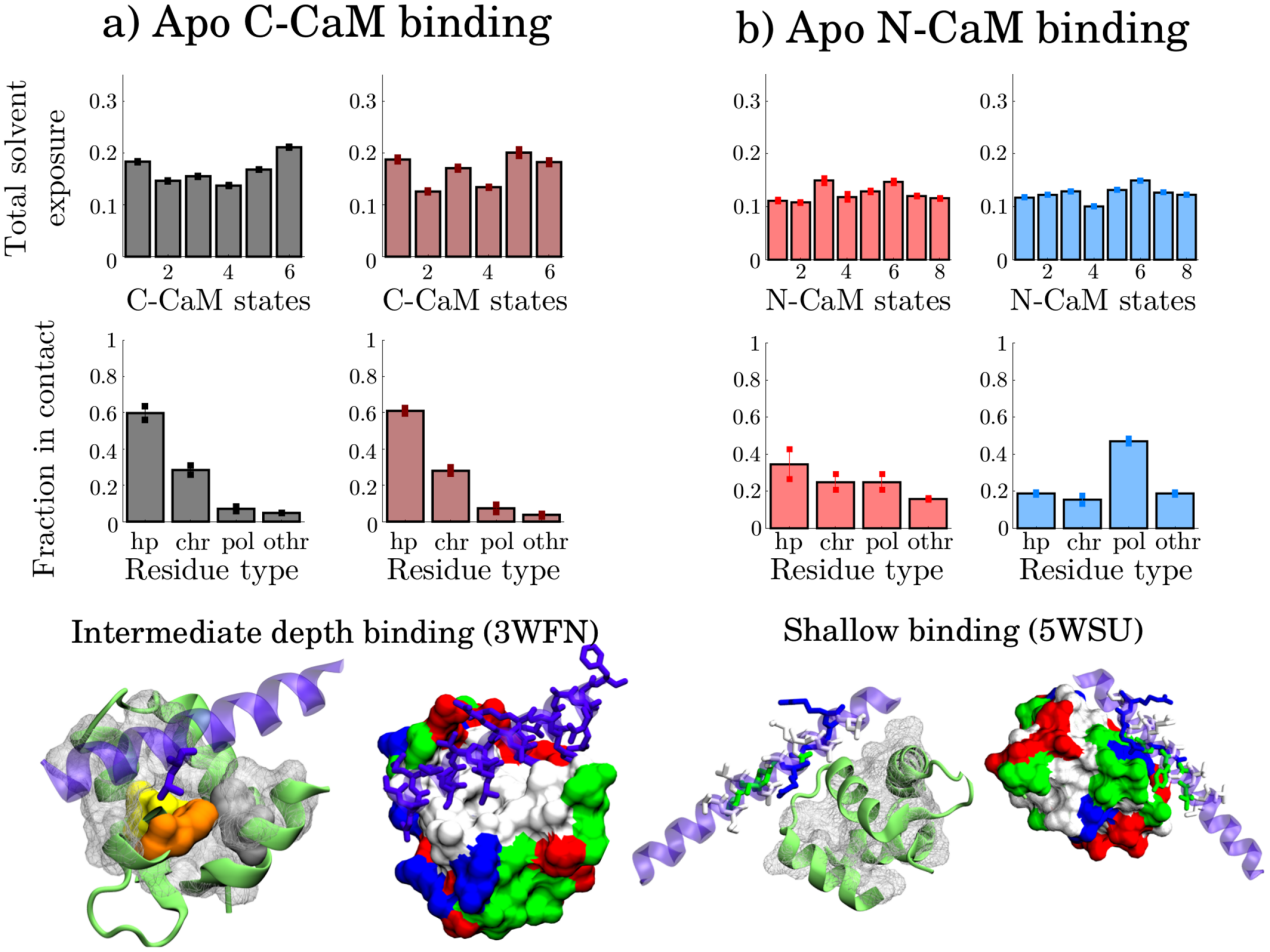
Representative models from the binding classes of apo C-CaM and N-CaM, with C-CaM intermediate binding to a) Na_V_1.6 ion channel and N-CaM shallow binding to b) Myosin VIIa. Color-highlighted residues are hydrophobic residues showing distinct signals in the contact-solvent exposure analysis. The other hydrophobic residues are shown by the white mesh. Associated to each class is the average total solvent exposure distribution per state (upper row) and the average distribution of contact types formed in the CaM-complexes (lower row). The standard deviation is indicated on the bars.

Fig 10a and S26 Fig show the classification of apo C-CaM binding. In this case the classes are divided according to whether contacts are formed with CaM residue LEU105 or PHE141 (in which case state 5 is favored) or with PHE89 (in which case state 6 is favored), Fig 10, Table 5 and S27 Fig. These residues are more exposed to solvent in the states that favor binding to the analyzed target proteins, suggesting that they are important for C-CaM binding.

Interestingly, mutating CaM either at PHE89 and PHE141 to LEU causes arrhythmias and long QT syndrome [80–82]. Moreover the Ca^2+^ affinity has been shown to decrease for the PHE141 to LEU mutation [83]. This residue is, in fact, more exposed in state 1 and 5 (S3 and S27 Figs), the two previously identified holo-like states, Fig 5, which indicates that this residue is important for both calcium as well as target protein binding.

As in holo, apo C-CaM target protein binding involves a larger fraction of hydrophobic residues than apo N-CaM binding. When binding Na_V_1.6 (3WFN), for example, a LEU is buried in the CaM hydrophobic cleft, Fig 10a. In the structures of apo N-CaM binding, however, we only observe superficial binding, Fig 10b and S28 Fig. An example is binding to Myosin VIIa (5WSU), where a VAL is facing the cleft, Fig 10b. The two classes from clustering instead distinguish whether polar contacts or more hydrophobic contacts are formed. The hydrophobic residues are well packed within N-CaM and a lack of hydrophobic cleft characterizes shallow binding.

#### Comparison of apo and holo target peptide binding

For apo CaM binding, the residues that bind to target proteins are in general equally exposed to solvent in all states, S27 and S29 Figs. Therefore, apo does not need to stay in distinct states for binding, but uses its configurational flexibility to adjust the binding pose after initial contacts are made. This flexibility that stems from the low free energy barriers and diffusivity of apo CaM [43], is possibly linked to apo not showing a diverse set of binding interfaces. It has previously been proposed that C-CaM in N-holo is semi-open [25], which would facilitate binding to target proteins, but later it was shown that the semi-open state exists in even pure apo C-CaM [2, 24, 31] and apo C-CaM complexes [8]. Here, the semi-open state is shown to exist in the unbound apo C-CaM ensemble, whereas the N-CaM ensemble is mostly closed. This is in line with the finding where the apo C-CaM interhelical angles overlap holo more than the interhelical angles of apo N-CaM, Fig 5.

Despite the overlap of interhelical angles, and thus semi-openness of apo C-CaM, the true open state and deep binding with a large hydrophobic residue buried in the CaM hydrophobic pocket is an effect of Ca^2+^ and thus only observed in holo CaM. Binding to the protein is then most likely facilitated by the holo C-CaM cluster 6, where the loss of beta sheet opens the cleft and exposes a larger hydrophobic pocket, allowing for deeper binding, S1 and S16 Figs and Fig 4. These well-defined states of holo allow for binding to target peptides in a mechanism dominated by conformational selection.

### Conclusions

We used molecular dynamics and temperature enhanced MD with an agnostic spectral clustering scheme to search for conformational selection of CaM Ca^2+^ and target protein binding. We found that the Ca^2+^-state of one lobe does not significantly influence the conformation of the other lobe, but binding Ca^2+^ may occur through conformational selection facilitated by a transition state that is visited by both apo and holo CaM. It is observed here by overlapping conformations in interhelical angle space. In N-CaM, the transition between apo and holo likely involves a beta sheet residue shift.

The target protein binding modes of apo CaM are more shallow than those of holo CaM, and the charged interactions dominate due to the induced burial of the hydrophobic binding interface from Ca^2+^-depletion. The residues involved in binding are equally exposed in apo CaM states and thus all observed states are equally likely to initiate binding to the target protein.

The notion that not only the linker, but also the binding interface of C-CaM shows configurational flexibility has previously been proposed both through extensive MD simulations [31], but also in structural studies [2, 24, 25]. The conformation of C-CaM interface is suggested to vary more than the N-CaM interface [25]. Here, holo C-CaM shows distinct states exposing hydrophobic residues that are otherwise screened from water. Such states are absent in N-CaM, which also exhibits less binding heterogeneity than C-CaM in the CaM-complex structures. This is seen as the shallow class of C-CaM show more shallow binding than the shallow class of N-CaM, while the deep binding class of C-CaM shows deeper binding than the deep binding class of N-CaM. The distinct states of holo C-CaM with a clear mapping to bound structures indicate a tendency for C-CaM binding to be selective and dominated by conformational selection, while N-CaM, which lacks this clear mapping, likely binds through a more flexible mechanism involving intermediate states.

For general protein-ligand binding, strong and long-ranged interactions have been thought to favor binding dominated by induced fit, while short-ranged interactions would favor conformational selection. [84] Our results support and extend this idea to CaM by showing that weak hydrophobic interactions dominate deep binding modes which indeed tend to be dominated by conformational selection in C-CaM. In the flexible binding of N-CaM, on the other hand, hydrophobic interactions occur less frequently. We hypothesize that the long-ranged electrostatic interactions of the N-CaM charged residues may initiate fast binding while the hydrophobic pocket in C-CaM may account for selectivity. Previously published studies using NMR [23] proposed that C-CaM is selective while N-CaM binds afterwards through induced fit mechanism, or a coupled conformational selection mechanism initiated by C-CaM [60]. This is in line with the argument given here on C-CaM selectivity and N-CaM flexibility.

This study opens the way towards understanding the process and mechanisms behind calmodulin Ca^2+^-sensing. In a next step, the second aspect of binding, induced fit, may be studied. The full mechanism of specific target peptide binding could also be simulated starting from the conformational selection state of CaM identified here.

## Supporting information

S1 Fig The holo C-CaM states. a) The holo C-CaM states representative structures obtained from spectral clustering. Key residues from the contact/solvent exposure analysis are highlighted in colors. b) Experimentally obtained structures with similar interhelical angle arrangements as the obtained states. The states that are similar to experimentally obtained states are marked by a symbol corresponding to the experimental structure. Note that state 1 is similar to 5HIT only at the EF-EG angles.
(TIFF)

S2 Fig The holo N-CaM states. a) The holo N-CaM states representative structures obtained from spectral clustering. Key residues from the contact/solvent exposure analysis are highlighted in colors. b) Experimentally obtained structures with similar interhelical angle arrangements as the obtained states. The states that are similar to experimentally obtained states are marked by a symbol corresponding to the experimental structure. Note that state 4 has similar interhelical angles as 5SY1_C but a different set of inter-residue contacts.
(TIFF)

S3 Fig The apo C-CaM states. a) The apo C-CaM states representative structures obtained from spectral clustering. Key residues from the contact/solvent exposure analysis are highlighted in colors. b) Experimentally obtained structures with similar interhelical angle arrangements as the obtained states. The states that are similar to experimentally obtained states are marked by a symbol corresponding to the experimental structure.
(TIFF)

S4 Fig The apo N-CaM states. a) The apo N-CaM states representative structures obtained from spectral clustering. b) Experimentally obtained structures with similar interhelical angle arrangements as the obtained states. The states that are similar to experimentally obtained states are marked by a symbol corresponding to the experimental structure. Note that state 2 is similar to 1DMO only for the AD angle.
(TIFF)

S5 Fig Estimated free energy landscapes of the holo ensemble using data acquired from MD simulations. Note that these are not accurate estimates of free energies due to limited simulation time.
(TIFF)

S6 Fig Estimated free energy landscapes of the holo ensemble using data acquired from T-REMD simulations. Note that these are not accurate estimates of free energies due to limited simulation time.
(TIFF)

S7 Fig Estimated free energy landscapes of the holo ensemble using data acquired from REST simulations. Note that these are not accurate estimates of free energies due to limited simulation time.
(TIFF)

S8 Fig Estimated free energy landscapes of the apo ensemble using data acquired from MD simulations. Note that these are not accurate estimates of free energies due to limited simulation time.
(TIFF)

S9 Fig Estimated free energy landscapes of the apo ensemble using data acquired from T-REMD simulations. Note that these are not accurate estimates of free energies due to limited simulation time.
(TIFF)

S10 Fig Estimated free energy landscapes of the apo ensemble using data acquired from REST simulations. Note that these are not accurate estimates of free energies due to limited simulation time.
(TIFF)

S11 Fig Networks of interconversion between the states obtained in this study, as derived from the MD simulation trajectories. States that were not sampled in the plain MD simulations are disconnected from the networks. Replica exchange simulations disrupt the dynamics through coordinate exchanges. Although kinetics of toy systems can be restored from replica exchange simulations [90], application of the method to the present dataset was not possible.
(TIFF)

S12 Fig Secondary structure frequency of apo state 2 difference to average holo secondary structure frequency. The propensity of residues 129-131 to join the beta sheet and deforming the fourth Ca^2+^-loop is marked by an orange circle.
(TIFF)

S13 Fig Secondary structure frequency of holo N-CaM state 4 and 6 difference to average holo secondary structure frequency. These states overlap the apo ensemble. The beta sheet shift to residue 28/62 in N-CaM is marked by an orange ellipse.
(TIFF)

S14 Fig Holo C-CaM total relative solvent exposure for each CaM-complex structure. (EPS)

S15 Fig The residue types involved in holo C-CaM target protein contacts for each CaM-complex structure. (EPS)

S16 Fig Secondary structure frequency of holo state 6 difference to average holo secondary structure frequency. The lack of beta sheets in the C-term lobe is marked by an orange ellipse.
(TIFF)

S17 Fig Free energy landscapes over holo FH and GH interhelical angles, estimated with GMM with cross validation free energy estimator [43]. State 6 is marked with a ring, showing that it is only observed in temperature enhanced MD.
(TIFF)

S18 Fig The relative solvent exposure per CaM-complex contact residue for the different holo C-CaM states, part 1. (TIFF)

S19 Fig The relative solvent exposure per CaM-complex contact residue for the different holo C-CaM states, part 2. (TIFF)

S20 Fig The relative solvent exposure per CaM-complex contact residue for the different holo C-CaM states, part 3. (TIFF)

S21 Fig Holo N-CaM total relative solvent exposure for each CaM-complex structure. (EPS)

S22 Fig The residue types involved in holo N-CaM target protein contacts for each CaM-complex structure. (EPS)

S23 Fig The relative solvent exposure per CaM-complex contact residue for the different holo N-CaM states, part 1. (TIFF)

S24 Fig The relative solvent exposure per CaM-complex contact residue for the different holo N-CaM states, part 2. (TIFF)

S25 Fig The relative solvent exposure per CaM-complex contact residue for the different holo N-CaM states, part 3. (TIFF)

S26 Fig a) Apo C-CaM total relative solvent exposure and b) the residue types involved in apo C-CaM target protein contacts for each CaM-complex structure. (EPS)

S27 Fig The relative solvent exposure per CaM-complex contact residue for the different apo C-CaM states. (TIFF)

S28 Fig a) Apo N-CaM total relative solvent exposure and b) the residue types involved in apo N-CaM target protein contacts for each CaM-complex structure. (EPS)

S29 Fig The relative solvent exposure per CaM-complex contact residue for the different apo N-CaM states. (TIFF)

S1 Data Interhelical angles, secondary structure assignments, and data used for solvent exposure analysis of all the states in holo and apo C-CaM and N-CaM. (ZIP)

S1 Text Explanation of interhelical angles and comparison of sampled states and experimental structures. A discussion of derived rate constants is also provided.
(PDF)

## References

[1] Stevens FC. Calmodulin: an introduction. Canadian Journal of Biochemistry and Cell Biology. 1983;61(8):906–910. doi: 10.1139/o83-115 6313166 10.1139/o83-115

[2] Kuboniwa H, Tjandra N, Grzesiek S, Ren H, Klee CB, Bax A. Solution structure of calcium-free calmodulin. Nature Structural & Molecular Biology. 1995;2(9):768–776. doi: 10.1038/nsb0995-768 10.1038/nsb0995-768 7552748

[3] Kanehisa M, Furumichi M, Tanabe M, Sato Y, Morishima K. KEGG: new perspectives on genomes, pathways, diseases and drugs. Nucleic Acids Res. 2017;45(D1):D353–D361. doi: 10.1093/nar/gkw1092 27899662 10.1093/nar/gkw1092 PMC5210567

[4] Smith DMA, Straatsma TP, Squier TC. Retention of Conformational Entropy upon Calmodulin Binding to Target Peptides Is Driven by Transient Salt Bridges. Biophysical Journal. 2012;103(7):1576–1584. doi: 10.1016/j.bpj.2012.08.037 23062350 10.1016/j.bpj.2012.08.037 PMC3471477

[5] Rhoads AR, Friedberg F. Sequence motifs for calmodulin recognition. FASEB journal: official publication of the Federation of American Societies for Experimental Biology. 1997;11(5):331–340. doi: 10.1096/fasebj.11.5.9141499 9141499 10.1096/fasebj.11.5.9141499

[6] Bähler M, Rhoads A. Calmodulin signaling via the IQ motif. FEBS Letters. 2002;513(1):107–113. doi: 10.1016/S0014-5793(01)03239-2 11911888 10.1016/s0014-5793(01)03239-2

[7] Tidow H, Nissen P. Structural diversity of calmodulin binding to its target sites. FEBS Journal. 2013;280(21):5551–5565. doi: 10.1111/febs.12296 23601118 10.1111/febs.12296

[8] Feldkamp MD, Yu L, Shea MA. Structural and Energetic Determinants of Apo Calmodulin Binding to the IQ Motif of the NaV1.2 Voltage-Dependent Sodium Channel. Structure. 2011;19(5):733–747. doi: 10.1016/j.str.2011.02.009 21439835 10.1016/j.str.2011.02.009 PMC3094505

[9] Mahling R, Kilpatrick AM, Shea MA. Backbone resonance assignments of complexes of human voltage-dependent sodium channel NaV1.2 IQ motif peptide bound to apo calmodulin and to the C-domain fragment of apo calmodulin. Biomolecular NMR Assignments. 2017; p. 1–7. 10.1007/s12104-017-9767-2 PMC5791537 28823028

[10] Strulovich R, Tobelaim WS, Attali B, Hirsch JA. Structural Insights into the M-Channel Proximal C-Terminus/Calmodulin Complex. Biochemistry. 2016;55(38):5353–5365. doi: 10.1021/acs.biochem.6b00477 27564677 10.1021/acs.biochem.6b00477

[11] Keller JP. Solution of the structure of a calmodulin–peptide complex in a novel configuration from a variably twinned data set. Acta Crystallographica Section D: Structural Biology. 2017;73(1):22–31. doi: 10.1107/S2059798316019318 28045382 10.1107/S2059798316019318

[12] Marques-Carvalho MJ, Oppermann J, Muñoz E, Fernandes AS, Gabant G, Cadene M, et al Molecular Insights into the Mechanism of Calmodulin Inhibition of the EAG1 Potassium Channel. Structure. 2016;24(10):1742–1754. doi: 10.1016/j.str.2016.07.020 27618660 10.1016/j.str.2016.07.020 PMC5176025

[13] Lee K, Alphonse S, Piserchio A, Tavares CDJ, Giles DH, Wellmann RM, et al Structural Basis for the Recognition of Eukaryotic Elongation Factor 2 Kinase by Calmodulin. Structure. 2016;24(9):1441–1451. doi: 10.1016/j.str.2016.06.015 27499441 10.1016/j.str.2016.06.015 PMC5014583

[14] Schumacher MA, Rivard AF, Bächinger HP, Adelman JP. Structure of the gating domain of a Ca2+-activated K+ channel complexed with Ca2+/calmodulin. Nature. 2001;410(6832):1120–1124. doi: 10.1038/35074145 11323678 10.1038/35074145

[15] Li J, Chen Y, Deng Y, Unarta IC, Lu Q, Huang X, et al Ca2+-Induced Rigidity Change of the Myosin VIIa IQ Motif-Single alpha Helix Lever Arm Extension. Structure. 2017;25(4):579–591.e4. doi: 10.1016/j.str.2017.02.002 28262393 10.1016/j.str.2017.02.002

[16] Zhang Y, Matt L, Patriarchi T, Malik ZA, Chowdhury D, Park DK, et al Capping of the N-terminus of PSD-95 by calmodulin triggers its postsynaptic release. The EMBO Journal. 2014;33(12):1341–1353. doi: 10.1002/embj.201488126 24705785 10.1002/embj.201488126 PMC4194123

[17] Wang C, Chung BC, Yan H, Wang HG, Lee SY, Pitt GS. Structural analyses of Ca^2+^/CaM interaction with Na_V_ channel C-termini reveal mechanisms of calcium-dependent regulation. Nature Communications. 2014;5:ncomms5896. 10.1038/ncomms5896 PMC4170523 25232683

[18] Dunlap TB, Guo HF, Cook EC, Holbrook E, Rumi-Masante J, Lester TE, et al Stoichiometry of the Calcineurin Regulatory Domain–Calmodulin Complex. Biochemistry. 2014;53(36):5779–5790. doi: 10.1021/bi5004734 25144868 10.1021/bi5004734

[19] Houdusse A, Gaucher JF, Krementsova E, Mui S, Trybus KM, Cohen C. Crystal structure of apo-calmodulin bound to the first two IQ motifs of myosin V reveals essential recognition features. Proceedings of the National Academy of Sciences. 2006;103(51):19326–19331. doi: 10.1073/pnas.0609436103 10.1073/pnas.0609436103 PMC1687203 17151196

[20] Lu Q, Li J, Ye F, Zhang M. Structure of myosin-1c tail bound to calmodulin provides insights into calcium-mediated conformational coupling. Nature Structural & Molecular Biology. 2015;22(1):81–88. doi: 10.1038/nsmb.2923 10.1038/nsmb.2923 25437912

[21] Chichili VPR, Xiao Y, Seetharaman J, Cummins TR, Sivaraman J. Structural Basis for the Modulation of the Neuronal Voltage-Gated Sodium Channel Na_V_1.6 by Calmodulin. Scientific Reports. 2013;3:srep02435 doi: 10.1038/srep02435 10.1038/srep02435 PMC3743062 23942337

[22] Chen LT, Liang WX, Chen S, Li RK, Tan JL, Xu PF, et al Functional and molecular features of the calmodulin-interacting protein IQCG required for haematopoiesis in zebrafish. Nature Communications. 2014;5:ncomms4811. 10.1038/ncomms4811 24787902

[23] Hoang J, Prosser RS. Conformational Selection and Functional Dynamics of Calmodulin: A 19F Nuclear Magnetic Resonance Study. Biochemistry. 2014;53(36):5727–5736. doi: 10.1021/bi500679c 25148136 10.1021/bi500679c

[24] Zhang M, Tanaka T, Ikura M. Calcium-induced conformational transition revealed by the solution structure of apo calmodulin. Nature Structural & Molecular Biology. 1995;2(9):758–767. doi: 10.1038/nsb0995-758 10.1038/nsb0995-758 7552747

[25] Zhang M, Abrams C, Wang L, Gizzi A, He L, Lin R, et al Structural Basis for Calmodulin as a Dynamic Calcium Sensor. Structure. 2012;20(5):911–923. doi: 10.1016/j.str.2012.03.019 22579256 10.1016/j.str.2012.03.019 PMC3372094

[26] Wriggers W, Mehler E, Pitici F, Weinstein H, Schulten K. Structure and Dynamics of Calmodulin in Solution. Biophysical Journal. 1998;74(4):1622–1639. doi: 10.1016/S0006-3495(98)77876-2 9545028 10.1016/S0006-3495(98)77876-2 PMC1299510

[27] Shepherd CM, Vogel HJ. A Molecular Dynamics Study of Ca2+-Calmodulin: Evidence of Interdomain Coupling and Structural Collapse on the Nanosecond Timescale. Biophysical Journal. 2004;87(2):780–791. doi: 10.1529/biophysj.103.033266 15298887 10.1529/biophysj.103.033266 PMC1304488

[28] Fiorin G, Biekofsky RR, Pastore A, Carloni P. Unwinding the helical linker of calcium-loaded calmodulin: A molecular dynamics study. Proteins: Structure, Function, and Bioinformatics. 2005;61(4):829–839. doi: 10.1002/prot.20597 10.1002/prot.20597 16193483

[29] Project E, Friedman R, Nachliel E, Gutman M. A Molecular Dynamics Study of the Effect of Ca2+ Removal on Calmodulin Structure. Biophysical Journal. 2006;90(11):3842–3850. doi: 10.1529/biophysj.105.077792 16533845 10.1529/biophysj.105.077792 PMC1459500

[30] Fiorin G, Pastore A, Carloni P, Parrinello M. Using Metadynamics to Understand the Mechanism of Calmodulin/Target Recognition at Atomic Detail. Biophysical Journal. 2006;91(8):2768–2777. doi: 10.1529/biophysj.106.086611 16877506 10.1529/biophysj.106.086611 PMC1578468

[31] Shukla D, Peck A, Pande VS. Conformational heterogeneity of the calmodulin binding interface. Nature Communications. 2016;7:10910 doi: 10.1038/ncomms10910 27040077 10.1038/ncomms10910 PMC4822001

[32] Ma B, Kumar S, Tsai CJ, Nussinov R. Folding funnels and binding mechanisms. Protein Engineering, Design and Selection. 1999;12(9):713–720. doi: 10.1093/protein/12.9.713 10.1093/protein/12.9.713 10506280

[33] Koshland DE. The Key–Lock Theory and the Induced Fit Theory. Angewandte Chemie International Edition in English. 1995;33(23-24):2375–2378. doi: 10.1002/anie.199423751

[34] Pabon NA, Camacho CJ. Probing protein flexibility reveals a mechanism for selective promiscuity. eLife. 2017;6:e22889 doi: 10.7554/eLife.22889 28432789 10.7554/eLife.22889 PMC5446241

[35] Babu YS, Bugg CE, Cook WJ. Structure of calmodulin refined at 2.2 A resolution. Journal of Molecular Biology. 1988;204(1):191–204. doi: 10.1016/0022-2836(88)90608-0 3145979 10.1016/0022-2836(88)90608-0

[36] Ishida H, Nakashima Ki, Kumaki Y, Nakata M, Hikichi K, Yazawa M. The solution structure of apocalmodulin from Saccharomyces cerevisiae implies a mechanism for its unique Ca2+ binding property. Biochemistry. 2002;41(52):15536–15542. doi: 10.1021/bi020330r 12501182 10.1021/bi020330r

[37] Jo S, Kim T, Iyer VG, Im W. CHARMM-GUI: A web-based graphical user interface for CHARMM. Journal of computational chemistry. 2008;29(11):1859–1865. doi: 10.1002/jcc.20945 18351591 10.1002/jcc.20945

[38] Lee J, Cheng X, Swails JM, Yeom MS, Eastman PK, Lemkul JA, et al CHARMM-GUI input generator for NAMD, GROMACS, AMBER, OpenMM, and CHARMM/OpenMM simulations using the CHARMM36 additive force field. Journal of chemical theory and computation. 2015;12(1):405–413. doi: 10.1021/acs.jctc.5b00935 26631602 10.1021/acs.jctc.5b00935 PMC4712441

[39] MacKerell AD, Bashford D, Bellott M, Dunbrack RL, Evanseck JD, Field MJ, et al All-atom empirical potential for molecular modeling and dynamics studies of proteins. The Journal of Physical Chemistry B. 1998;102(18):3586–3616. doi: 10.1021/jp973084f 24889800 10.1021/jp973084f

[40] Jorgensen WL, Chandrasekhar J, Madura JD, Impey RW, Klein ML. Comparison of simple potential functions for simulating liquid water. The Journal of Chemical Physics. 1983;79(2):926–935. doi: 10.1063/1.445869

[41] Liao J, Marinelli F, Lee C, Huang Y, Faraldo-Gómez JD, Jiang Y. Mechanism of extracellular ion exchange and binding-site occlusion in a sodium/calcium exchanger. Nature Structural & Molecular Biology. 2016;23(6):590–599. doi: 10.1038/nsmb.3230 10.1038/nsmb.3230 PMC4918766 27183196

[42] Berendsen HJC, Postma JPM, van Gunsteren WF, DiNola A, Haak JR. Molecular dynamics with coupling to an external bath. The Journal of Chemical Physics. 1984;81(8):3684–3690. doi: 10.1063/1.448118

[43] Westerlund AM, Harpole TJ, Blau C, Delemotte L. Inference of Calmodulin’s Ca2+-Dependent Free Energy Landscapes via Gaussian Mixture Model Validation. Journal of Chemical Theory and Computation. 2017 29144736 10.1021/acs.jctc.7b00346

[44] Darden T, York D, Pedersen L. Particle mesh Ewald: An N?log(N) method for Ewald sums in large systems. The Journal of Chemical Physics. 1993;98(12):10089–10092. doi: 10.1063/1.464397

[45] Nosé S. A unified formulation of the constant temperature molecular dynamics methods. The Journal of Chemical Physics. 1984;81(1):511–519. doi: 10.1063/1.447334

[46] Parrinello M, Rahman A. Polymorphic transitions in single crystals: A new molecular dynamics method. Journal of Applied Physics. 1981;52(12):7182–7190. doi: 10.1063/1.328693

[47] Hess B, Bekker H, Berendsen HJC, Fraaije JGEM. LINCS: A linear constraint solver for molecular simulations. Journal of Computational Chemistry. 1997;18(12):1463–1472. doi: 10.1002/(SICI)1096-987X(199709)18:12<1463::AID-JCC4>3.0.CO;2-H

[48] Sugita Y, Okamoto Y. Replica-exchange molecular dynamics method for protein folding. Chemical Physics Letters. 1999;314(1–2):141–151. doi: 10.1016/S0009-2614(99)01123-9

[49] Liu P, Kim B, Friesner RA, Berne BJ. Replica exchange with solute tempering: A method for sampling biological systems in explicit water. Proceedings of the National Academy of Sciences of the United States of America. 2005;102(39):13749–13754. doi: 10.1073/pnas.0506346102 16172406 10.1073/pnas.0506346102 PMC1236566

[50] Wang L, Friesner RA, Berne BJ. Replica Exchange with Solute Scaling: A More Efficient Version of Replica Exchange with Solute Tempering (REST2). The Journal of Physical Chemistry B. 2011;115(30):9431–9438. doi: 10.1021/jp204407d 21714551 10.1021/jp204407d PMC3172817

[51] Huang X, Hagen M, Kim B, Friesner RA, Zhou R, Berne BJ. Replica Exchange with Solute Tempering:? Efficiency in Large Scale Systems. The Journal of Physical Chemistry B. 2007;111(19):5405–5410. doi: 10.1021/jp068826w 17439169 10.1021/jp068826w PMC2744475

[52] Rhee YM, Pande VS. Multiplexed-Replica Exchange Molecular Dynamics Method for Protein Folding Simulation. Biophysical Journal. 2003;84(2):775–786. doi: 10.1016/S0006-3495(03)74897-8 12547762 10.1016/S0006-3495(03)74897-8 PMC1302658

[53] Patriksson A, vd Spoel D. A temperature predictor for parallel tempering simulations. Physical Chemistry Chemical Physics. 2008;10(15):2073–2077. doi: 10.1039/b716554d 18688361 10.1039/b716554d

[54] Tribello GA, Bonomi M, Branduardi D, Camilloni C, Bussi G. PLUMED 2: New feathers for an old bird. Computer Physics Communications. 2014;185(2):604–613. doi: 10.1016/j.cpc.2013.09.018

[55] Abraham MJ, Murtola T, Schulz R, Páll S, Smith JC, Hess B, et al GROMACS: High performance molecular simulations through multi-level parallelism from laptops to supercomputers. SoftwareX. 2015;1–2:19–25. doi: 10.1016/j.softx.2015.06.001

[56] Bussi G. Hamiltonian replica exchange in GROMACS: a flexible implementation. Molecular Physics. 2014;112(3-4):379–384. doi: 10.1080/00268976.2013.824126

[57] Ponzoni L, Polles G, Carnevale V, Micheletti C. SPECTRUS: A Dimensionality Reduction Approach for Identifying Dynamical Domains in Protein Complexes from Limited Structural Datasets. Structure. 2015;23(8):1516–1525. doi: 10.1016/j.str.2015.05.022 26165596 10.1016/j.str.2015.05.022

[58] Ng AY, Jordan MI, Weiss Y. On Spectral Clustering: Analysis and an Algorithm. In: Proceedings of the 14th International Conference on Neural Information Processing Systems: Natural and Synthetic. NIPS’01. Cambridge, MA, USA: MIT Press; 2001. p. 849–856. Available from: http://dl.acm.org/citation.cfm?id=2980539.2980649.

[59] Bellman R. Dynamic Programming. 1st ed Princeton, NJ, USA: Princeton University Press; 1957.

[60] Gsponer J, Christodoulou J, Cavalli A, Bui JM, Richter B, Dobson CM, et al A Coupled Equilibrium Shift Mechanism in Calmodulin-Mediated Signal Transduction. Structure. 2008;16(5):736–746. doi: 10.1016/j.str.2008.02.017 18462678 10.1016/j.str.2008.02.017 PMC2428103

[61] Rao ST, Wu S, Satyshur KA, Sundaralingam M, Ling KY, Kung C. Structure of Paramecium tetraurelia calmodulin at 1.8 Å resolution. Protein Science. 1993;2(3):436–447. doi: 10.1002/pro.5560020316 8453381 10.1002/pro.5560020316 PMC2142389

[62] Chattopadhyaya R, Meador WE, Means AR, Quiocho FA. Calmodulin structure refined at 1.7 Å resolution. Journal of Molecular Biology. 1992;228(4):1177–1192. doi: 10.1016/0022-2836(92)90324-D 1474585 10.1016/0022-2836(92)90324-d

[63] Wilson MA, Brunger AT. The 1.0 Å crystal structure of Ca2+-bound calmodulin: an analysis of disorder and implications for functionally relevant plasticity. Edited by Wilson I. Journal of Molecular Biology. 2000;301(5):1237–1256. doi: 10.1006/jmbi.2000.4029 10966818 10.1006/jmbi.2000.4029

[64] Symersky J, Lin G, Li S, Qiu S, Carson M, Schormann N, et al Structural genomics of caenorhabditis elegans: Crystal structure of calmodulin. Proteins: Structure, Function, and Bioinformatics. 2003;53(4):947–949. doi: 10.1002/prot.10482 10.1002/prot.10517 14635136

[65] Ban C, Ramakrishnan B, Ling KY, Kung C, Sundaralingam M. Structure of the recombinant Paramecium tetraurelia calmodulin at 1.68 Å resolution. Acta Crystallographica Section D: Biological Crystallography. 1994;50(1):50–63. doi: 10.1107/S0907444993007991 15299476 10.1107/S0907444993007991

[66] Fallon JL, Quiocho FA. A Closed Compact Structure of Native Ca2+-Calmodulin. Structure. 2003;11(10):1303–1307. doi: 10.1016/j.str.2003.09.004 14527397 10.1016/j.str.2003.09.004

[67] Rupp B, Marshak DR, Parkin S. Crystallization and preliminary X-ray analysis of two new crystal forms of calmodulin. Acta Crystallographica Section D: Biological Crystallography. 1996;52(2):411–413. doi: 10.1107/S0907444995011826 15299715 10.1107/S0907444995011826

[68] Taylor DA, Sack JS, Maune JF, Beckingham K, Quiocho FA. Structure of a recombinant calmodulin from Drosophila melanogaster refined at 2.2-A resolution. The Journal of Biological Chemistry. 1991;266(32):21375–21380. 1939171 10.2210/pdb4cln/pdb

[69] Kursula P. Crystallographic snapshots of initial steps in the collapse of the calmodulin central helix. Acta Crystallographica Section D: Biological Crystallography. 2014;70(1):24–30. doi: 10.1107/S1399004713024437 24419375 10.1107/S1399004713024437

[70] O’Donnell SE, Newman RA, Witt TJ, Hultman R, Froehlig JR, Christensen AP, et al Chapter 21 Thermodynamics and Conformational Change Governing Domain–Domain Interactions of Calmodulin. Methods in Enzymology. 2009;466:503–526. doi: 10.1016/S0076-6879(09)66021-3 21609874 10.1016/S0076-6879(09)66021-3

[71] Bentrop D, Bertini I, Cremonini MA, Forsén S, Luchinat C, Malmendal A. Solution Structure of the Paramagnetic Complex of the N-Terminal Domain of Calmodulin with Two Ce3+ Ions by 1H NMR. Biochemistry. 1997;36(39):11605–11618. doi: 10.1021/bi971022+ 9305950 10.1021/bi971022+

[72] Kabsch W, Sander C. Dictionary of protein secondary structure: pattern recognition of hydrogen-bonded and geometrical features. Biopolymers. 1983;22(12):2577–2637. doi: 10.1002/bip.360221211 6667333 10.1002/bip.360221211

[73] McGibbon RT, Beauchamp KA, Harrigan MP, Klein C, Swails JM, Hernández CX, et al MDTraj: A Modern Open Library for the Analysis of Molecular Dynamics Trajectories. Biophysical Journal. 2015;109(8):1528–1532. doi: 10.1016/j.bpj.2015.08.015 26488642 10.1016/j.bpj.2015.08.015 PMC4623899

[74] Humphrey W, Dalke A, Schulten K. VMD—Visual Molecular Dynamics. Journal of Molecular Graphics. 1996;14:33–38. doi: 10.1016/0263-7855(96)00018-5 8744570 10.1016/0263-7855(96)00018-5

[75] Yamada Y, Matsuo T, Iwamoto H, Yagi N. A Compact Intermediate State of Calmodulin in the Process of Target Binding. Biochemistry. 2012;51(19):3963–3970. doi: 10.1021/bi3002192 22548417 10.1021/bi3002192

[76] Chen YG, Hummer G. Slow Conformational Dynamics and Unfolding of the Calmodulin C-Terminal Domain. Journal of the American Chemical Society. 2007;129(9):2414–2415. doi: 10.1021/ja067791a 17290995 10.1021/ja067791a

[77] Hines KE, Middendorf TR, Aldrich RW. Determination of parameter identifiability in nonlinear biophysical models: A Bayesian approach. The Journal of General Physiology. 2014;143(3):401–416. doi: 10.1085/jgp.201311116 24516188 10.1085/jgp.201311116 PMC3933937

[78] Halling DB, Liebeskind BJ, Hall AW, Aldrich RW. Conserved properties of individual Ca2+-binding sites in calmodulin. Proceedings of the National Academy of Sciences. 2016;113(9):E1216–E1225. doi: 10.1073/pnas.1600385113 10.1073/pnas.1600385113 PMC4780646 26884197

[79] Aykut AO, Atilgan AR, Atilgan C. Designing Molecular Dynamics Simulations to Shift Populations of the Conformational States of Calmodulin. PLOS Computational Biology. 2013;9(12):e1003366 doi: 10.1371/journal.pcbi.1003366 24339763 10.1371/journal.pcbi.1003366 PMC3854495

[80] Marsman RF, Barc J, Beekman L, Alders M, Dooijes D, van den Wijngaard A, et al A Mutation in CALM1 Encoding Calmodulin in Familial Idiopathic Ventricular Fibrillation in Childhood and Adolescence. Journal of the American College of Cardiology. 2014;63(3):259–266. doi: 10.1016/j.jacc.2013.07.091 24076290 10.1016/j.jacc.2013.07.091

[81] Limpitikul WB, Dick IE, Joshi-Mukherjee R, Overgaard MT, George AL, Yue DT. Calmodulin mutations associated with long QT syndrome prevent inactivation of cardiac L-type Ca2+ currents and promote proarrhythmic behavior in ventricular myocytes. Journal of Molecular and Cellular Cardiology. 2014;74(Supplement C):115–124. doi: 10.1016/j.yjmcc.2014.04.022 24816216 10.1016/j.yjmcc.2014.04.022 PMC4262253

[82] Rocchetti M, Sala L, Dreizehnter L, Crotti L, Sinnecker D, Mura M, et al Elucidating arrhythmogenic mechanisms of long-QT syndrome CALM1-F142L mutation in patient-specific induced pluripotent stem cell-derived cardiomyocytes. Cardiovascular Research. 2017;113(5):531–541. doi: 10.1093/cvr/cvx006 28158429 10.1093/cvr/cvx006

[83] Crotti L, Johnson CN, Graf E, Ferrari GMD, Cuneo BF, Ovadia M, et al Calmodulin Mutations Associated With Recurrent Cardiac Arrest in InfantsClinical Perspective. Circulation. 2013;127(9):1009–1017. doi: 10.1161/CIRCULATIONAHA.112.001216 23388215 10.1161/CIRCULATIONAHA.112.001216 PMC3834768

[84] Okazaki Ki, Takada S. Dynamic energy landscape view of coupled binding and protein conformational change: Induced-fit versus population-shift mechanisms. Proceedings of the National Academy of Sciences. 2008;105(32):11182–11187. doi: 10.1073/pnas.0802524105 10.1073/pnas.0802524105 PMC2516237 18678900

[85] Chen Y, Clarke OB, Kim J, Stowe S, Kim YK, Assur Z, et al Structure of the STRA6 receptor for retinol uptake. Science. 2016;353(6302):aad8266 doi: 10.1126/science.aad8266 27563101 10.1126/science.aad8266 PMC5114850

[86] Whicher JR, MacKinnon R. Structure of the voltage-gated K+ channel Eag1 reveals an alternative voltage sensing mechanism. Science. 2016;353(6300):664–669. doi: 10.1126/science.aaf8070 27516594 10.1126/science.aaf8070 PMC5477842

[87] Irene D, Huang JW, Chung TY, Li FY, Tzen JTC, Lin TH, et al Binding orientation and specificity of calmodulin to rat olfactory cyclic nucleotide-gated ion channel. Journal of Biomolecular Structure and Dynamics. 2013;31(4):414–425. doi: 10.1080/07391102.2012.703069 22877078 10.1080/07391102.2012.703069

[88] Tidow H, Poulsen LR, Andreeva A, Knudsen M, Hein KL, Wiuf C, et al A bimodular mechanism of calcium control in eukaryotes. Nature. 2012;491(7424):468–472. doi: 10.1038/nature11539 23086147 10.1038/nature11539

[89] Chagot B, Chazin WJ. Solution NMR Structure of Apo-Calmodulin in Complex with the IQ Motif of Human Cardiac Sodium Channel NaV1.5. Journal of Molecular Biology. 2011;406(1):106–119. doi: 10.1016/j.jmb.2010.11.046 21167176 10.1016/j.jmb.2010.11.046 PMC3030672

[90] Stelzl LS, Hummer G. Kinetics from Replica Exchange Molecular Dynamics Simulations. Journal of Chemical Theory and Computation. 2017;13(8):3927–3935. doi: 10.1021/acs.jctc.7b00372 28657736 10.1021/acs.jctc.7b00372

