# Supplementary figures and images for: Effect of Ca2+ on the promiscuous target-protein binding of calmodulin
Source: PLoS Comput Biol. 2018 Apr 3;14(4):e1006072. doi: 10.1371/journal.pcbi.1006072 (PMC5898786; doi:10.1371/journal.pcbi.1006072)

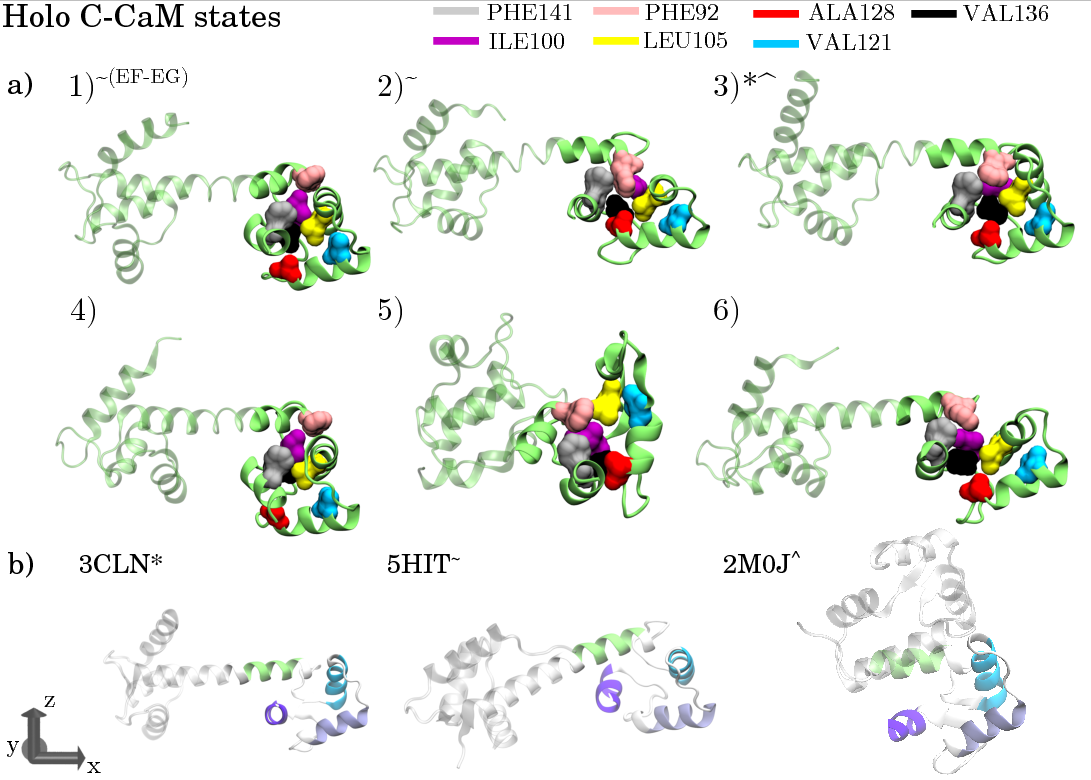

Supplement: S1 Fig — a) The holo C-CaM states representative structures obtained from spectral clustering. Key residues from the contact/solvent exposure analysis are highlighted in colors. b) Experimentally obtained structures with similar interhelical angle arrangements as the obtained states. The states that are similar to experimentally obtained states are marked by a symbol corresponding to the experimental structure. Note that state 1 is similar to 5HIT only at the EF-EG angles. (TIFF) [file pcbi.1006072.s001.tiff]

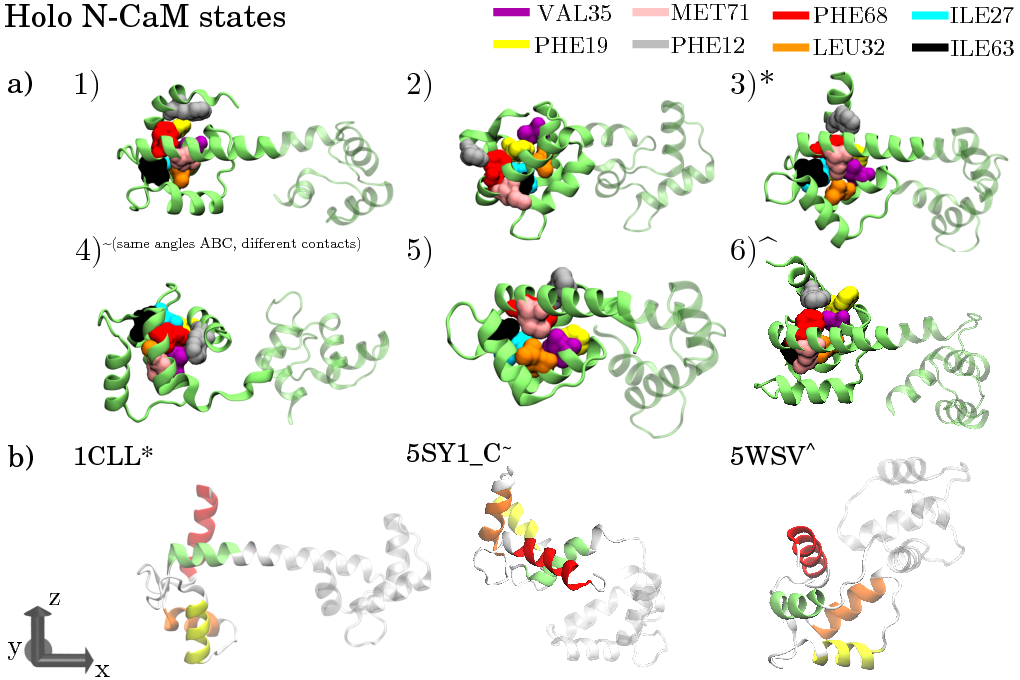

Supplement: S2 Fig — a) The holo N-CaM states representative structures obtained from spectral clustering. Key residues from the contact/solvent exposure analysis are highlighted in colors. b) Experimentally obtained structures with similar interhelical angle arrangements as the obtained states. The states that are similar to experimentally obtained states are marked by a symbol corresponding to the experimental structure. Note that state 4 has similar interhelical angles as 5SY1_C but a different set of inter-residue contacts. (TIFF) [file pcbi.1006072.s002.tiff]

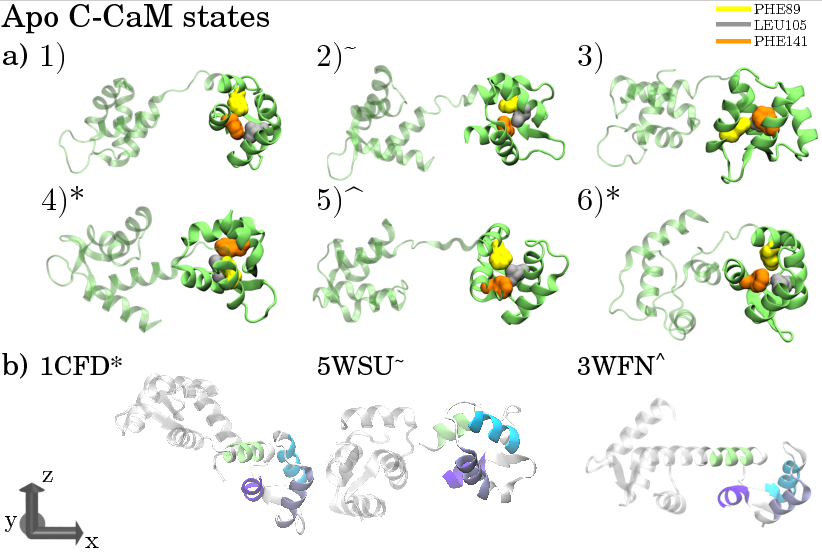

Supplement: S3 Fig — a) The apo C-CaM states representative structures obtained from spectral clustering. Key residues from the contact/solvent exposure analysis are highlighted in colors. b) Experimentally obtained structures with similar interhelical angle arrangements as the obtained states. The states that are similar to experimentally obtained states are marked by a symbol corresponding to the experimental structure. (TIFF) [file pcbi.1006072.s003.tiff]

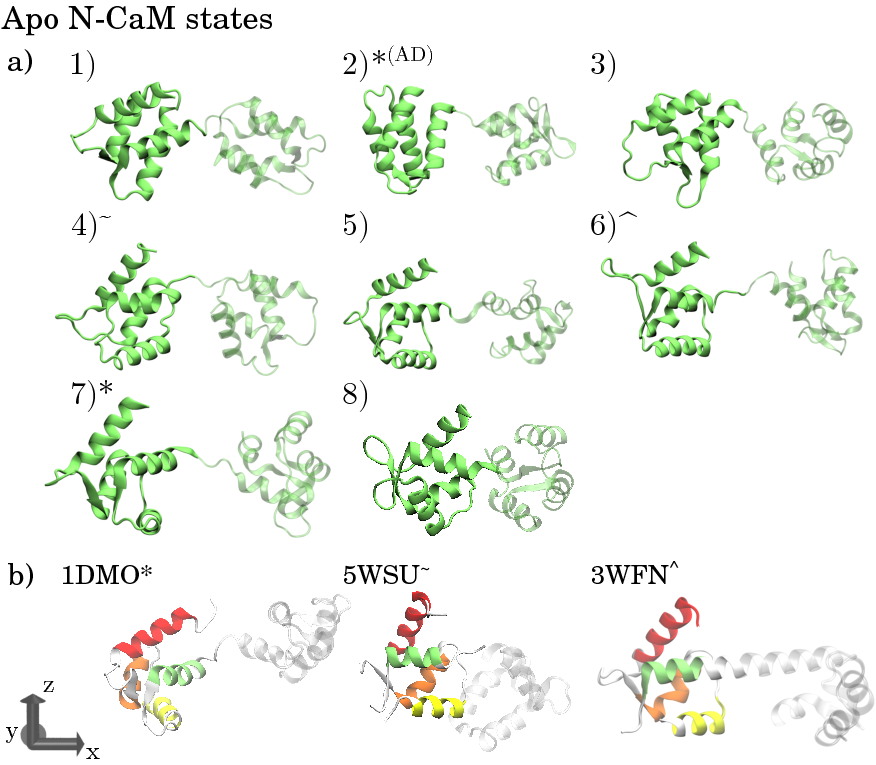

Supplement: S4 Fig — a) The apo N-CaM states representative structures obtained from spectral clustering. b) Experimentally obtained structures with similar interhelical angle arrangements as the obtained states. The states that are similar to experimentally obtained states are marked by a symbol corresponding to the experimental structure. Note that state 2 is similar to 1DMO only for the AD angle. (TIFF) [file pcbi.1006072.s004.tiff]

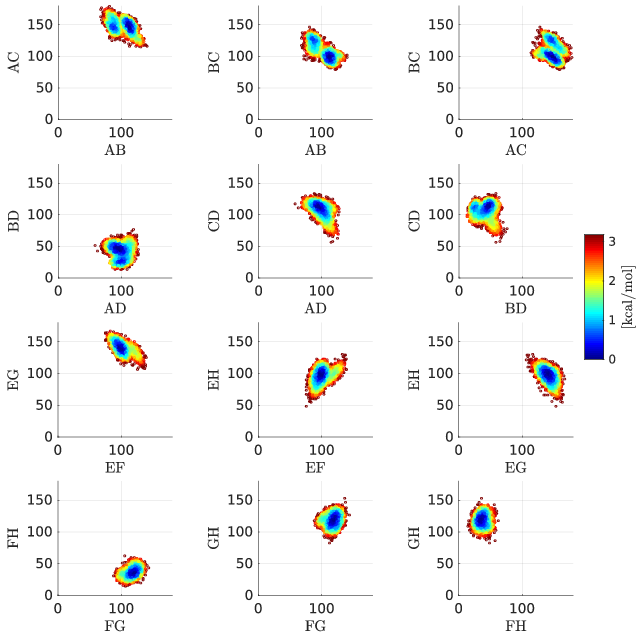

Supplement: S5 Fig — Note that these are not accurate estimates of free energies due to limited simulation time. (TIFF) [file pcbi.1006072.s005.tiff]

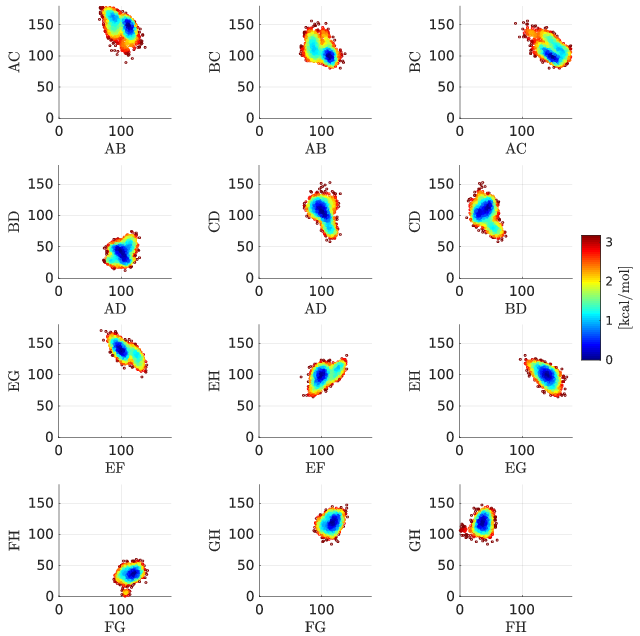

Supplement: S6 Fig — Note that these are not accurate estimates of free energies due to limited simulation time. (TIFF) [file pcbi.1006072.s006.tiff]

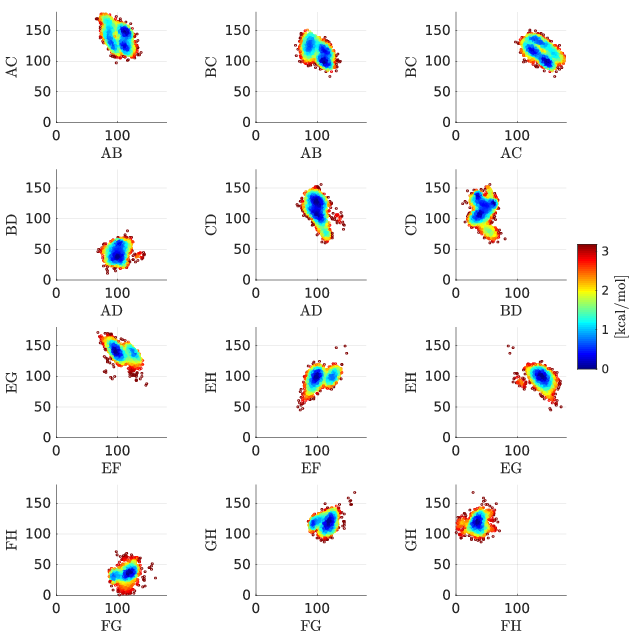

Supplement: S7 Fig — Note that these are not accurate estimates of free energies due to limited simulation time. (TIFF) [file pcbi.1006072.s007.tiff]

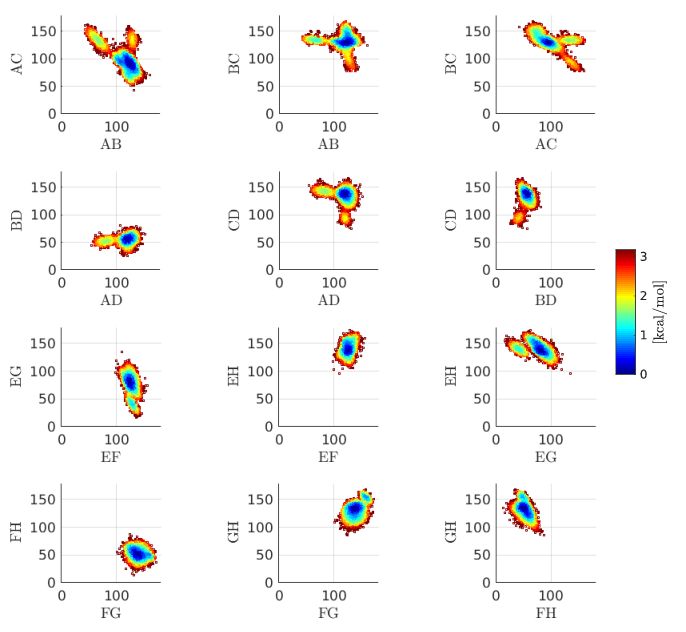

Supplement: S8 Fig — Note that these are not accurate estimates of free energies due to limited simulation time. (TIFF) [file pcbi.1006072.s008.tiff]

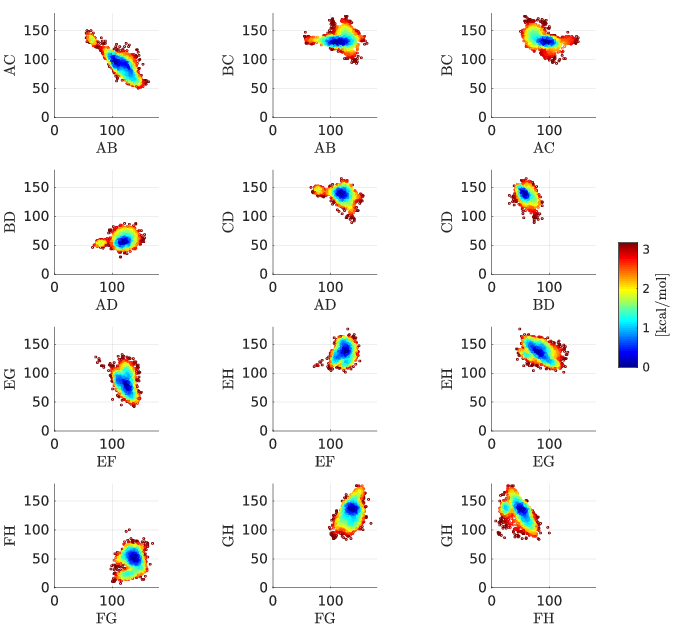

Supplement: S9 Fig — Note that these are not accurate estimates of free energies due to limited simulation time. (TIFF) [file pcbi.1006072.s009.tiff]

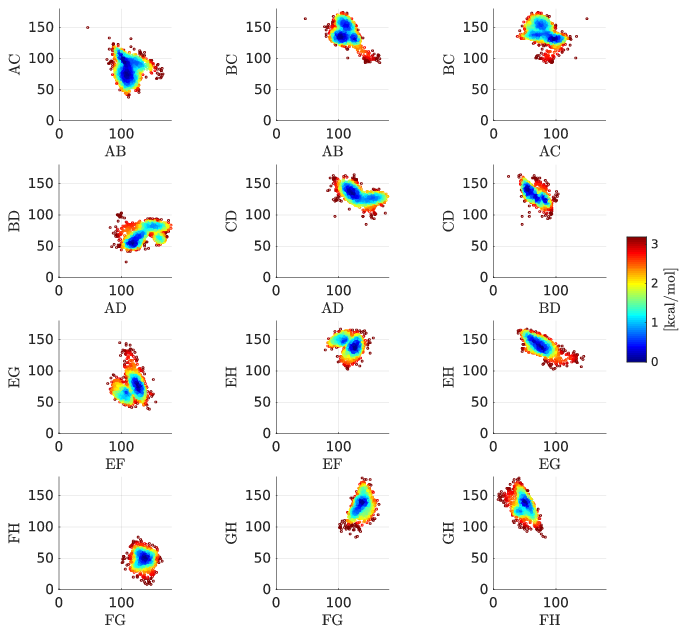

Supplement: S10 Fig — Note that these are not accurate estimates of free energies due to limited simulation time. (TIFF) [file pcbi.1006072.s010.tiff]

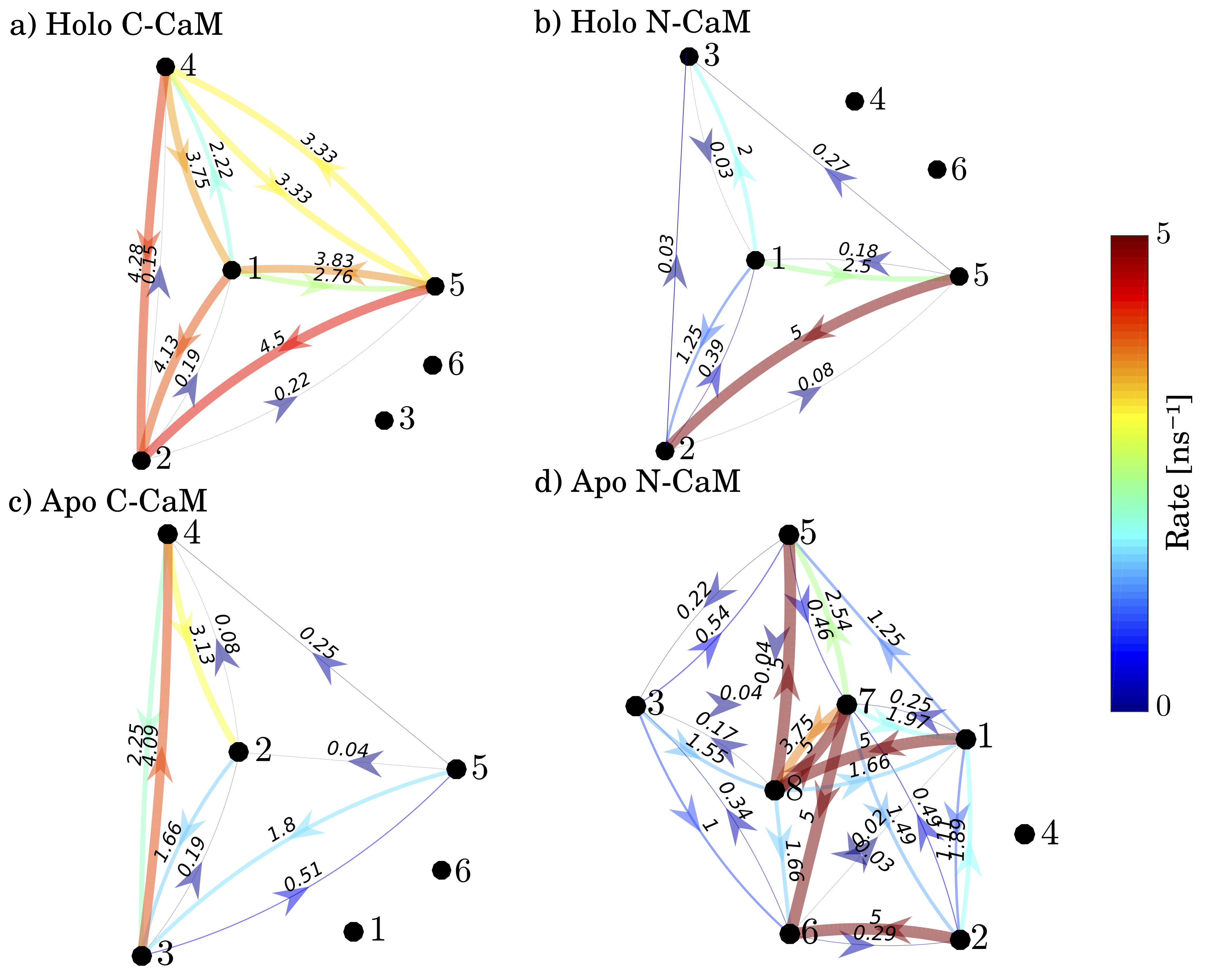

Supplement: S11 Fig — States that were not sampled in the plain MD simulations are disconnected from the networks. Replica exchange simulations disrupt the dynamics through coordinate exchanges. Although kinetics of toy systems can be restored from replica exchange simulations [90], application of the method to the present dataset was not possible. (TIFF) [file pcbi.1006072.s011.tiff]

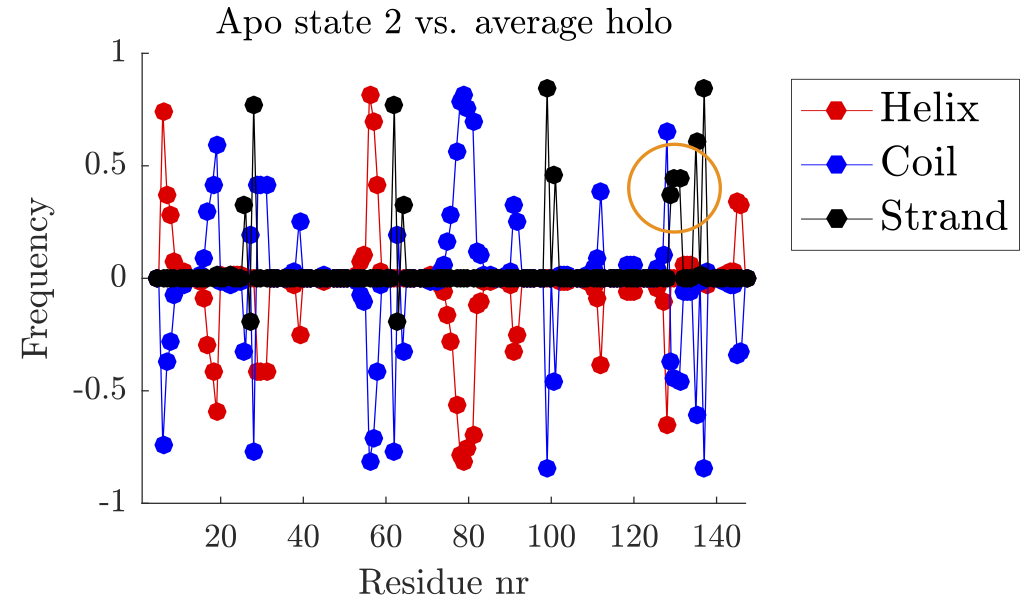

Supplement: S12 Fig — The propensity of residues 129-131 to join the beta sheet and deforming the fourth Ca2+-loop is marked by an orange circle. (TIFF) [file pcbi.1006072.s012.tiff]

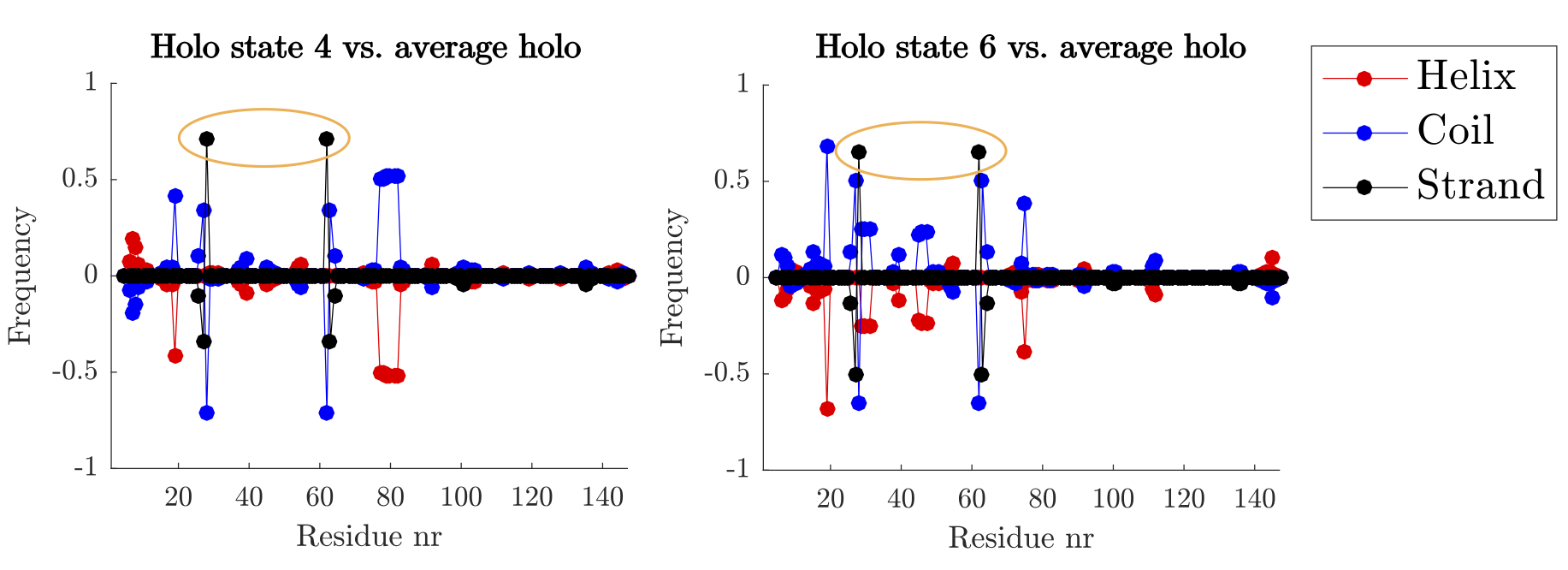

Supplement: S13 Fig — These states overlap the apo ensemble. The beta sheet shift to residue 28/62 in N-CaM is marked by an orange ellipse. (TIFF) [file pcbi.1006072.s013.tiff]

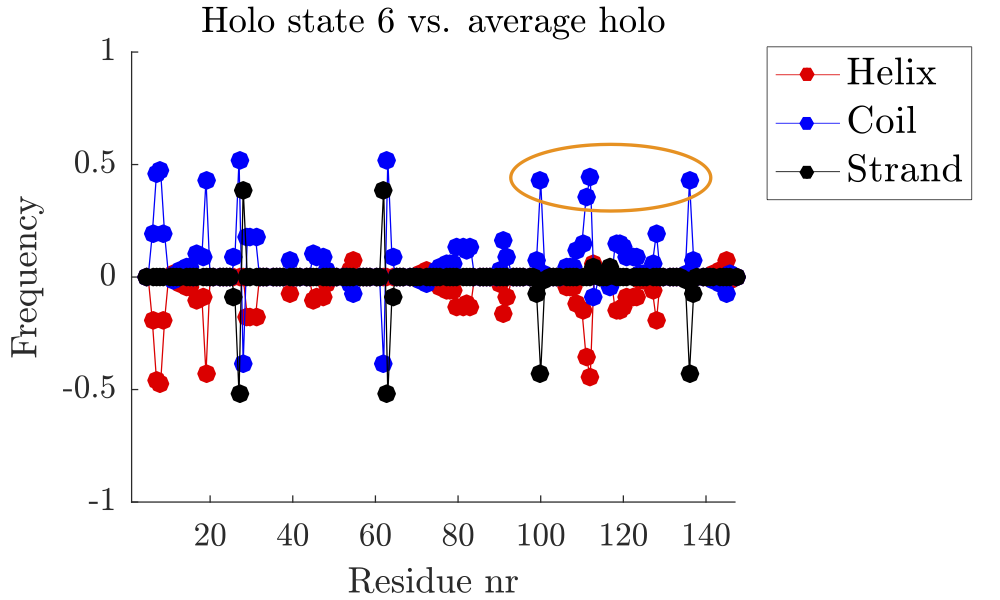

Supplement: S16 Fig — The lack of beta sheets in the C-term lobe is marked by an orange ellipse. (TIFF) [file pcbi.1006072.s016.tiff]

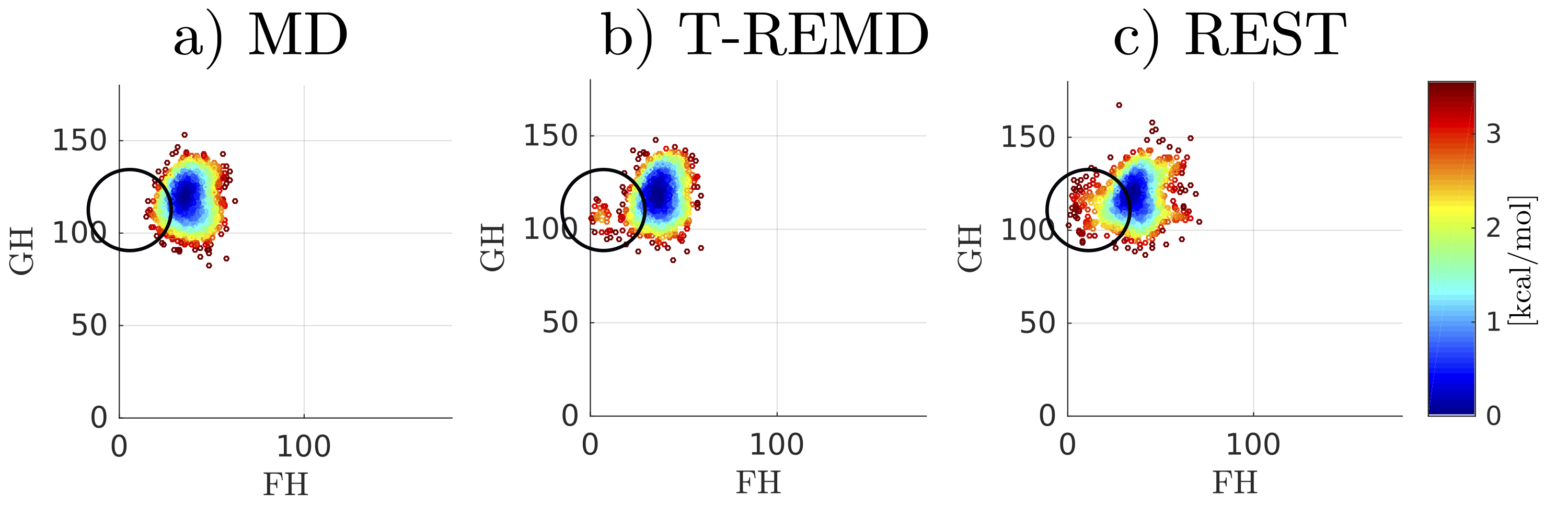

Supplement: S17 Fig — State 6 is marked with a ring, showing that it is only observed in temperature enhanced MD. (TIFF) [file pcbi.1006072.s017.tiff]

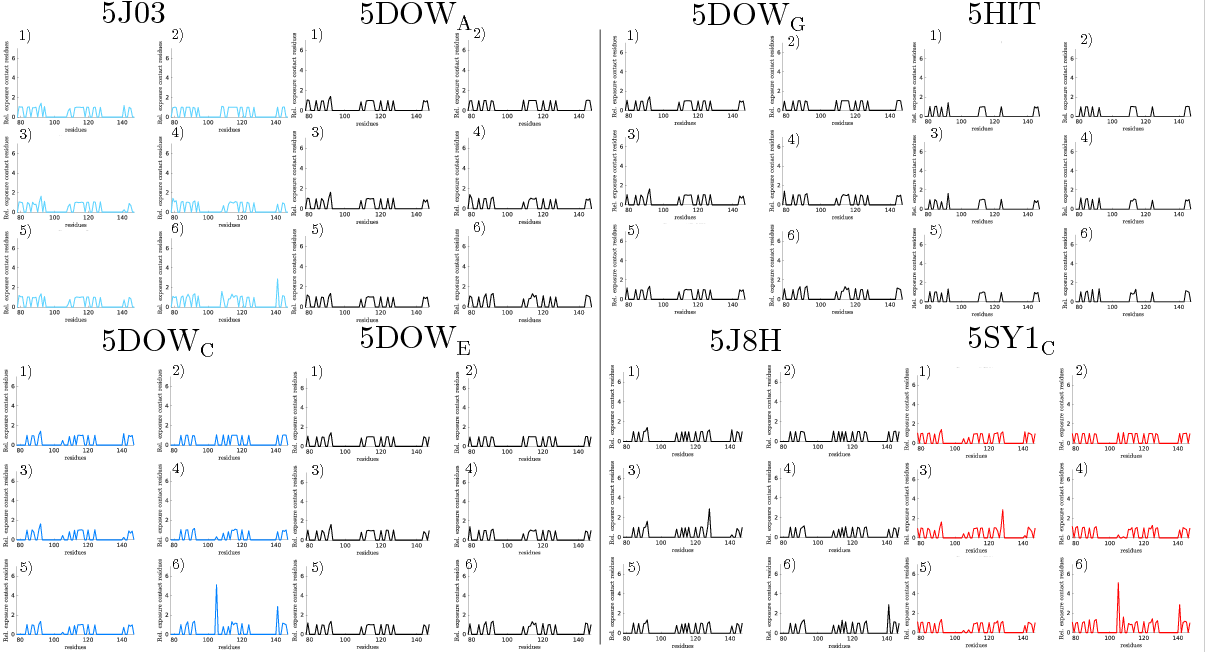

Supplement: S18 Fig — (TIFF) [file pcbi.1006072.s018.tiff]

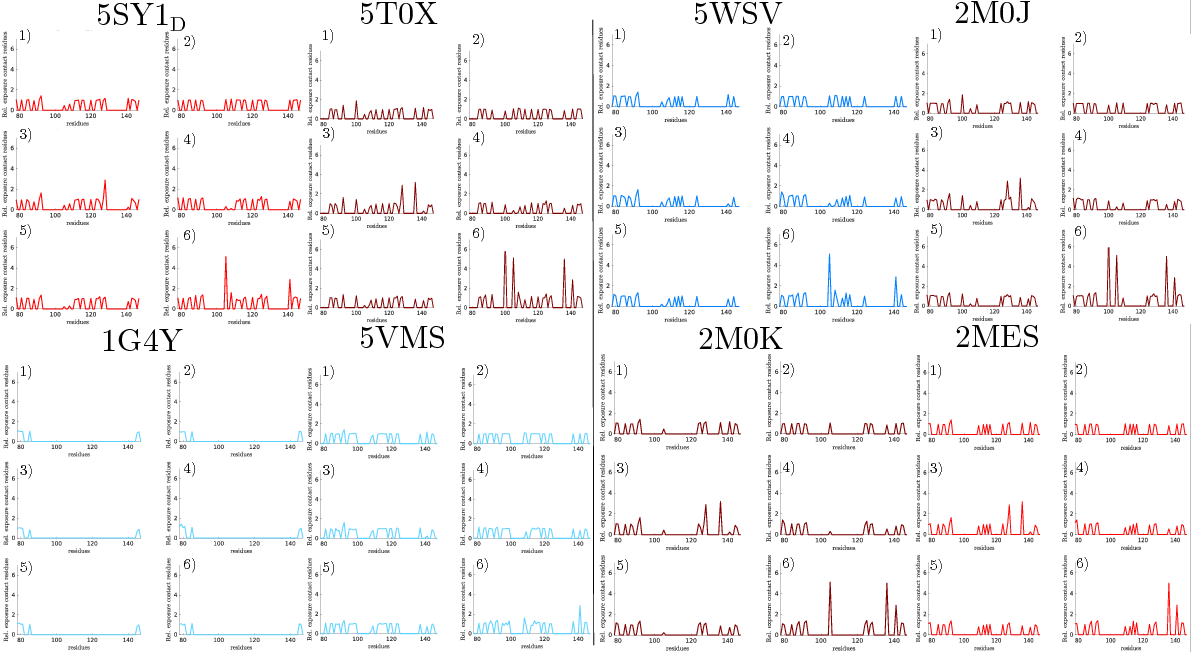

Supplement: S19 Fig — (TIFF) [file pcbi.1006072.s019.tiff]

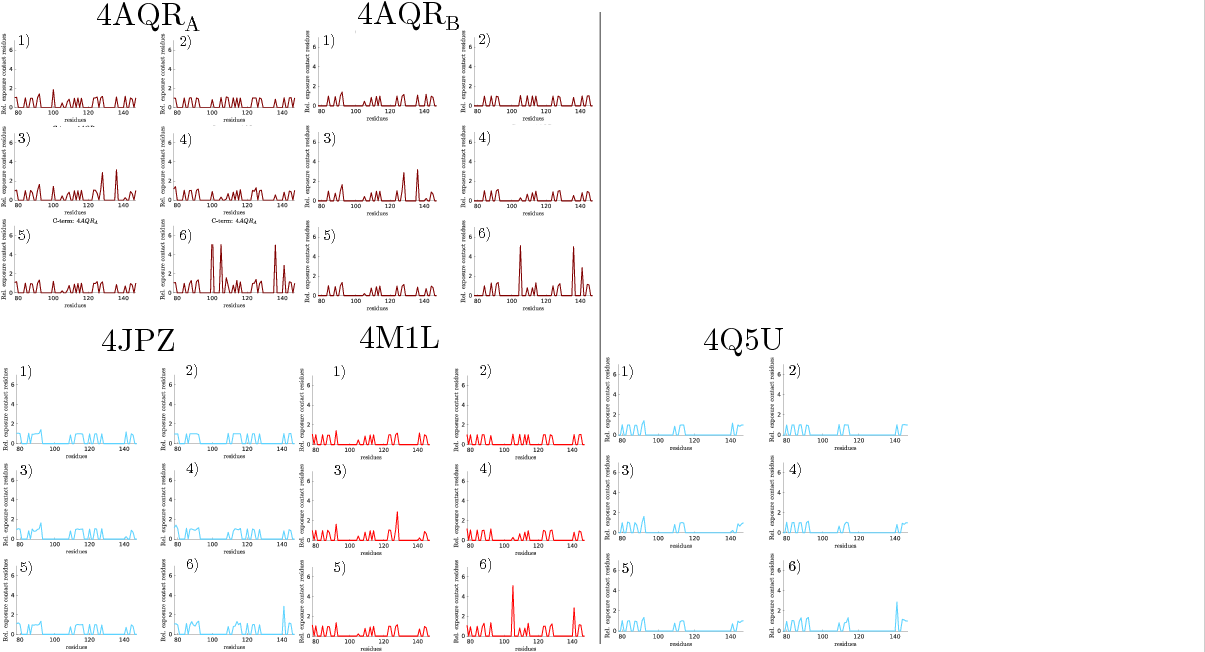

Supplement: S20 Fig — (TIFF) [file pcbi.1006072.s020.tiff]

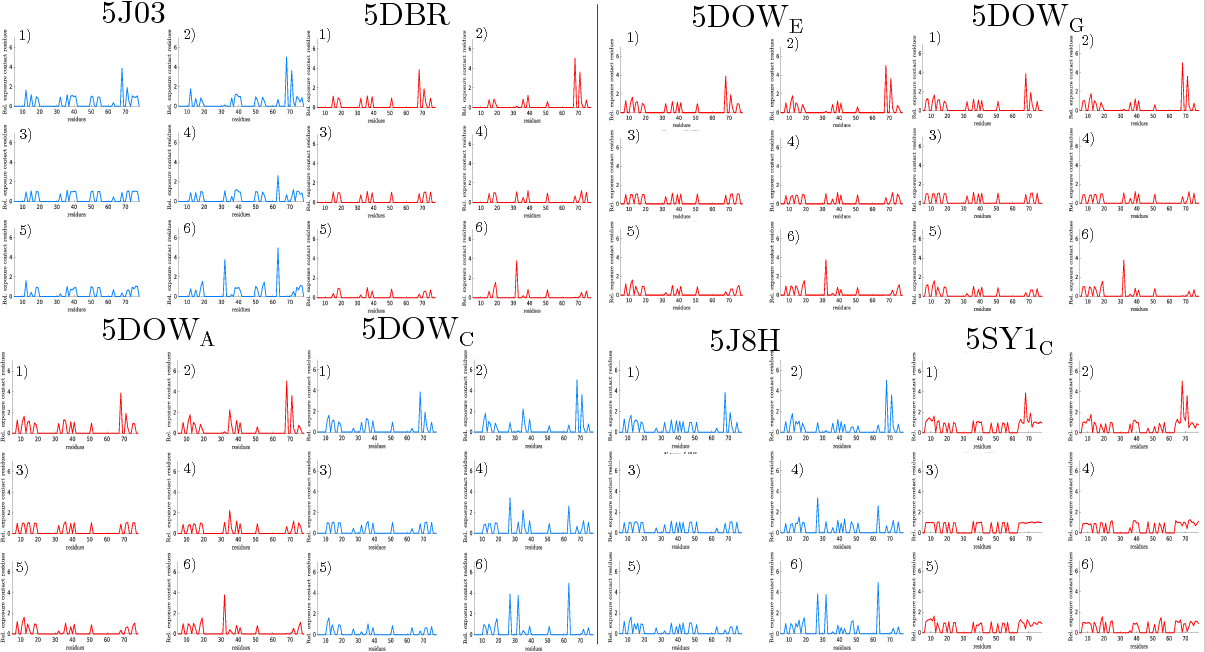

Supplement: S23 Fig — (TIFF) [file pcbi.1006072.s023.tiff]

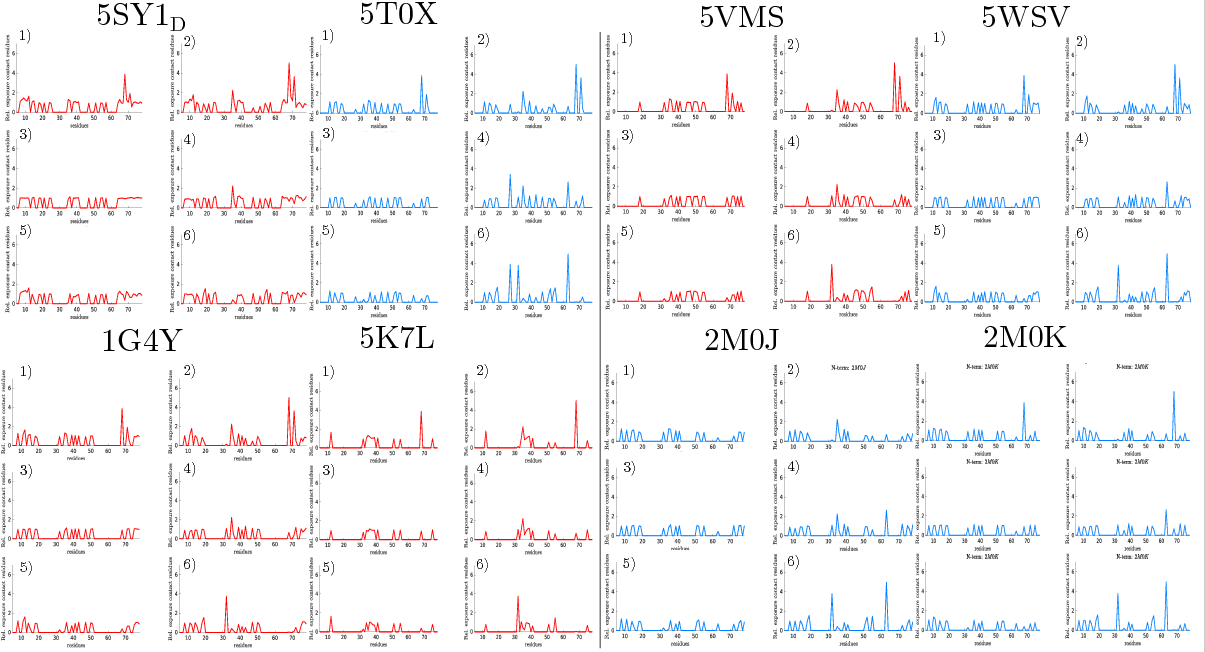

Supplement: S24 Fig — (TIFF) [file pcbi.1006072.s024.tiff]

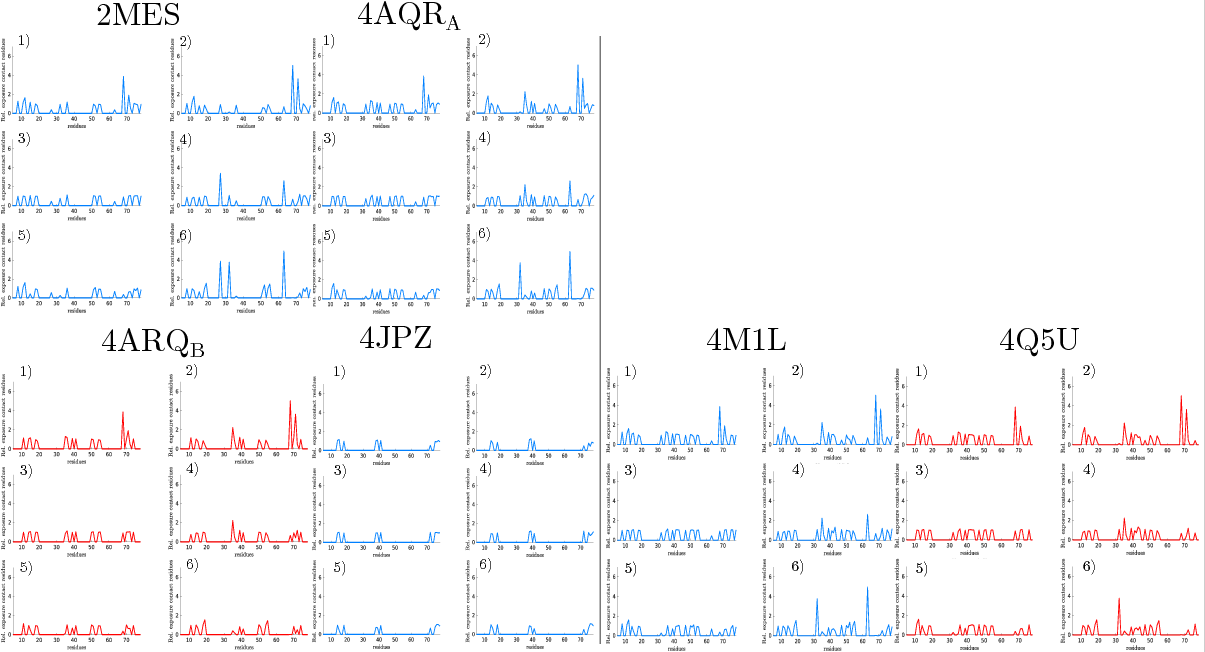

Supplement: S25 Fig — (TIFF) [file pcbi.1006072.s025.tiff]

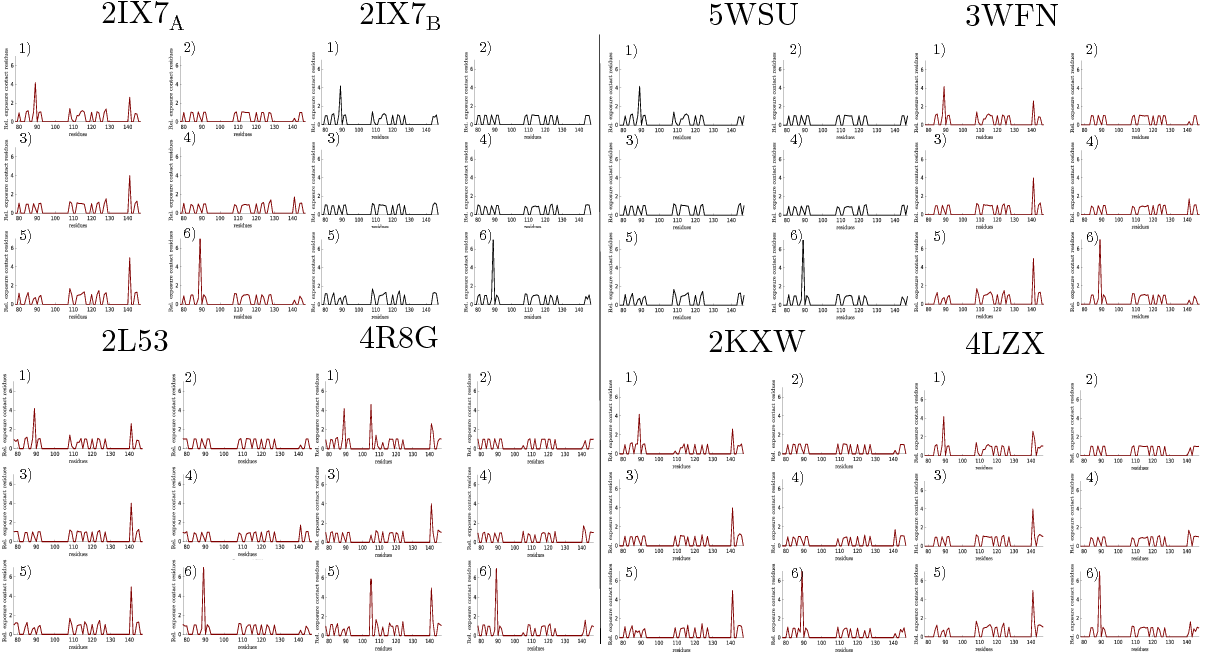

Supplement: S27 Fig — (TIFF) [file pcbi.1006072.s027.tiff]

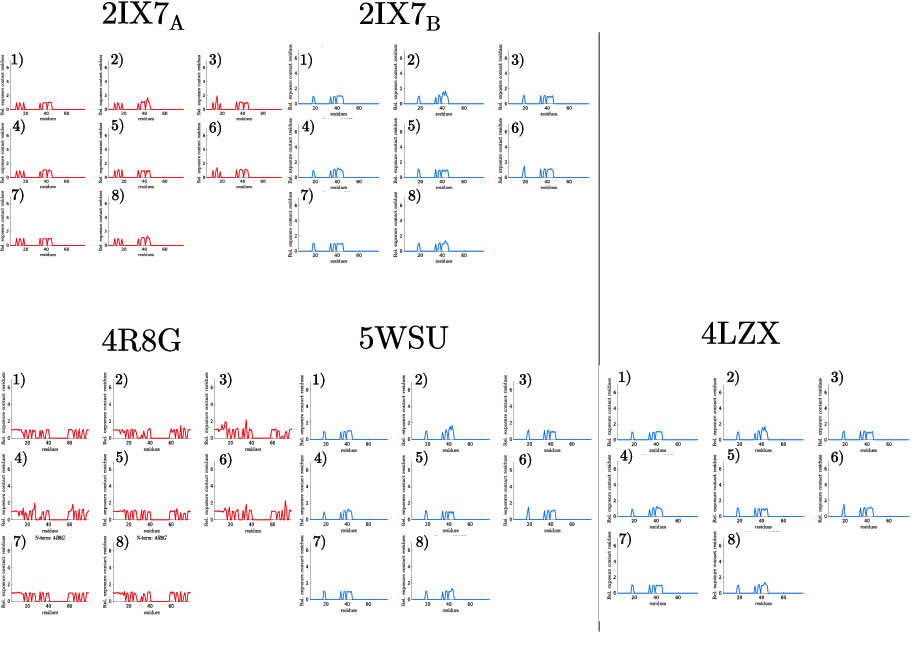

Supplement: S29 Fig — (TIFF) [file pcbi.1006072.s029.tiff]
