# Supplementary material for: Effect of Ca2+ on the promiscuous target-protein binding of calmodulin
Source: PLoS Comput Biol. 2018 Apr 3;14(4):e1006072. doi: 10.1371/journal.pcbi.1006072 (PMC5898786; doi:10.1371/journal.pcbi.1006072)
Supplement: S1 Text — A discussion of derived rate constants is also provided. (PDF) [file pcbi.1006072.s031.pdf]

Supplementary material: Effect of Ca^{2+} on the promiscuous target-protein binding of calmodulin

Annie Westerlund, Lucie Delemotte

March 13, 2018

Supplementary results

The representative structures of each state and a few similar experimental structures are shown in Fig S1- S4. The holo C-CaM state 3, Fig S1, corresponds to the helical arrangement found in 3CLN. Compared to this, state 2 has slightly smaller EH and larger EG angle, which correspond to a slight upward tilt of helix G and helix H C-term towards the linker. This is a similar helical arrangement as that of 5HIT. In addition to this, we note that interhelical angles are degenerate as descriptors for conformational states and thus their free energies. For example, holo N-CaM state 4 and 5SY1_C share the same ABC angles, Fig S2, but display different arrangements of helices. Thus, contacts are better suited for clustering the conformational space (see methods).
Among the holo N-CaM representative structures, Fig S2, we found states that strikingly resembled experimental structures. State 3 has similar helical arrangements as many experimental structures including 1CLL and 3CLN. This arrangement is characterized by a smaller CD angle, which results in the helices approaching a parallel arrangement. State 6, instead features an AC angle closer to perpendicular. Helix C is here almost parallel to the linker.
Among the apo C-CaM states, Fig S3, state 2 is distinguished from the rest through smaller EG and larger GH angles. Here, helices F and G are placed in front of E and H, forming a compact conformation. In contrast to this, state 5 has a larger EG and smaller GH angle, which is the result of helix G facing away from E, opening the hydrophobic cleft.
The representative apo N-CaM structures, Fig S4, display the resemblance between state 7 and 1DMO, where helix C is perpendicular to D, pointing in opposite directions, towards the reader. States 4, 6 and 8 have a larger AB angle than the other states. On these representative structures, the A and B helices are almost parallel, compared to states 3 and 5, where the helices A and B deviate more from being parallel. The main differences between state 8 and 4/6 are the angles between helices AC and CD. The orientation of helix C makes states 4 and 6 similar to 5WSU and 3WFN, while state 8 is not.

Rate constants derived from the MD simulations, Fig S11, suggest that some states interconvert more rapidly than others. The rates *to* holo C-CaM state 2 are for example larger than rates *from* it, indicating a lower relative free energy. The same argument also applies to apo N-CaM state 6. Similarly, the rates suggest that apo C-CaM state 5 and holo C-CaM state 5 have higher relative free energies, and that holo N-CaM 4, 6, holo C-CaM 3, 6, apo N-CaM 4 and apo C-CaM 1 are separated by high free energy barriers.

Supplementary figures

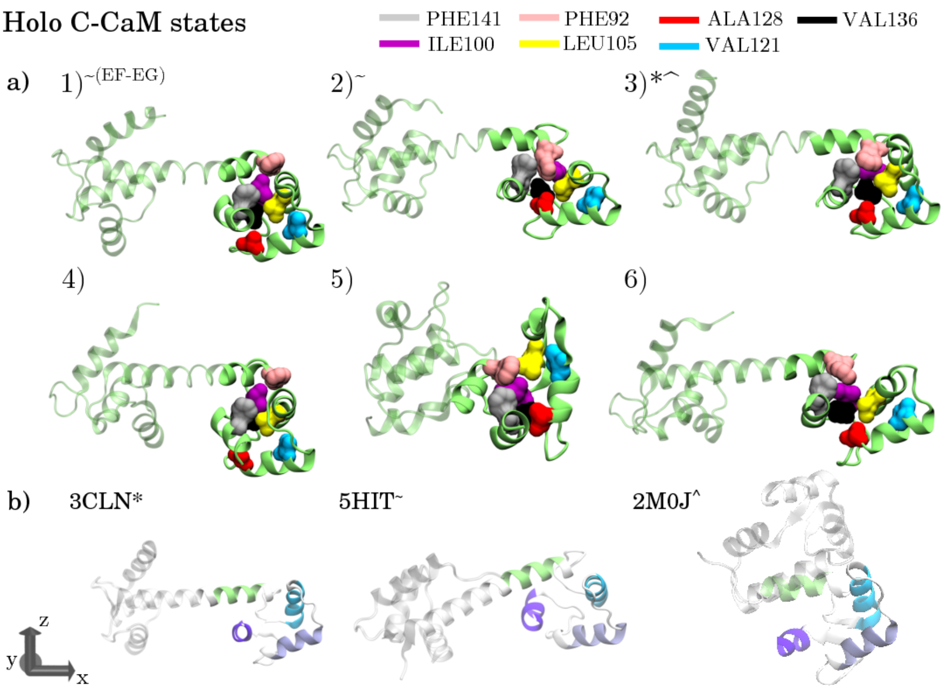

Figure S1: *a) The holo C-CaM states representative structures obtained from spectral clustering. Key residues from the contact/solvent exposure analysis are highlighted in colors. b) Experimentally obtained structures with similar interhelical angle arrangements as the obtained states. The states that are similar to experimentally obtained states are marked by a symbol corresponding to the experimental structure. Note that state 1 is similar to 5HIT only at the EF-EG angles.*

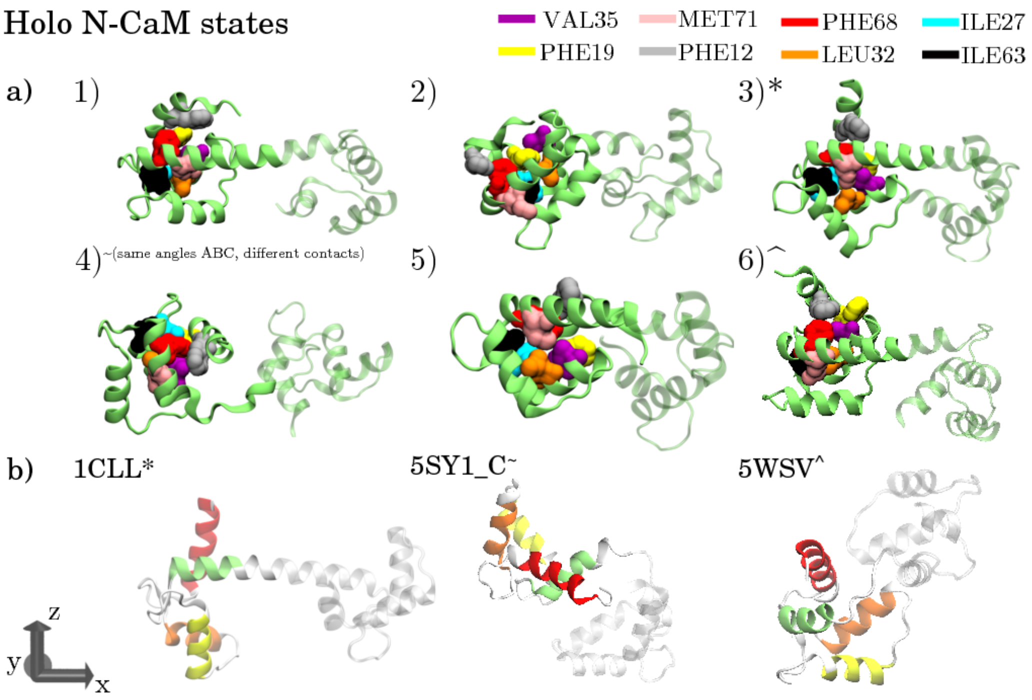

Figure S2: *a) The holo N-CaM states representative structures obtained from spectral clustering. Key residues from the contact/solvent exposure analysis are highlighted in colors. b) Experimentally obtained structures with similar interhelical angle arrangements as the obtained states. The states that are similar to experimentally obtained states are marked by a symbol corresponding to the experimental structure. Note that state 4 has similar interhelical angles as 5SY1_C but a different set of interresidue contacts.*

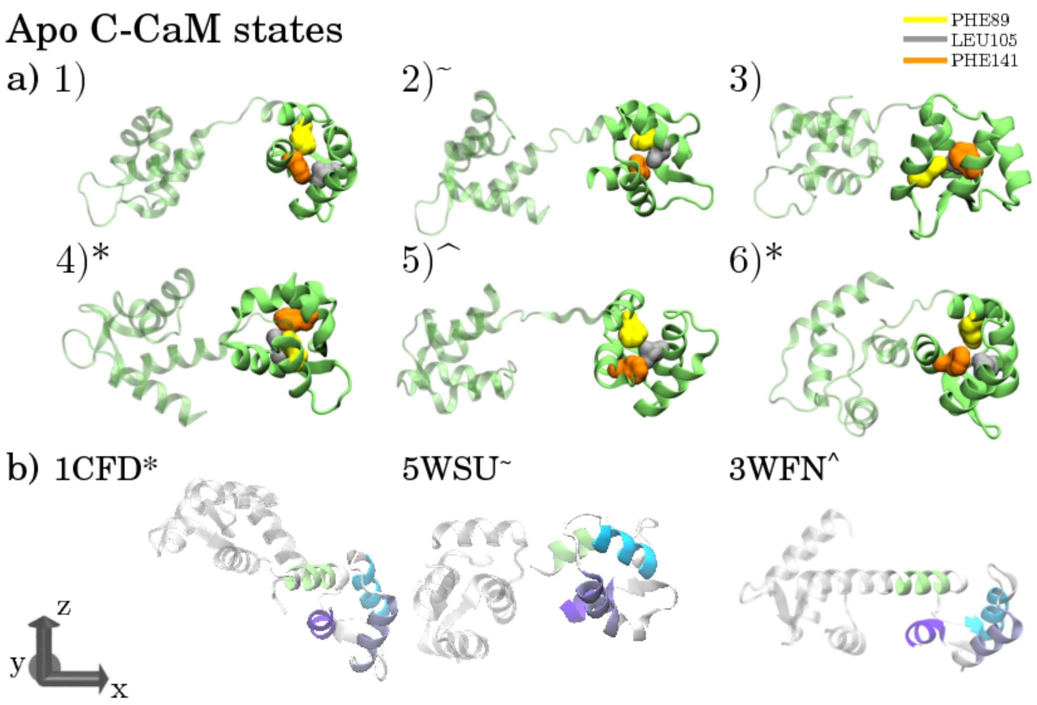

Figure S3: *a) The apo C-CaM states representative structures obtained from spectral clustering. Key residues from the contact/solvent exposure analysis are highlighted in colors. b) Experimentally obtained structures with similar interhelical angle arrangements as the obtained states. The states that are similar to experimentally obtained states are marked by a symbol corresponding to the experimental structure.*

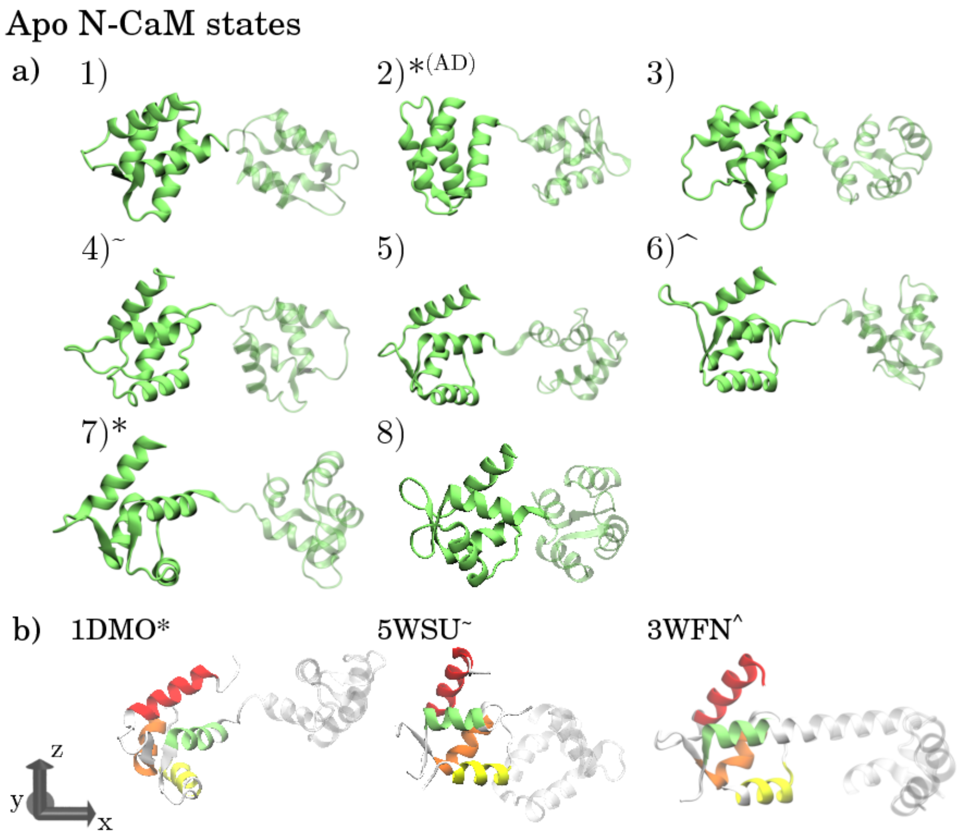

Figure S4: *a) The apo N-CaM states representative structures obtained from spectral clustering. b) Experimentally obtained structures with similar interhelical angle arrangements as the obtained states. The states that are similar to experimentally obtained states are marked by a symbol corresponding to the experimental structure. Note that state 2 is similar to 1DMO only for the AD angle.*

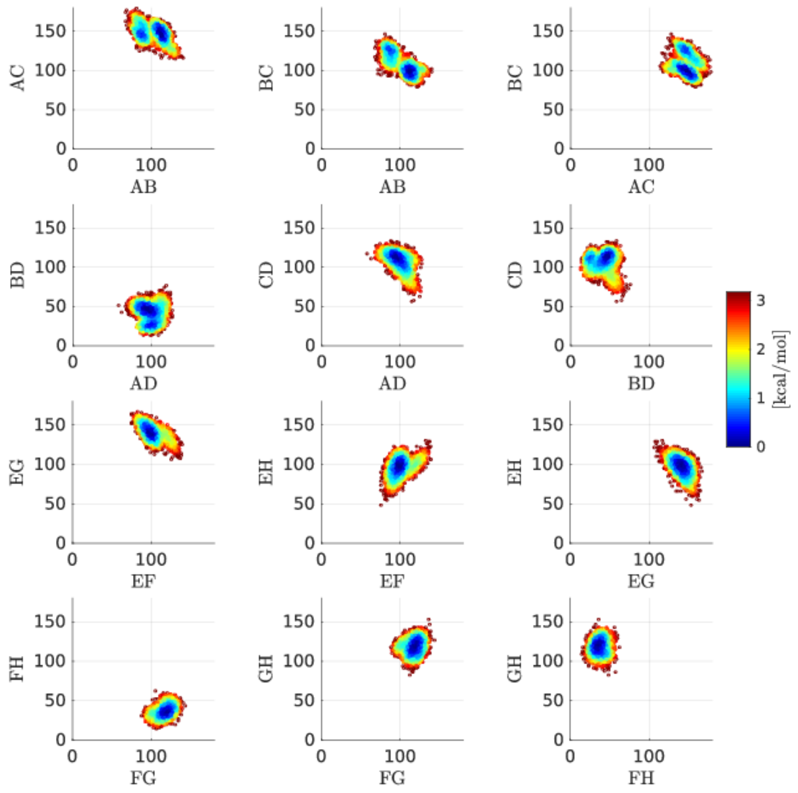

Figure S5: *Estimated free energy landscapes of the holo ensemble using data acquired from MD simulations. Note that these are not accurate estimates of free energies due to limited simulation time.*

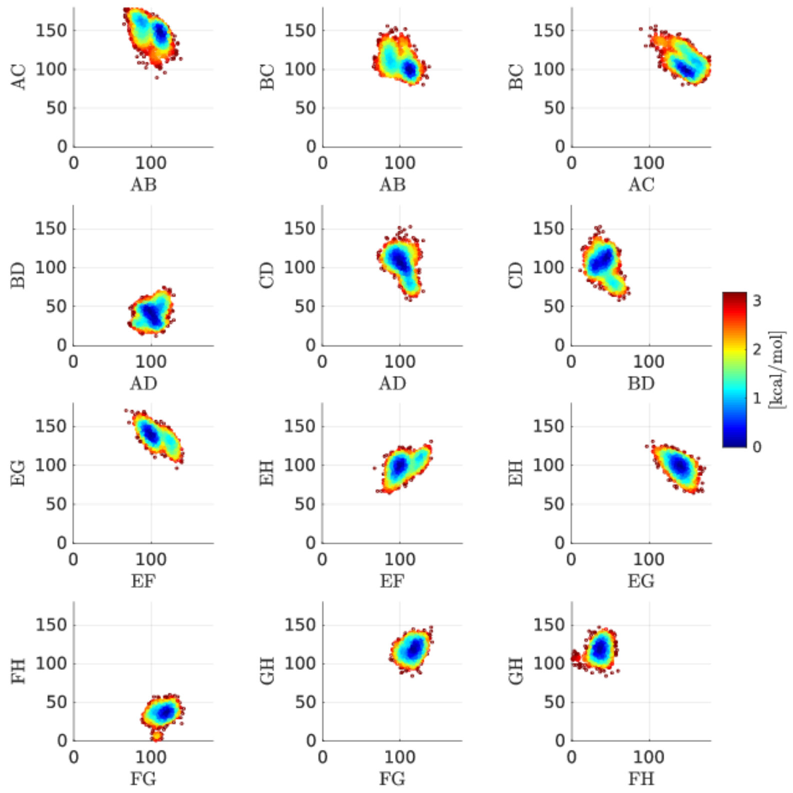

Figure S6: *Estimated free energy landscapes of the holo ensemble using data acquired from T-REMD simulations. Note that these are not accurate estimates of free energies due to limited simulation time.*

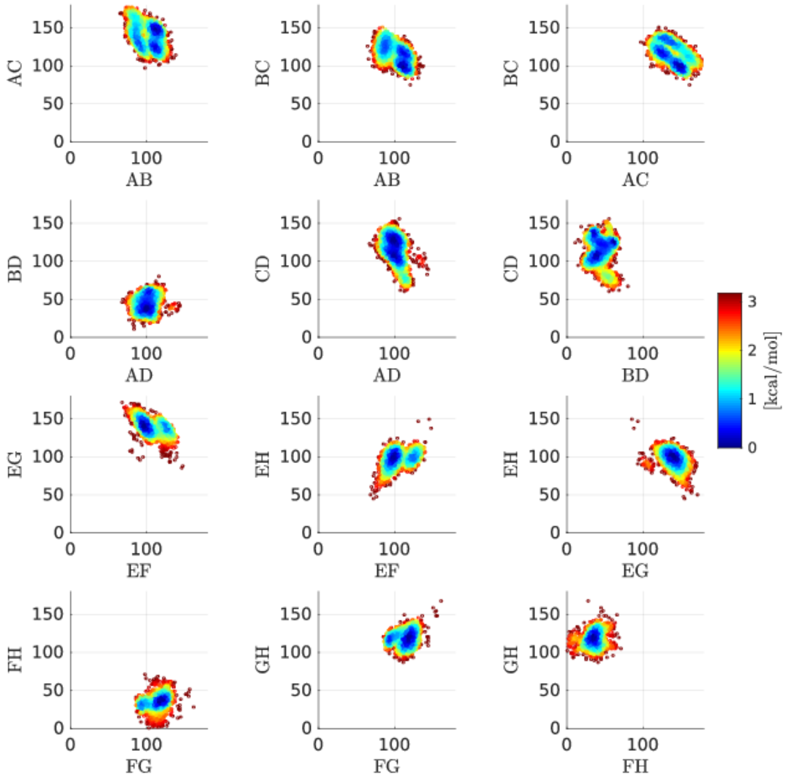

Figure S7: *Estimated free energy landscapes of the holo ensemble using data acquired from REST simulations. Note that these are not accurate estimates of free energies due to limited simulation time.*

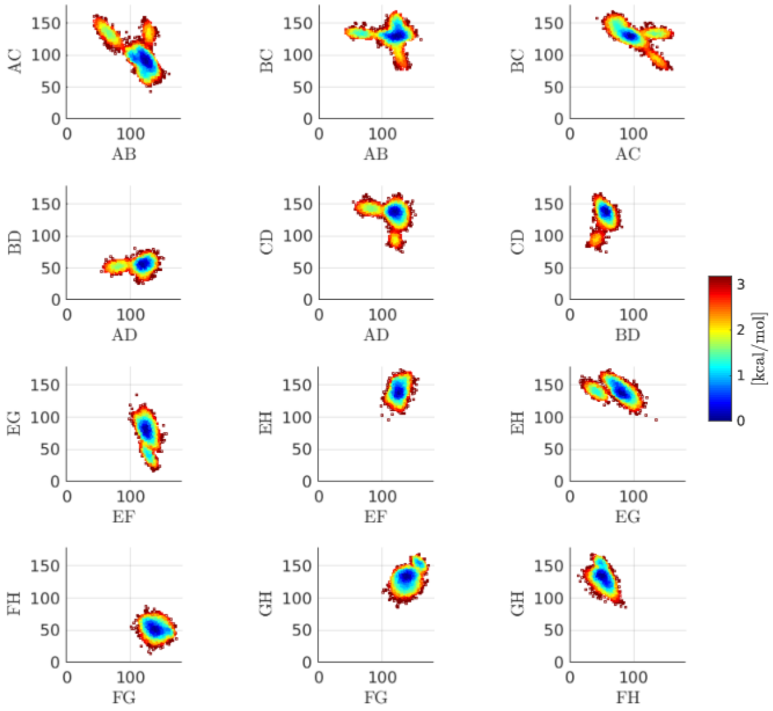

Figure S8: *Estimated free energy landscapes of the apo ensemble using data acquired from MD simulations. Note that these are not accurate estimates of free energies due to limited simulation time.*

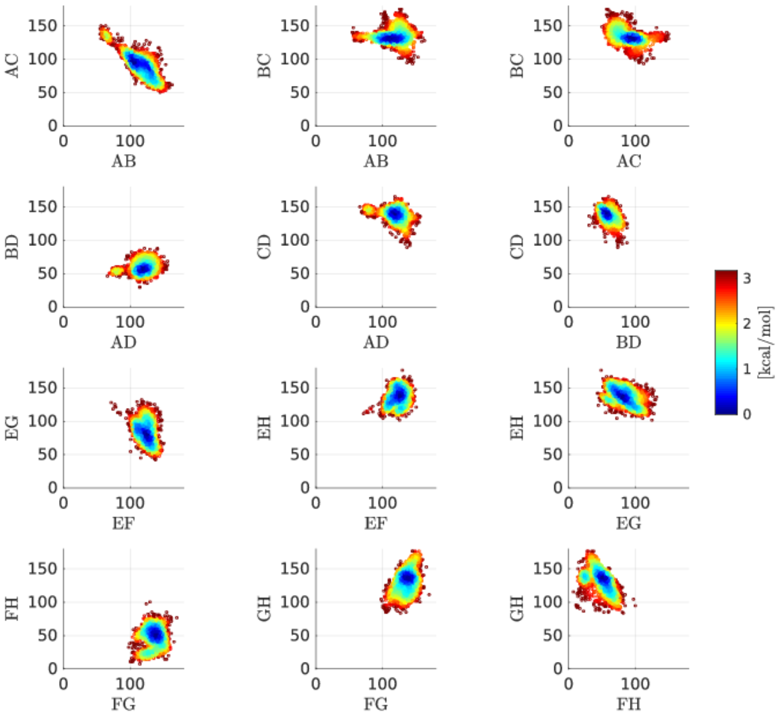

Figure S9: *Estimated free energy landscapes of the apo ensemble using data acquired from T-REMD simulations. Note that these are not accurate estimates of free energies due to limited simulation time.*

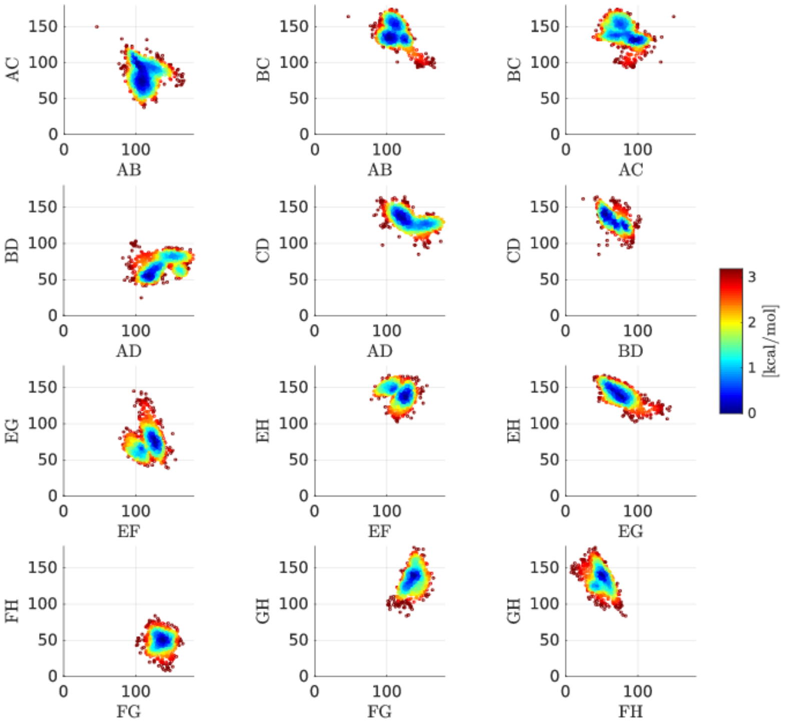

Figure S10: *Estimated free energy landscapes of the apo ensemble using data acquired from REST simulations. Note that these are not accurate estimates of free energies due to limited simulation time.*

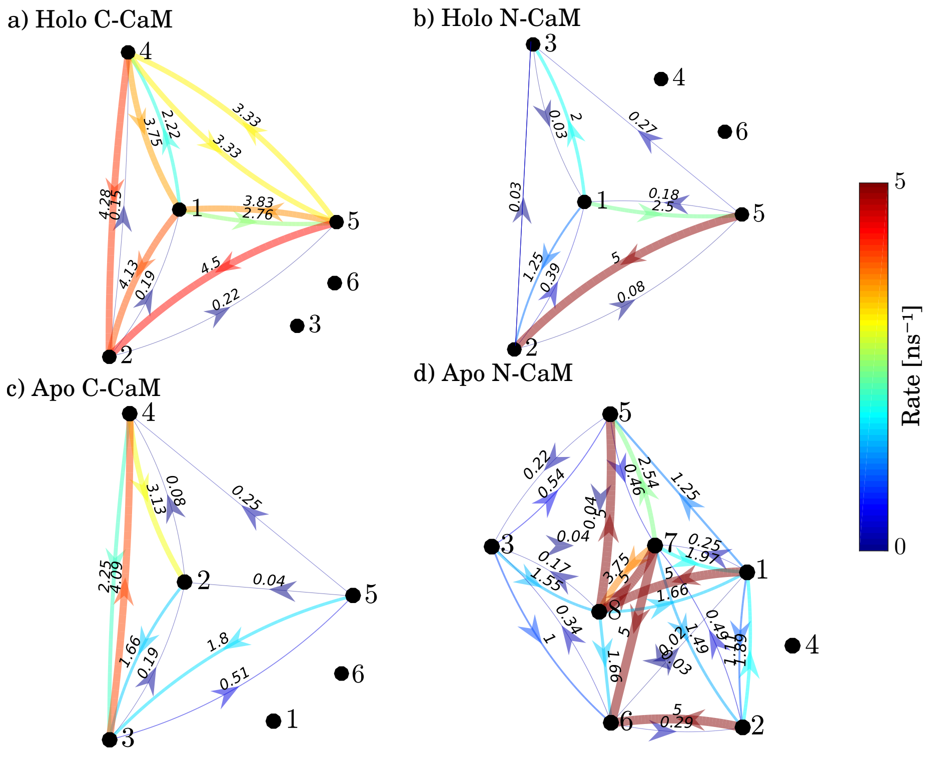

Figure S11: *Networks of interconversion between the states obtained in this study, as derived from the MD simulation trajectories. States that were not sampled in the plain MD simulations are disconnected from the networks. Replica exchange simulations disrupt the dynamics through coordinate exchanges. Although kinetics of toy systems can be restored from replica exchange simulations [1], application of the method to the present dataset was not possible.*

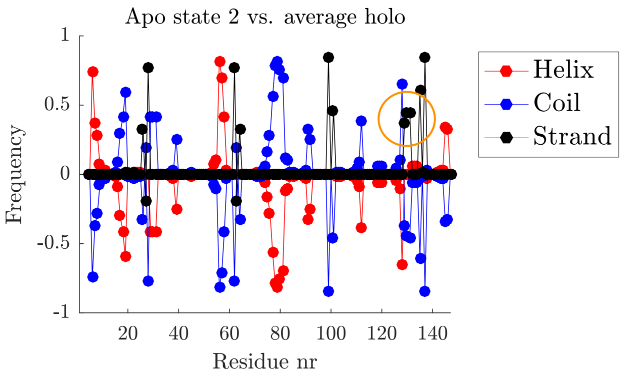

Figure S12: *Secondary structure frequency of apo C-CaM state 2 difference to average holo secondary structure frequency. The propensity of residues 129-131 to join the beta sheet and deforming the fourth Ca^{2+}-loop is marked by an orange circle.*

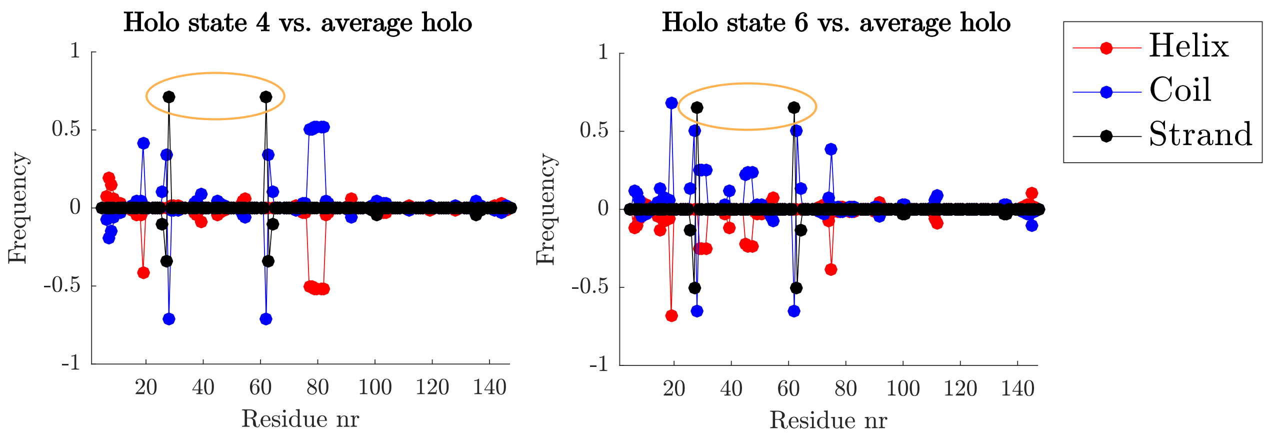

Figure S13: *Secondary structure frequency of holo N-CaM state 4 and 6 difference to average holo secondary structure frequency. These states overlap the apo ensemble. The beta sheet shift to residue 28/62 in N-CaM is marked by an orange ellipse.*

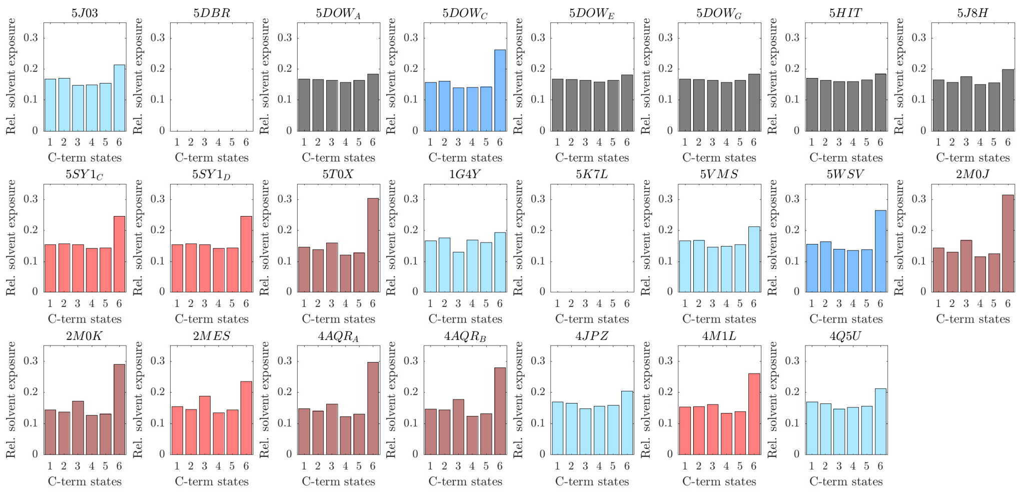

Figure S14: *Holo C-CaM total relative solvent exposure for each CaM-complex structure.*

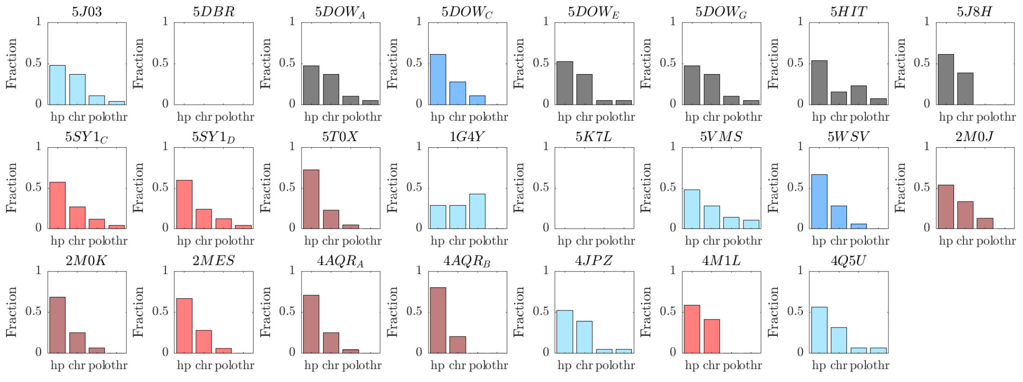

Figure S15: *The residue types involved in holo C-CaM target protein contacts.*

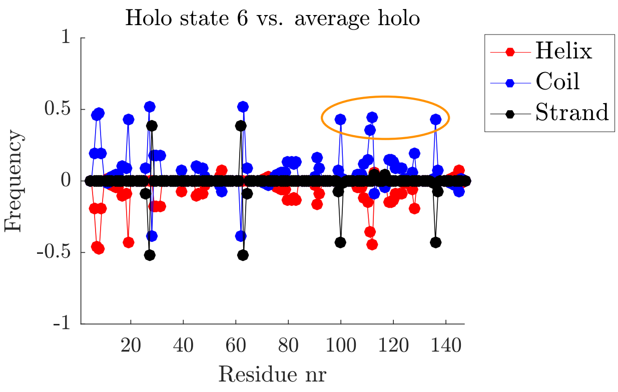

Figure S16: *Secondary structure frequency of holo state 6 difference to average holo secondary structure frequency. The lack of beta sheets in the C-term lobe is marked by an orange ellipse.*

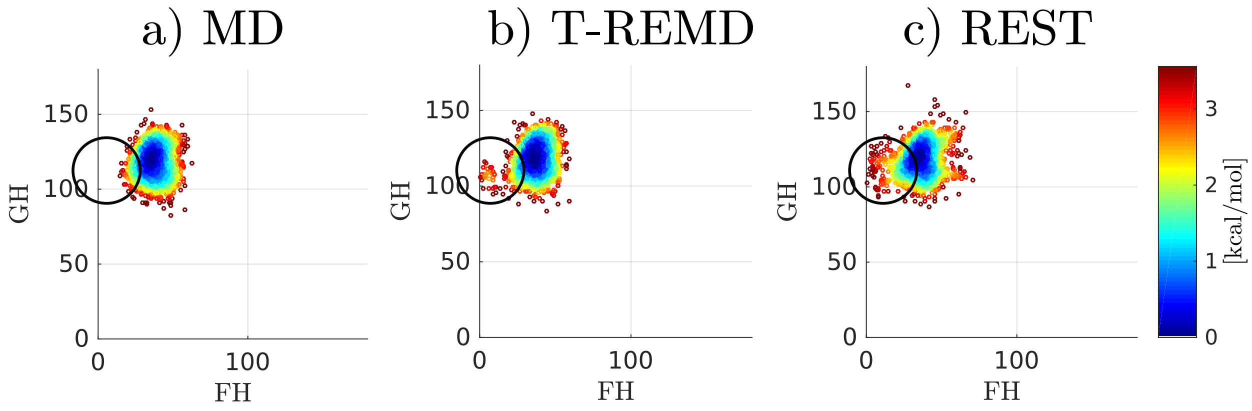

Figure S17: *Estimated free energy landscapes over holo FH and GH inter-helical angles using GMM with cross validation free energy estimator [2]. State 6 is marked with a ring, showing that it is only observed in temperature enhanced MD.*

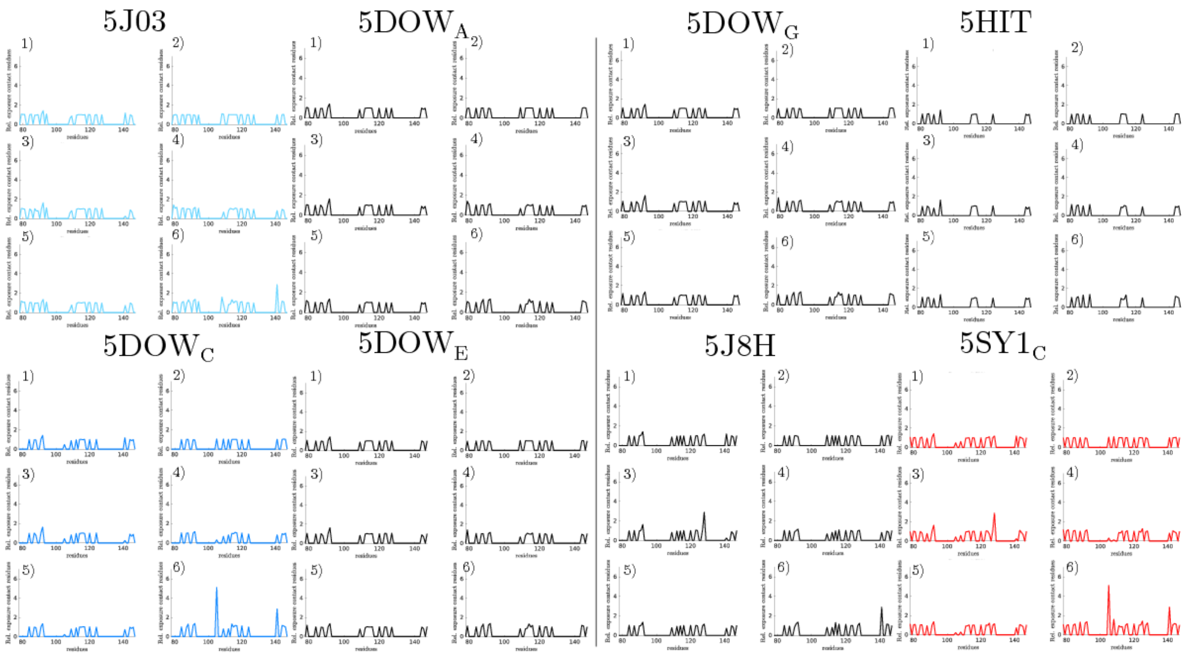

Figure S18: *The relative solvent exposure per CaM-complex contact residue for the different holo C-CaM states.*

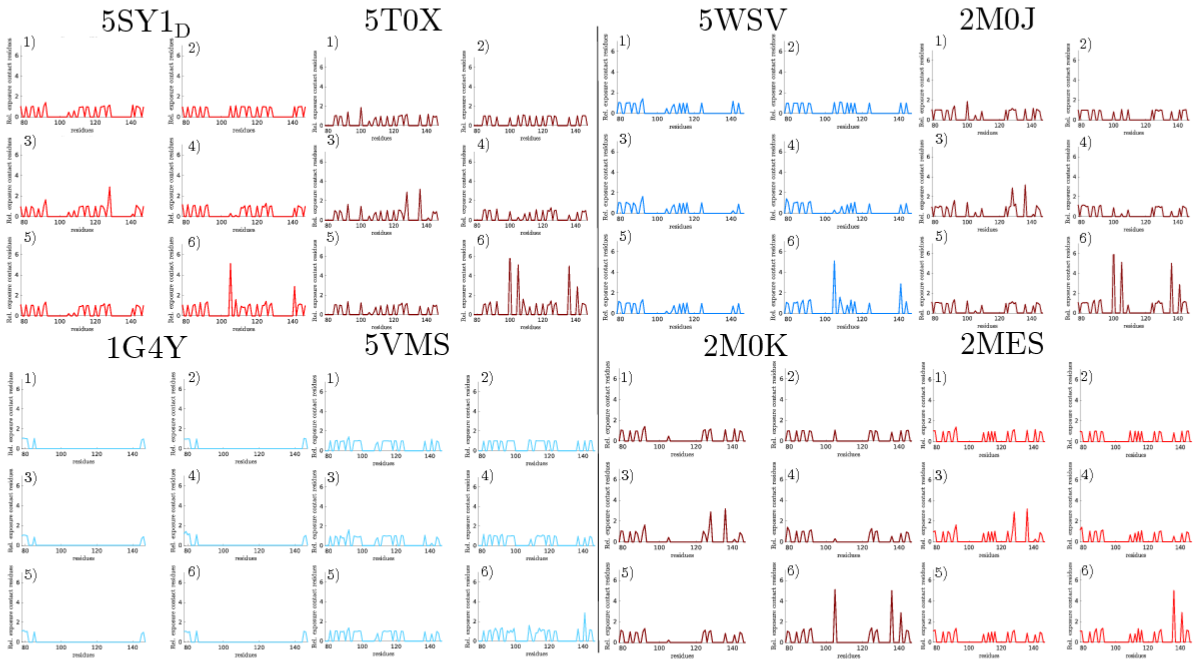

Figure S19: *The relative solvent exposure per CaM-complex contact residue for the different holo C-CaM states.*

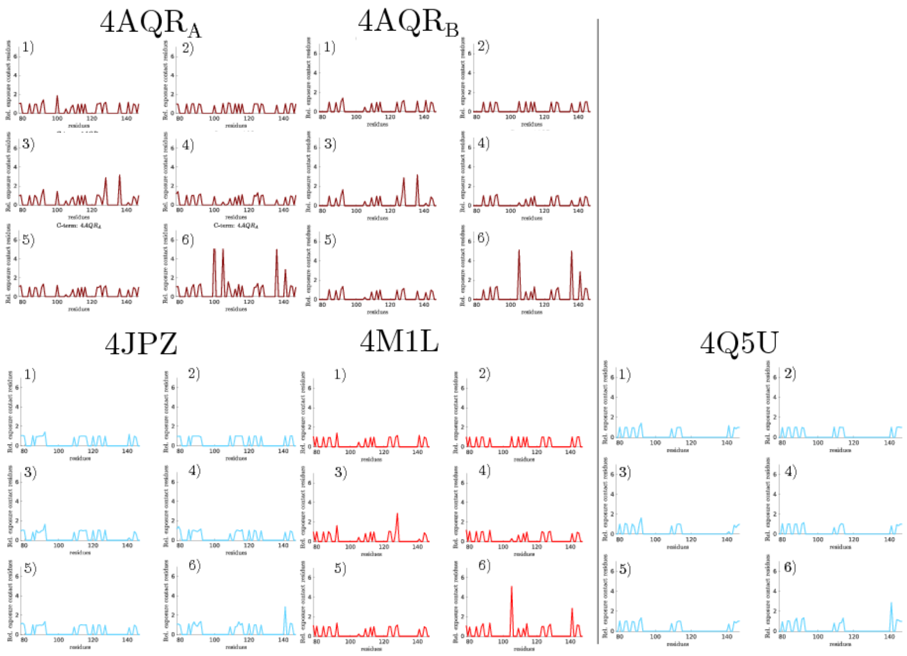

Figure S20: *The relative solvent exposure per CaM-complex contact residue for the different holo C-CaM states.*

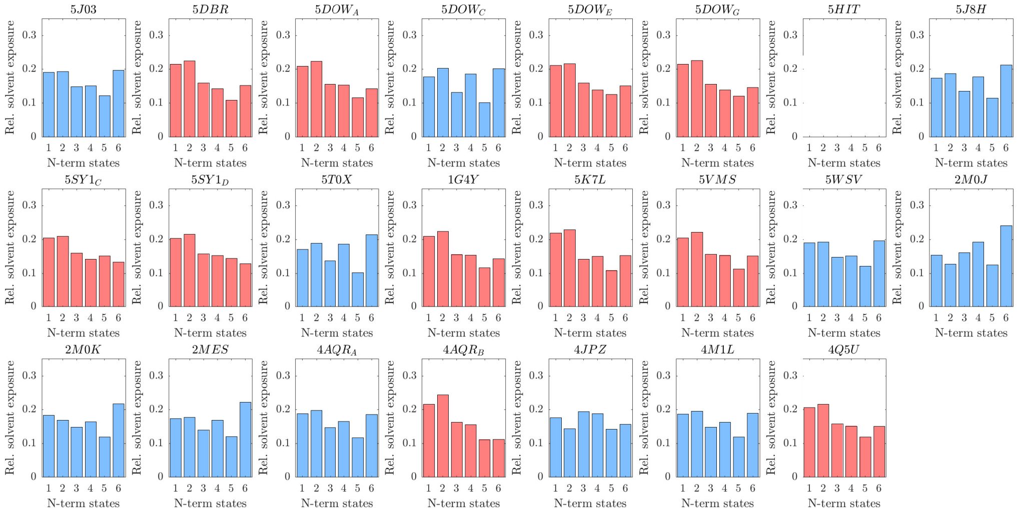

Figure S21: *Holo N-CaM total relative solvent exposure for each CaM-complex structure.*

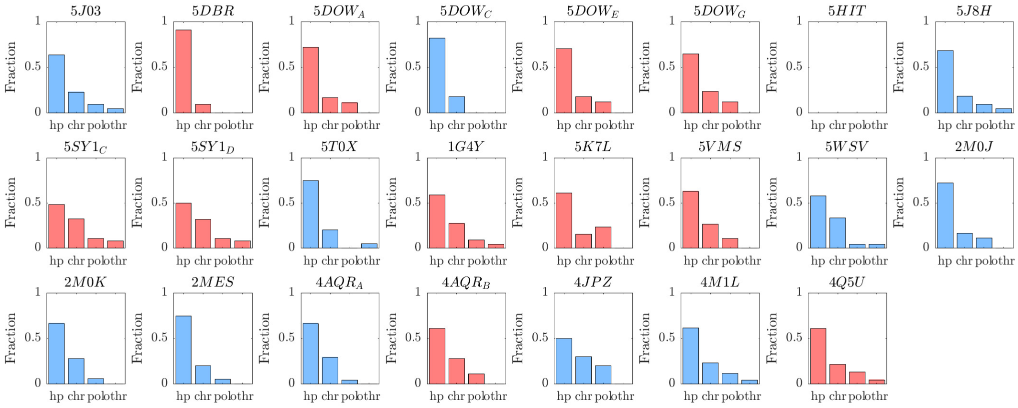

Figure S22: *The residue types involved in holo N-CaM target protein contacts for each CaM-complex structure.*

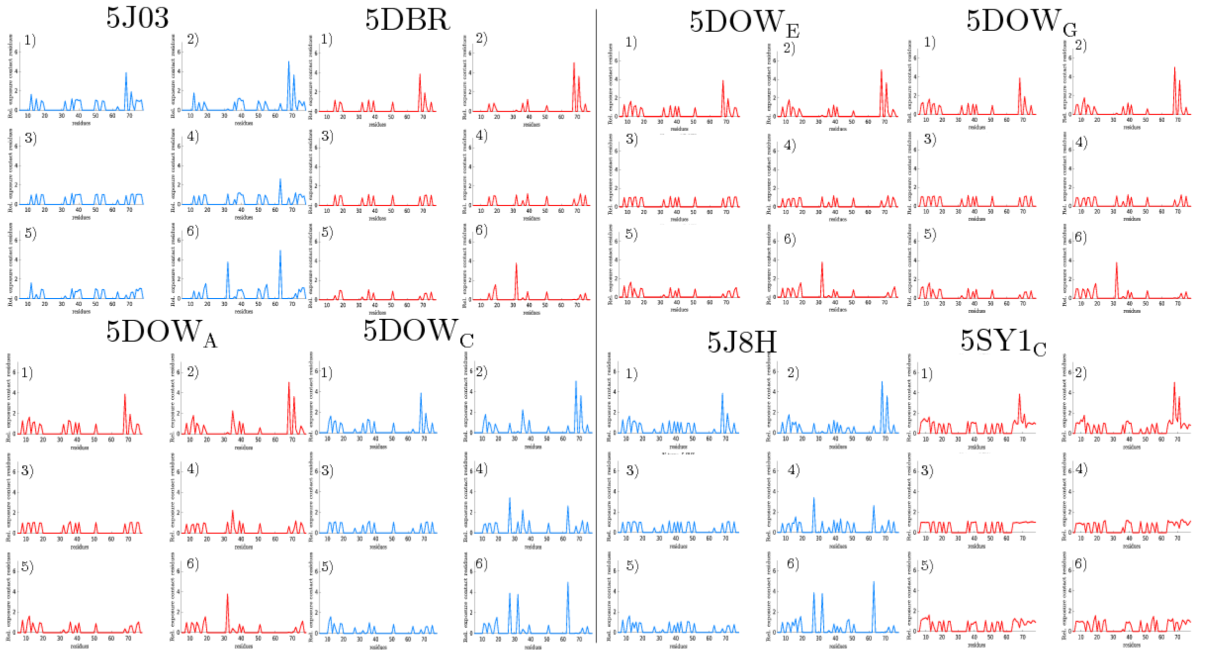

Figure S23: *The relative solvent exposure per CaM-complex contact residue for the different holo N-CaM states.*

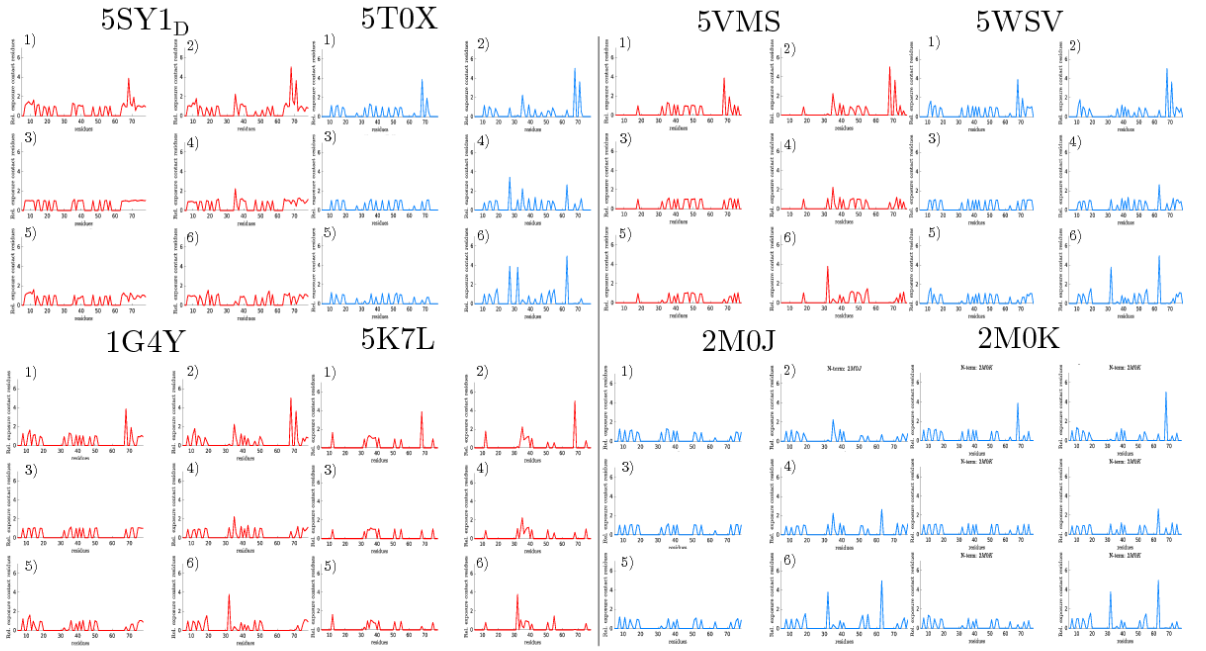

Figure S24: *The relative solvent exposure per CaM-complex contact residue for the different holo N-CaM states.*

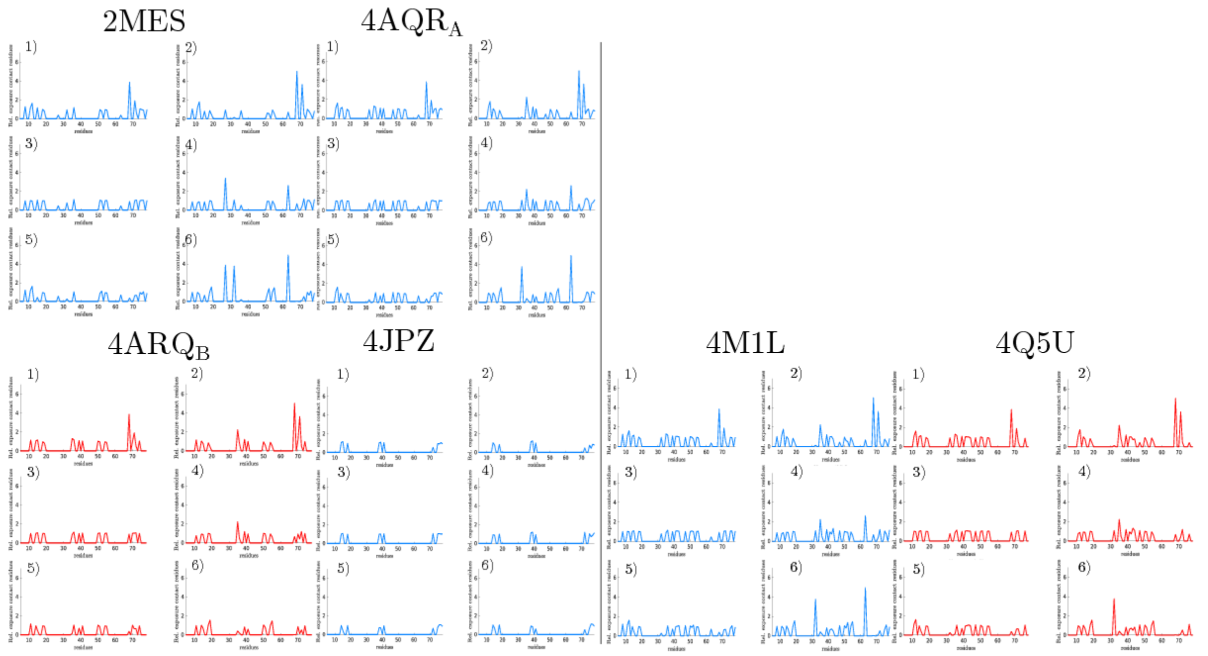

Figure S25: *The relative solvent exposure per CaM-complex contact residue for the different holo N-CaM states.*

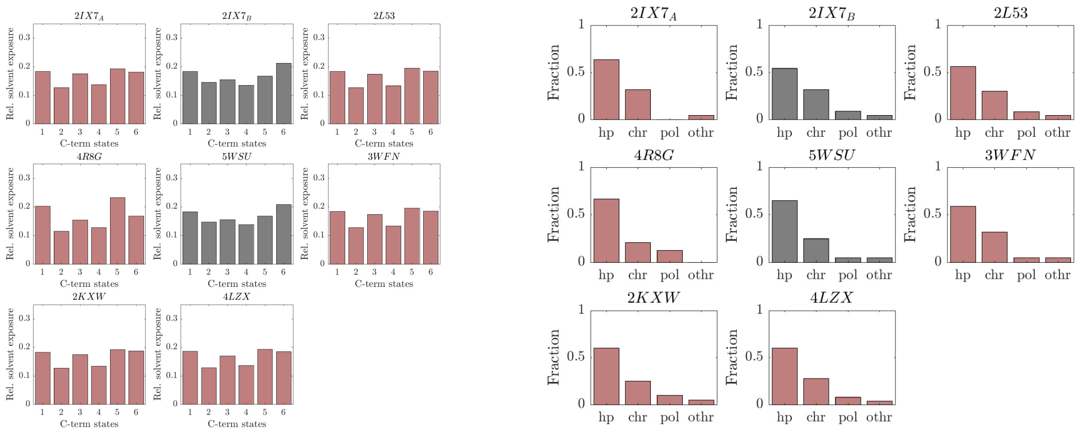

Figure S26: *Left: Apo C-CaM total relative solvent exposure for each CaM-complex structure. Right: The residue types involved in apo C-CaM target protein contacts.*

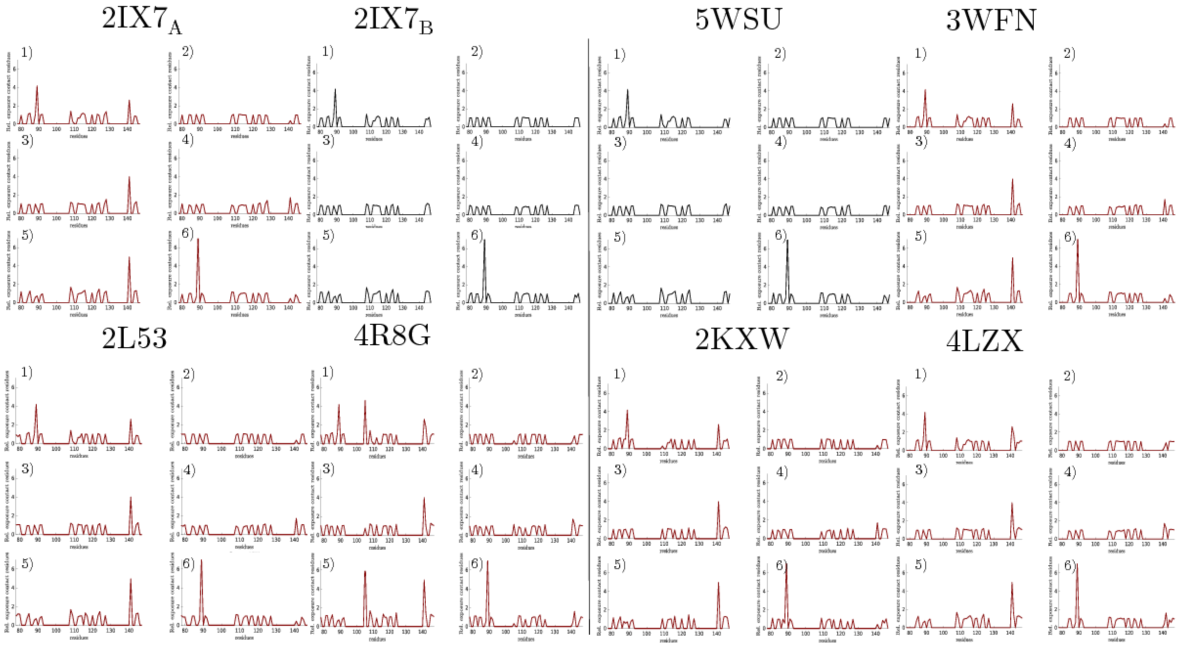

Figure S27: *The relative solvent exposure per CaM-complex contact residue for the different apo C-CaM states.*

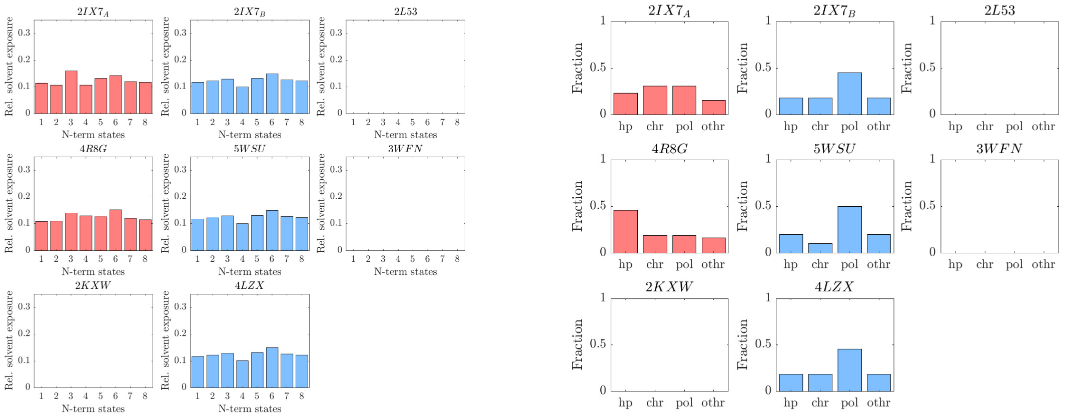

Figure S28: *Left: Apo N-CaM total relative solvent exposure for each CaM-complex structure. Right: The residue types involved in apo N-CaM target protein contacts.*

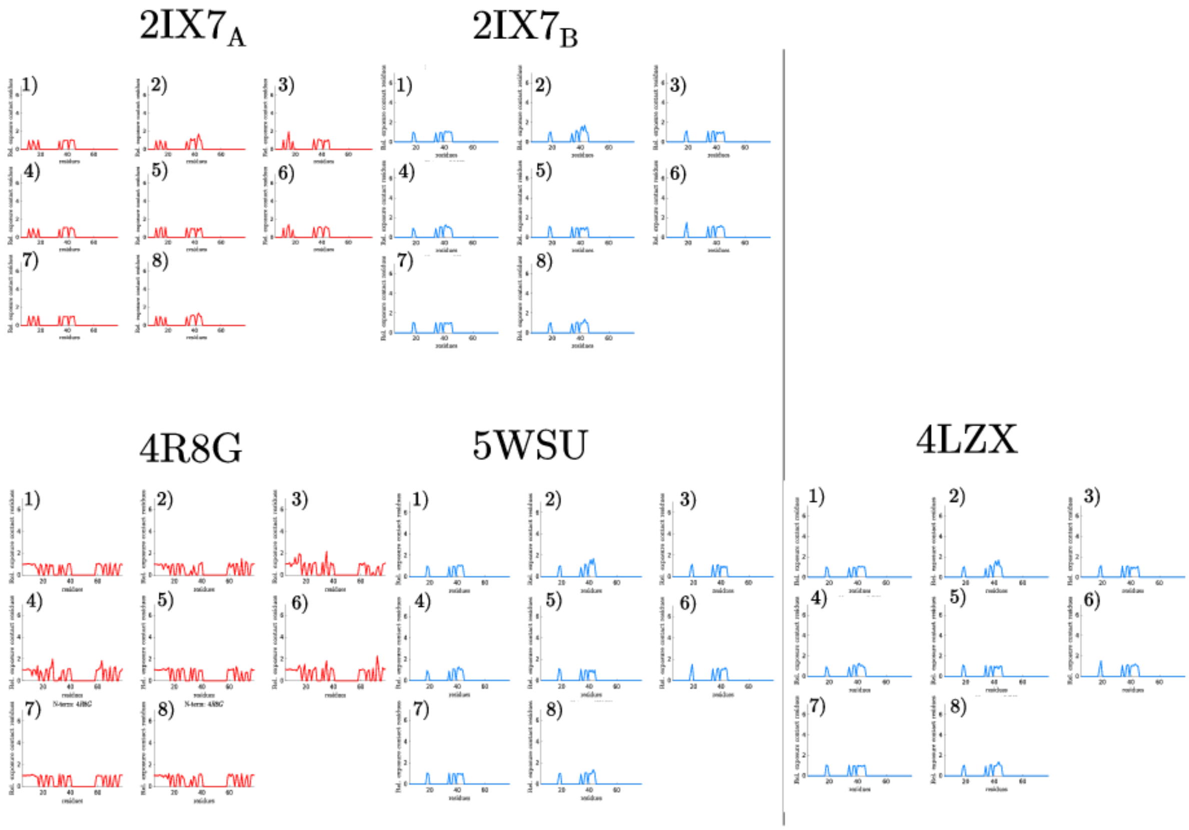

Figure S29: *The relative solvent exposure per CaM-complex contact residue for the different apo N-CaM states.*

References

[1] Lukas S. Stelzl and Gerhard Hummer. Kinetics from Replica Exchange Molecular Dynamics Simulations. *Journal of Chemical Theory and Computation*, 13(8):3927–3935, August 2017.

[2] Annie M. Westerlund, Tyler J. Harpole, Christian Blau, and Lucie Delemotte. Inference of Calmodulin's Ca2+-Dependent Free Energy Landscapes via Gaussian Mixture Model Validation. *Journal of Chemical Theory and Computation*, November 2017.
